# Site-specific recombination and purifying selection influence Cry toxin formation and specialization

**DOI:** 10.1093/ismejo/wrag218

**Published:** 2026-08-26

**Authors:** Anton E Shikov, Ruslan O Alagov, Maria E Belousova, Anton A Nizhnikov, Kirill S Antonets

**Affiliations:** Faculty of Biology, St. Petersburg State University, St. Petersburg 199034, Russia; Laboratory for Proteomics of Supra-Organismal Systems, All-Russia Research Institute for Agricultural Microbiology, St. Petersburg 196608, Russia; Laboratory for Proteomics of Supra-Organismal Systems, All-Russia Research Institute for Agricultural Microbiology, St. Petersburg 196608, Russia; Laboratory for Proteomics of Supra-Organismal Systems, All-Russia Research Institute for Agricultural Microbiology, St. Petersburg 196608, Russia; Faculty of Biology, St. Petersburg State University, St. Petersburg 199034, Russia; Laboratory for Proteomics of Supra-Organismal Systems, All-Russia Research Institute for Agricultural Microbiology, St. Petersburg 196608, Russia; Faculty of Biology, St. Petersburg State University, St. Petersburg 199034, Russia; Laboratory for Proteomics of Supra-Organismal Systems, All-Russia Research Institute for Agricultural Microbiology, St. Petersburg 196608, Russia

**Keywords:** Cry toxins, *Bacillus thuringiensis*, recombination, domain swapping, host specificity, purifying selection, MGEs, insertion sequences, conserved regions

## Abstract

In bacteria, multi-domain toxins represent a universal tool for determining the host range, being a cornerstone for public health and agriculture. Given high host specificity and potency, Cry toxins represent the most widely used bioinsecticides in agriculture. Their worldwide application grows annually; thus, the rational design of bioinsecticides requires a deeper understanding of the processes that orchestrate the origin and evolution of Cry toxins. The accepted views on how Cry toxins exchange domains postulate that homologous recombination sparks domain III swapping, altering host specificity and broadening strains’ activities. This perspective, however, stems from laboratory experiments and has never been studied in nature. Here, we leveraged all available Cry sequences and uncovered the mechanism of the evolution of Cry toxins via site-specific recombination guided by XerCD recombinases that target insertions and conserved blocks between domain borders. The emergence of specialists from generalists is sparked by the purifying selection of specialized hybrids. Therefore, our model of domain shuffling caused by site-specific recombination of *cry* loci, followed by selection-driven specialization, illustrates how multi-domain toxins evolve and attune to specific hosts.

## Introduction

Adaptation of bacterial pathogens strictly relies on attuning their toxic moieties. This process is a double-edged sword, leading to both broadening the activities and narrowing the affected hosts in the case of highly competitive environments [1]. The trajectory of crystalline Cry toxins in this context remains understudied, despite their overwhelming agricultural significance. Cry toxins are ubiquitous and the most widely used biopesticides, predominantly produced by *Bacillus thuringiensis* (*Bt*), accounting for 90%–97% of the biocontrol agents in pest management [2, 3]. The worldwide application of transgenic crops modified with *Bt* genes has risen from 32 to 191 million hectares by 2018 [4, 5]. The biopesticides market is expected to reach nearly equivalent market sizes for chemical pesticides by the 2040s–2050s [6, 7]. Despite considerable progress, the emergence of resistant pests requires the development of novel toxins [8–10].

Cry toxins are considered major virulence factors in certain bacteria apart from *Bt*, such as *Clostridium* and *Lysinibacillus* [11]. After reclassification, all Cry toxins were placed into a single group containing three domains [12]; however, the original principles with four ranks based on amino acid sequence identity were retained [13]. Despite the immense diversity, a three-domain organization of Cry proteins is conserved [4]. The α-helix-built domain I ensures pore formation [14], whereas β-sheets domains II and III are viewed as specificity determinants [9, 11, 15, 16].

Several trends in *Bt* evolution were described. Apart from mutationing under positive selection pressure [17], swapping of the third domain has been suggested as the primary evolutionary route allowing *Bt* to infect new hosts [18]. The recombination-driven domain exchange was proposed shortly after the sequencing of the first *cry* gene [19, 20], with increasing evidence being accumulated each year [21, 22]. The role of domain III swaps in expanding the natural diversity of Cry toxins was based on comparisons of the topologies of phylogenies corresponding to individual domains [18, 23]. The hypothesis was supported by *in vitro* experiments with domain III exchange to enhance toxicity [24] and by confirming the anti-coleopteran properties of Cry1B and Cry1I predicted by their phylogenetic grouping [25]. The domain-shuffling technique became a powerful tool for enhancing toxicity [26–28].

The discovery of domain swapping has broadened our knowledge of how Cry toxins evolve; however, we are still far from understanding what causes the exchanges. Multiple reports suggest that hybrids represent non-functional moieties [29]. In addition, the impact of domain swapping ranges from improving toxicity to impairing it [30–33]. To date, the exact molecular mechanisms controlling exchanges remain unknown, because the existing conceptions are based predominantly on laboratory experiments [24, 33–35]. Homologous recombination (HR) was proposed based strictly on genetic engineering experiments, which were not confirmed in environmental populations, because the molecular techniques utilized “tandem” plasmids, polymerase chain reaction (PCR)-based deoxyribonucleic acid (DNA) shuffling, or hybrid cloning [28].

At present, there are no large-scale studies of Cry toxins, given the abundance of available sequences. In this work, we applied sequence-based, phylogenetic, evolutionary, pangenomic, and functional bioinformatic analyses to fill the gap in our understanding of key drivers and consequences of domain swappings in Cry toxins by answering three fundamental questions to reveal (i) how prevalent the domain exchanges are and how significant their role is in forming novel toxins; (ii) which molecular mechanisms modulate the domain swapping process; and (iii) how these exchanges alter the host specificity of toxins and bacterial isolates. Here, we discovered the fundamental mechanisms controlling the evolution of Cry toxins, demonstrating that the rapid formation of diverse chimeric toxins implies site-specific recombination, which, in general, leads to the specialization but not broadening of the host range through negative selection of efficient hybrids.

## Materials and methods

### Data acquisition and processing

A detailed description of the methods is presented in Supplementary Texts S1.1–S1.10. The reproducible code and the raw data are available at the Git repository https://github.com/lab7arriam/Cry_phylogeny. The sequence dataset was obtained from the NCBI databases and Bacterial Pesticidal Protein Resource Center (BPPRC) [12]. Cry toxins were extracted with CryProcessor [36] and clustered with CD-HIT v4.8.1-2019-0228 [37] (Supplementary Texts S1.1–S1.2).

### Phylogeny reconstruction

To reconstruct phylogenetic trees, we used MAFFT v7.429 [38] followed by trimming with trimAl v1.4.rev22 [39] (Supplementary Text S1.3). Phylogenetic trees were reconstructed with RAxML-NG v1.1.0 [40] based on ModelTest-NG v0.1.7-reported [41] evolutionary models [39]. The trees were compared with tqDist v1.0.2 [42] and RAxML-NG quality-checked using phangorn v2.5.5 [43] and CollessLike v2.0 packages [44].

### Detecting recombination events

We utilized the RDP4 v101 [45], fastGEAR [46], Gubbins v3.1.2 [47], and ClonalFrameML v1.12 [48] for predicting recombination events, which were filtered based on the length of the transferred region and the congruence between parents and children on domain-wise trees. Only nearly-full exchanges affecting >70% of the domain length and those in which children and domain-wise parents formed the compact phylogenetic clades were retained (Supplementary Texts S1.4–S1.5, Fig. S1a). The recombination graph was constructed with the networkX v2.4 library [49]. The Circos plot was built with the circlize v0.4.13 package [50].

### Predicting possible mechanisms of domain exchanges

Pangenomes of *Bt* assemblies were reconstructed using Panaroo v1.2.8 [51] with subsequent r/m estimation with ClonalFrameML. Insertion sequences (IS) were detected with ISEScan v1.7.2.3 [52], prophages—Phigaro v2.3.0 [53], and genetic islands (GI)—IslandPath-DIMOB v1.0.6 [54]. Conserved blocks were extracted from 100 bp regions flanking breakpoints in parents. The pairwise identity distributions were obtained with the zoo v1.8-9 [55] package and further clustered (Supplementary Text S1.6). The conserved regions were obtained as the longest conserved k-mer chains (Supplementary Texts S1.7–S1.8) and clustered using the NbClust v3.0.1 package [56]. The consensus models were built with GLAM2SCAN v5.5.7 [57]. The identity between flanks was compared with the effects v.4.2-2 package [58]. Pangenome-wise associations were found using Scoary2 v0.0.15 [59].

### Studying the impact of recombination on host specificity

The host specificity spectrum was obtained from reviews [60–62], the BPPRC specificity database [12], research articles, and patents. To process the imbalanced data, we applied similarity metrics. The sets of hosts and the composition of toxins were compared using Szymkiewicz–Simpson and Jaccard coefficients (Supplementary Text S1.9). The host specificity-wise graph was constructed utilizing networkX v2.4 [49] with nodes representing strains and edges corresponding to toxins.

### Analyzing evolutionary selection

We applied a multi-step pipeline to prepare the data for assessing evolutionary selection in recombinants and parents. In brief, we took the sequences of parents and children, analyzing each domain independently with the respective subtrees from domain-based phylogenies, and marked parents and recombinants for each event (Supplementary Text S2.10, Fig. S1b). The evolutionary selection was calculated using the ETE3 v3.1.2 toolkit [63]. Site, branch, and branch-site models were analyzed (Supplementary Text S1.10, Fig. S1c).

## Results

### Phylogenetic and sequence-based inferences assume the independent evolution of the domains

To scrutinize the origin of Cry toxins, we prepared a comprehensive dataset of their natural diversity, leveraging four sources, namely, NCBI Assembly, GenBank, Identical Protein Groups (IPG), and the BPPRC [12]. A total of 3305 sequences of Cry toxins were retrieved (Fig. 1a), 941 of which remained after deduplication with a 100% identity threshold. To eliminate data redundancy, we selected 368 reference clusters using a 95% identity cut-off (Fig. 1b and c, Table S1). The domain-wise distribution of sequence lengths/identity was uniform and finely balanced (Supplementary Text S2.1, Fig. S2a–d, Tables S2 and S3). Most of the reference clusters were singletons (298); however, 7 clusters included over 10 representatives with altered allele flow in the third domain (Supplementary Text S2.1, Fig. S3a–g, Tables S1–S5). In reference sequences, the mean identity with the closest homologs from the database was lower compared to the redundant data (64% on average). It also included unreported holotypes from non-*Bt* species, such as *Clostridium botulinum* (Supplementary Text S2.2, Fig. S4a and b, Table S6).

**Figure 1:**
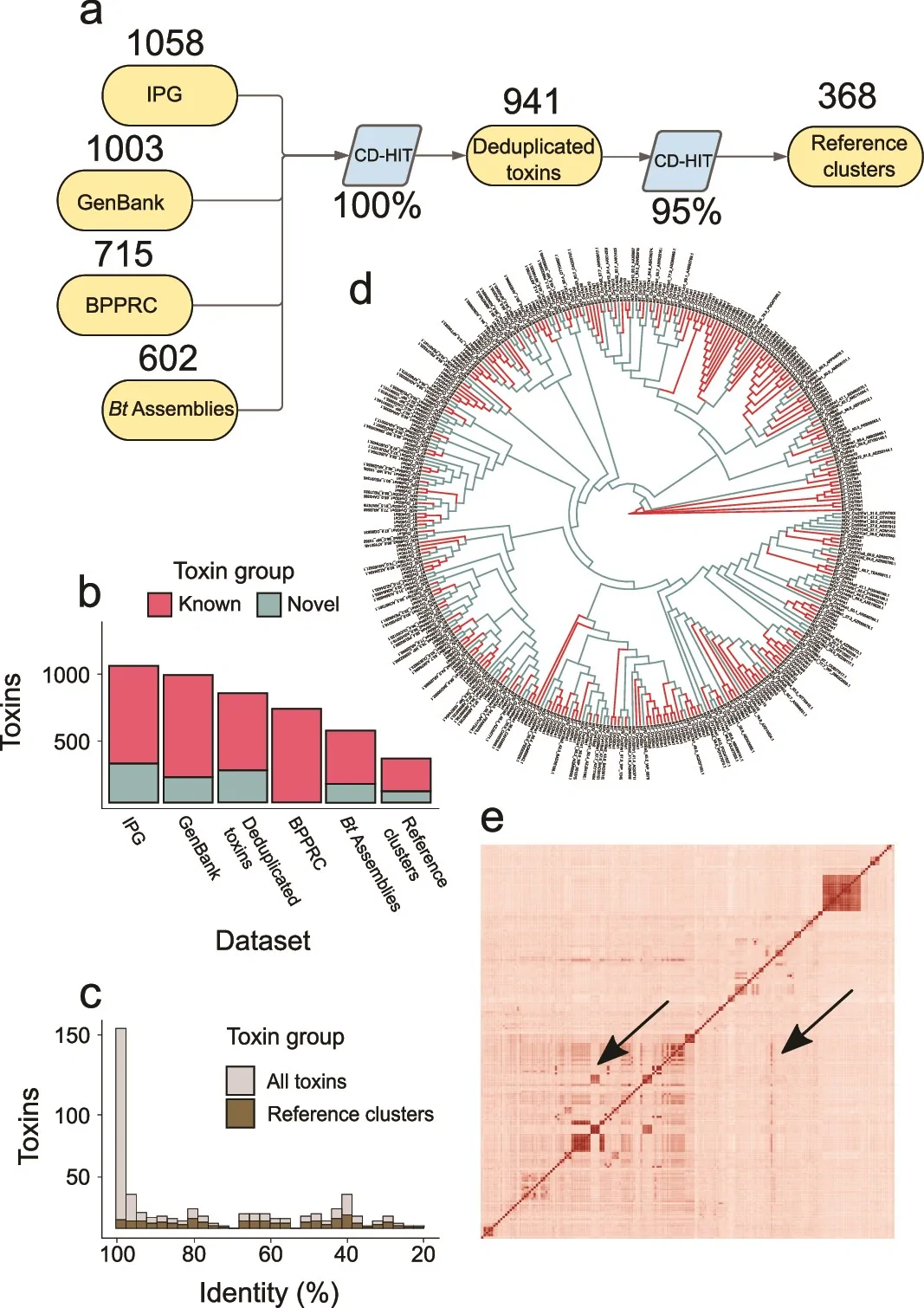
The properties of the cry toxins dataset and reference phylogeny. (a) The summary of Cry toxin amounts extracted from four datasets, namely, NCBI Assembly, GenBank, IPG, and BPPRC databases. (b) The distribution of Cry toxin types in the analyzed datasets. The color code denotes the type of toxins, with known toxins deposited in BPPRC and novel proteins designating putatively toxins with yet unstudied activity, yet belonging to the same protein class in terms of the domain composition (Supplementary Text S1.1). (c) The identity of the novel toxins with the closest homolog in BPPRC. The color code depicts the source of the dataset. Plotted are all unique toxins and reference clusters obtained using CD-HIT with a 95% identity threshold. (d) Reference ML-phylogeny (maximum likelihood) based on the concatenated alignments of the domains with partitioned evolutionary models. (e) The excised part of the heatmap with the pairwise identity of sequences of the third domain. The order of rows and columns follows the above-described phylogeny. Arrows point to the areas in which local identity does not comply with the expected order.

To reveal phylogenetic evidence of recombination, we reconstructed phylogenies from the sequences of the individual domains and a reference concatenated alignment (Fig. 1d, Fig. S5a–c). We compared the trees topologically and found that the tree of the domain III sequences varied sufficiently from the others (Fig. S5d–f, Table S7). Moderate dissimilarity was reported for the phylogeny of the first domain, whereas the second domain controlled the shape of the reference tree (Fig. S5e). The obtained incongruity was confirmed by the domain-wise identity distributions arranged by the reference tree with noticeable local identity peaks outside the diagonal (Fig. 1e, Fig. S6a–c, Table S8). Such an effect was likely caused by recombination, especially vivid in domain III and, to a certain point, domain I (Supplementary Text S2.3, Fig. S6a–c). We found that all the trees exhibited a high homoplasy rate, with a consistency index (CI) of <0.1, and were also unequally balanced (Table S9). Given that CI is a typical phylogenetic hallmark of recombination [64], we confirmed that recombination played a key role in maintaining the diversity of the toxins.

To reveal explicit evidence of recombination, we utilized RDP4 and revealed 120 events accordingly (Table S10). Toxins were classified as recombinants or minor and major parents, which transferred only one domain or two domains, respectively. The number of exchanges dropped to the 50 most probable events after applying filters based on phylogenetic congruence (Supplementary Text S2.4, Figs S7a–S10b, Tables S11–S16). The tree-based analysis of the unknown parents (see Supplementary Text S1.5) also showed that there are at least 40 putative Cry types yet to be discovered (Table S17). There were more exchanges of the third (33) and first (13) domains (Table 1), and only four events of the second domain were detected (Fig. 2a and b, Fig. S7a–f). Forasmuch as we analyzed domain-wise phylogenies independently, we observed 14 events with a long trail of single-domain parents, particularly of domain III, which are neither majors nor minors (Fig. S10b). We proposed a permanent flow of domain swapping, which was confirmed by the parent-wise mismatch distributions (Supplementary Text S2.5, Figs S10b–S11c, Tables S18–S21).

**Table 1:** Recombination events engaging the first domain. Presented are chimeric toxins and their parents, domain-wise identities, and toxicity spectrum. Recombination IDs correspond to the Supplementary material. More detailed information on general properties and activities is present in Tables S12 and S49–50. Novel toxins are coded according to the following scheme: NOV_ < *the closest known reference* > _ < *identity with reference* > _ < *accession number*>. Similarity metrics between recombinants and parents are given as two estimates representing identities between domain I and domains II/II jointly. The data on the toxicity spectrum were taken from articles and parents and is available in Table S45. Toxicity is reported only if the data for recombinants is known.

ID	Recombinants	Minor parents	Major parents	Identity, % (D-I/D-II + III), major	Identity, % (D-I/D-II + III), minor	Hosts (recombinants)	Hosts (minor parents)	Hosts (major parents)
34	NOV_Cry14Ab1_42.4_PGW46196.1	NOV_Cry14Aa1_44.0_PGW42274.1	NOV_Cry14Aa1_41.1_PGW46198.1	77.7/56.1	55.7/85.5	**Coleoptera** (*Diabrotica virgifera virgifera*); **Hemiptera** (*Nezara viridula*)		**Hemiptera** (*N. viridula*)
38	Cry59Ba1	Cry40Ca1; Cry40Da1; Cry40Ba1; NOV_Cry40Da1_81.5_AIN76756.1; NOV_Cry40Da1_77.3_ARJ25823.1; NOV_Cry40Da1_65.0_CAH33957.1; NOV_Cry40Da1_66.9_PEU78339.1	Cry59Aa1	70.9/51.9	54/85.7	**Diptera** (*Aedes aegypti*); **Lepidoptera** (*Spodoptera exigua*)	**Diptera** (*Culex quinquefasciatus*)	
45	NOV_Cry40Da1_65.0_CAH33957.1	NOV_Cry40Da1_66.9_PEU78339.1	unknown	95.3/67.7				
47	Cry2Ai1	Cry2Ac10	Cry2Aa13	97.6/74.5	81.2/98.7			
56	Cry9Ca1	unknown	Cry9Ed1; Cry9Ec1		58.9/68.2	**Lepidoptera** (*Agrotis ipsilon*; *Agrotis segetum*; *Bombyx mori*; *Cacyreus marshalli*; *Chilo suppressalis*; *Choristoneura fumiferana*; *Danaus plexippus*; *Epinotia aporema*; *Helicoverpa armigera*; *Heliothis virescens*; *Lobesia botrana*; *Manduca sexta*; *Ostrinia nubilalis*; *Pectinophora gossypiella*; *Plutella xylostella*; *S. exigua*; *Spodoptera littoralis*; *Trichoplusia ni*)		**Lepidoptera** (*B. mori*; *O. nubilalis*; *P*. *xylostella*)
62	Cry1Db1	NOV_Cry1Da1_83.2_AIW52615.1	Cry1Da1	88.4/70.3	74.0/94.1	**Lepidoptera** (*B. mori*; *Epiphyas postvittana*; *Spodoptera litura*)	**Lepidoptera** (*H. armigera*; *P*. *xylostella*)	**Lepidoptera** (*Anticarsia gemmatalis*; *B. mori*; *C. marshalli*; *C. fumiferana*; *Conopomorpha cramerella*; *Cydia pomonella*; *Earias insulana*; *H. armigera*; *Lambdina fiscellaria*; *Lymantria dispar*; *Malacosoma disstria*; *M. sexta*; *Orgyia leucostigma*; *P*. *gossypiella*; *Sesamia inferens*; *S. exigua*; *Spodoptera frugiperda*; *S. littoralis*; *S. litura*)
75	NOV_Cry32Ta1_48.0_AQX37180.1	Cry32Qa1	Cry32Va1; NOV_Cry32Va1_61.0_WP_134996253.1	68.9/53.7	54.6/55.4			
78	NOV_Cry56Aa2_56.0_AGV55019.1	NOV_Cry56Aa4_56.4_PEU80288.1	unknown	77.4/65.1				
84	NOV_Cry8Aa1_47.1_EEM92570.1	Cry8Ea2	NOV_Cry8Da1_43.7_ANC11094.1	68.4/51.2	56.2/64.1			
87	Cry1Be1; Cry1Bj1	Cry1Bf1; Cry1Ba1	Cry1Bb1	89.0/65.7	72.7/70.0	**Lepidoptera** (*A. gemmatalis*; *Chrysodeixis includens*; *Helicoverpa zea*; *H. virescens*; *O. nubilalis*; *P*. *xylostella*; *S. exigua*; *S. frugiperda*; *T. ni*)	**Coleoptera** (*Acanthoscelides obtectus*; *Anthonomus grandis*; *Chrysomela scripta*; *Hypothenemus hampei*; *Leptinotarsa decemlineata*; *Xylotrechus arvicola*); **Diptera** (*Lucilia cuprina*; *Lucilia sericata*; *Musca domestica*); **Hemiptera** (*Diaphorina citri*); **Lepidoptera** (*A. gemmatalis*; *C. marshalli*; *C*. *suppressalis*; *C. fumiferana*; *C. includens*; *C*. *cramerella*; *C*. *pomonella*; *Diatraea grandiosella*; *Diatraea saccharalis*; *E. insulana*; *Eritingis aporema*; *E*. *postvittana*; *H. virescens*; *M. disstria*; *M. sexta*; *Ostrinia furnacalis*; *O. nubilalis*; *Perileucoptera coffeella*; *Phthorimaea operculella*; *Pieris brassicae*; *Pieris rapae*; *P*. *xylostella*; *S*. *inferens*; *Sesamia nonagrioides*; *S. frugiperda*; *Thaumetopoea pityocampa*; *T. ni*; *Wiseana cervinata*; *Wiseana copularis*; *Wiseana jocosa*)	**Lepidoptera** (*C. includens*; *L. dispar*; *M. sexta*; *O. nubilalis*; *P*. *xylostella*; *S. exigua*; *S. frugiperda*; *T. ni*)
95	Cry2Am1	Cry2Al1	Cry2Ag1; Cry2Ab15	96.1/82.1	84.8/93.5	**Diptera** (*A. aegypti*)	**Lepidoptera** (*H. armigera*; *S. litura*)	**Diptera** (*A. aegypti*; *Anopheles gambiae*; *Rhagoletis cerasi*); **Lepidoptera** (*C*. *suppressalis*; *C*. *pomonella*; *D*. *saccharalis*; *E. insulana*; *H. armigera*; *Helicoverpa punctigera*; *H. zea*; *H. virescens*; *Leguminivora glycinivorella*; *L*. *botrana*; *L. dispar*; *Malacosoma neustria*; *M. sexta*; *Mythimna separata*; *O. nubilalis*; *P*. *gossypiella*; *P. brassicae*; *P. rapae*; *P*. *xylostella*; *S. exigua*; *S. frugiperda*; *T. ni*)
96	NOV_Cry19Ba1_74.0_AGV55021.1	Cry19Ba1	Cry19Ca1	89.3/62.0	68.4/68.7			
119	Cry14Ab1	NOV_Cry14Ab1_85.6_AXE15628.1	Cry14Aa1	92.6/81.5	79.7/86.6	**Nematoda** (*C. elegans*; *Heterodera glycines*)		**Nematoda** (*Bursaphelenchus xylophilus*; *C. elegans*; *Distolabrellus veechi*; *Nippostrongylus brasiliensis*; *Panagrellus redivivus*)

Insect order names are shown in bold and Insect species names are italized.

**Figure 2:**
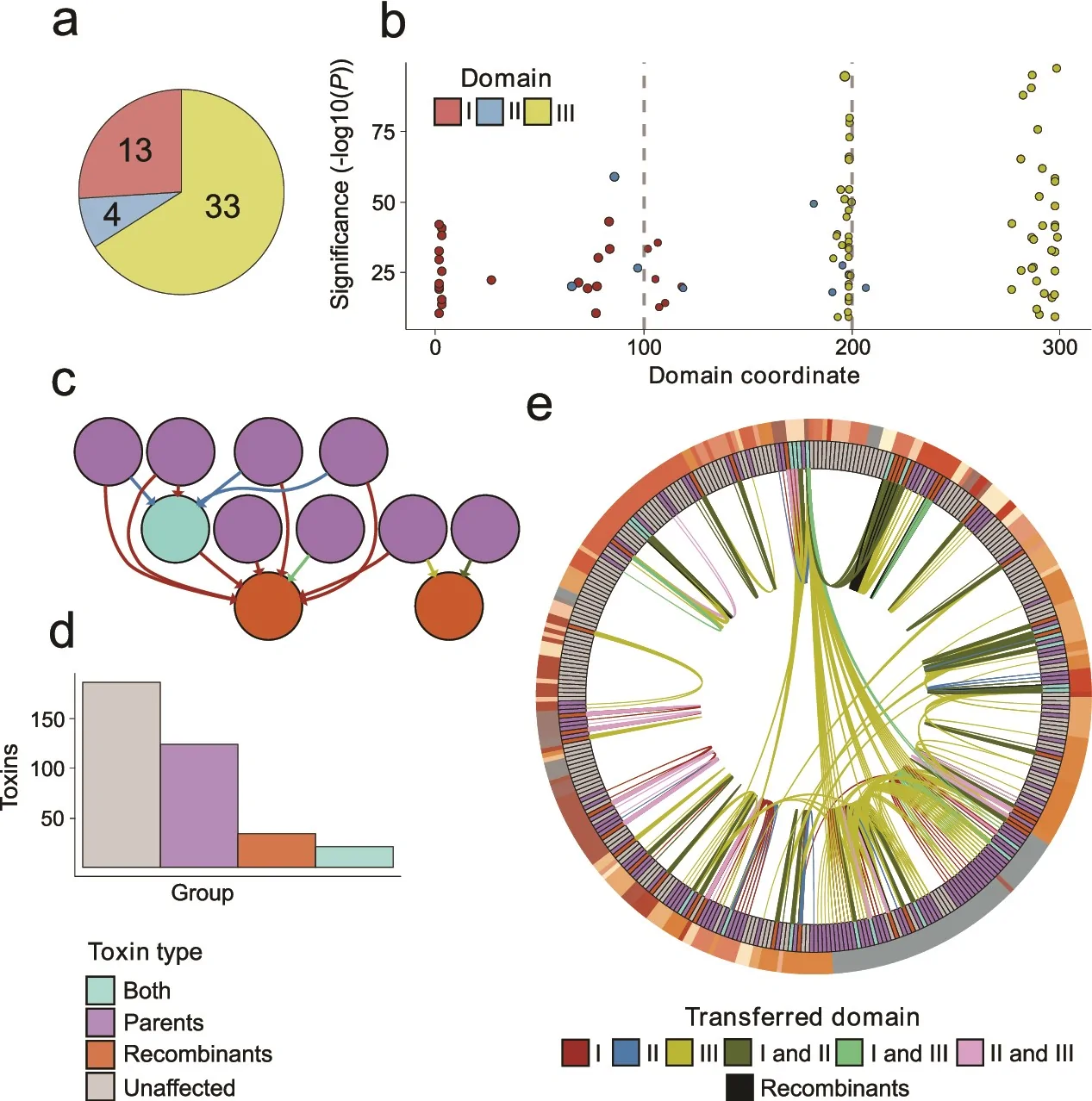
The recombination landscape of Cry toxins. (a) The total number of recombination events identified by the RDP4 utility and classified according to swapped domains. Shown are data for the filtered dataset, i.e. including events with >70% of the respective domain subjected and forming compact clades within domain-wise phylogenetic trees. For a detailed description of the filtration process, consult Supplementary Text S1.4. (b) Distribution of recombination breakpoints. On the *x*-axis, the relative coordinate in the sequence is presented. Plotted on the *y*-axis is the mean *P*-value from all recombination detection tests implemented in the RPD4 software. Gray dotted lines mark the boundaries of the domains. (c) An example of one weakly connected component extracted from the graph of domain exchanges (Fig. S12). The color of the node displays the role of the toxin in recombination events (recombinants, parents, and those participating in multiple swaps being parents and recombinants simultaneously). Edge colors denote the type domain(s) transferred, namely, a single domain for minor parents and two domains for major parents, respectively. (d) The number of toxins according to their type in recombination events. (e) Circos plot illustrating the map of domain exchanges with sectors corresponding to the order of the reference phylogeny. The color of the inner circle represents the toxin types in recombination events. Links are colorized by the domain transferred. Black curves connect toxins if a particular event contains more than one recombinant. The inner circle marks the first-rank Cry toxin class according to the established Cry nomenclature.

To draw a map of domain exchanges, we reconstructed a graph with nodes representing toxins and edges corresponding to the transferred domains (Fig. 2c, Fig. S12). The graph consisted of 310 edges, 180 nodes, and 22 connected components. The most abundant connected component encompassed 19 events, whereas 16 were singletons (Fig. S13a–c, Table S22). We categorized toxins as parents, recombinants, and both donors and recipients of the domains (Fig. 2d). These classes comprised 125, 34, and 21 toxins, respectively (Table S23). Thus, 48.9% of the unique reference toxins in the known diversity took part in recombination. The edges from the graph mapped on the reference tree were characterized by multiple links of variable phylogenetic depth, indicating both the intensity of the exchanges and their influence on phylogeny (Fig. 2e). There were three pairs of events in which the same toxin was spotted, reflecting the continuous nature of domain shifting (Supplementary Text S2.6, Fig. S14, Table S24).

The network analysis showed that domain exchanges occurred multiple times during the evolutionary history of Cry toxins, forming novel proteins with altered domain combinations. Toxins tend to constitute isolated recombination groups of within-group exchanges or, conversely, abundant connected components, implicating possible specialization from a larger pool of ancestral forms. Identification of the driving force controlling transfer events requires a detailed study of the genomic context surrounding *cry* genes.

### Domain exchanges are driven by flanking insertions and inter-domain conserved regions

To uncover swapping mechanisms, we compared the HR rate in *Bt* genomes that bore toxic loci or were devoid of them. Using a pangenomic approach, we found that the recombination-to-mutation rate (R/θ) was unexpectedly lower in recombination-affected genomes (Supplementary Text S2.7, Fig. S15a–f, Tables S25–S26). Nevertheless, the pangenomes were open, implying high genetic plasticity (Fig. S15a–c). We thus proposed that HR per se does not explain these domain exchanges and that alternative mechanisms exist. To this end, we searched for inclusions of *cry* loci into mobile genetic elements (MGEs). The assemblies containing *cry* loci carried significantly (*P* < 1e-08) more MGEs, especially IS (Supplementary Text S2.7, Fig. S16a–d, Tables S27–S28), and 36% of genes affected by domain swapping were included in MGEs (Fig. S16e). Multiple flanking insertions surrounding *cry* genes were identified (Fig. 3a).

**Figure 3:**
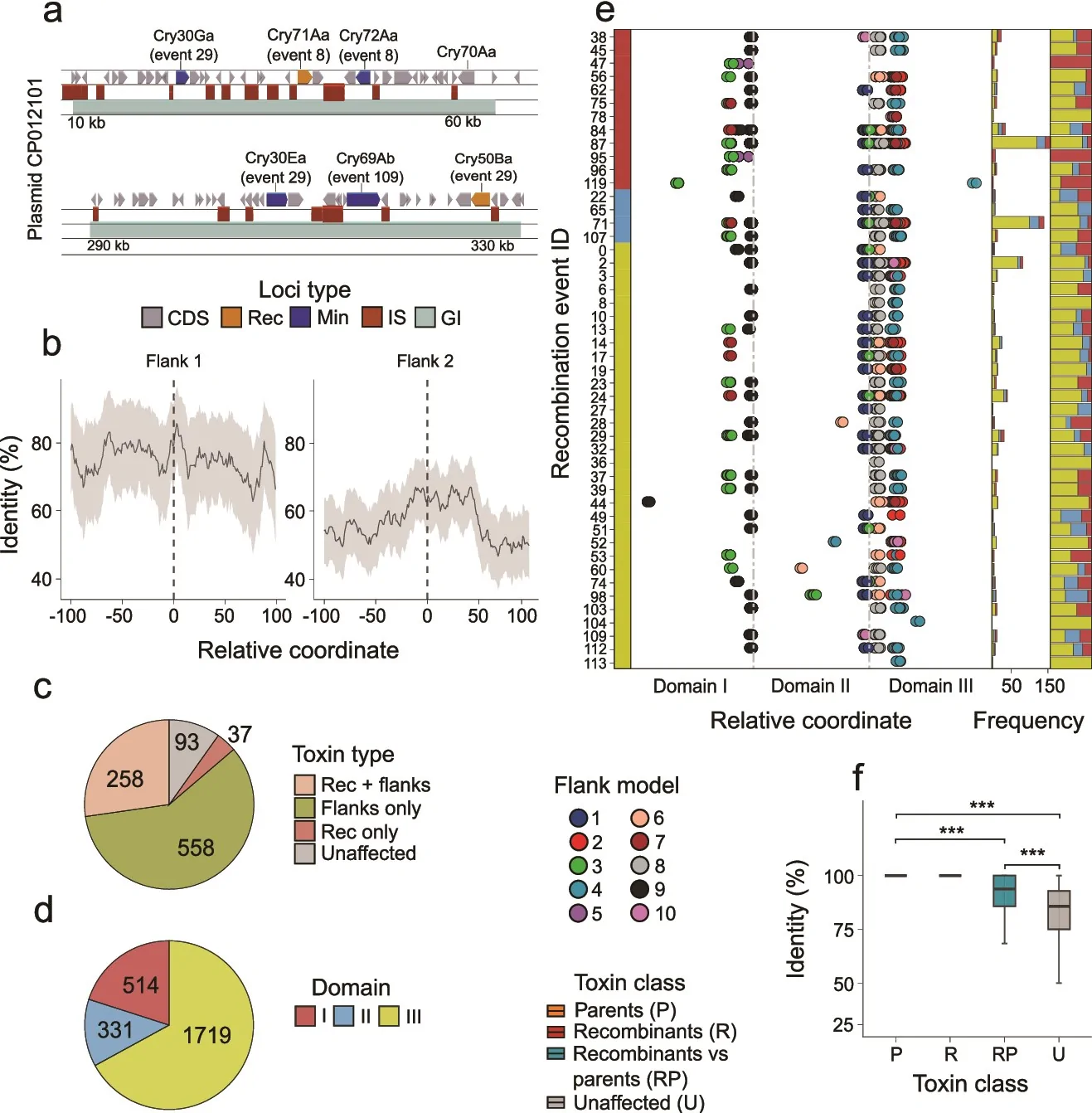
The mechanisms of domain exchanges driven by mobile genetic elements and domain-wise identity within breakpoints. (a) Loci surrounding genes encoding recombinants and parents from individual recombination events in the strain HS18-1. The color of the blocks on the first track corresponds to the role of toxins in recombination events, namely, major and minor parents, recombinants, and unaffected toxins. (b) Coordinate-wise mean identity between parental sequences in recombination events within two regions located between the first and the second domains (“Flank 1”) and between the second and the third domains (“Flank 2”). The identity is calculated for 100 bp flanking regions before and after the breakpoints. The dashed lines point to the breakpoints. Gray areas represent the standard error. (c) The overall composition of toxins regarding their role in recombination and/or the presence of signals from consensus models based on conserved flank clusters. (d) The classification of signals from flank clusters relative to the domain coordinates in the respective toxins, with the hits accordingly. (e) The distribution of hits with flank-based models in the participants of domain swapping events. Plotted on the *X*-axis are the relative coordinates normalized by the domain lengths. The *Y*-axis represents the unique identifiers of recombination events classified according to the domain obtained from the minor parent, which is specified in the leftmost strip. The points are colorized according to the flank model, matching the regions from the parental and recombinant toxins. The two adjacent plots on the right summarize the number of hits relative to the domain in which the consensus regions similar to flank models are located. On these plots, the total number and the proportion are shown, respectively. (f) Comparison of the pairwise identity estimates within flank regions irrespective of the cluster between toxin groups attributed to their role in domain exchanges, such as recombinants (“R”), parents (“P”), children compared to the parents (“RP”), and other toxin pairs, unaffected or not from the same events (“U”). The angular bracket points out that the depicted significant differences correspond to both parents and recombinants relative to other toxin groups. Asterisks denote significant differences according to the Wilcox test with *P* values adjusted using the Benjamini–Hochberg procedure.

The HR rate in *Bt* strains is low, implying that domain swapping is driven by a molecular mechanism alternative to HR. We hypothesized that domain exchanges are linked with regions of increased similarity between parents. To find recombination points, we extracted conserved blocks within the boundaries of the breakpoints (Supplementary Text S2.8, Table S29). We found that breakpoints between domains I/II and domains II/III were surrounded by peaks flanked by two local minima (Fig. 3b). The event-wise clustering revealed that similarity patterns of clustered events gained offset peaks with higher (*P* < .05) identity relative to transferred and adjacent domains (Supplementary Text S2.8, Figs S17a–S18c, Tables S30–S32).

Because domain transfer typically affects the whole sequence unit, we suggested that certain families of conserved blocks exist in *cry* sequences. To unveil the diversity of blocks, we leveraged the k-mer distributions and extracted 37 individual flanks forming 10 consensus models (Supplementary Text S2.9, Figs S19a–S20d, Tables S33–S41). We used these models to search for the signals in the deduplicated set of all Cry sequences. A total of 2592 signals in 816 toxins were found, 558 of which participated in recombination events (Fig. 3c, Table S42). The site-wise distribution indicated the presence of five key zones with conserved regions, two of which concentrated between domains I and II, and three between domains II and III (Fig. S21a–c). Flank locations in the domains resembled recombination events, given that 79% of flank models aligned to the third domain (Fig. 3d, Fig. S22a). The vast majority of parents and recombinants possessed homologous regions of flanks in the first and the third domain jointly (Fig. 3e), corresponding to the prevalence of these types of exchanges.

To reveal if and how specific flank sequences are related to recombination events, we compared sequence identities and applied pangenomic analysis. The identity-wise comparisons illustrate that representatives of the same events exhibit higher identity (*P* < 10e-16) in the flank regions compared with other toxins (Fig. 2f, Supplementary Text S2.10, Fig. S22b–e, Table S43). Thus, domain exchanges coincide with local similarities in the boundaries between the domains in parental sequences. To identify the probable molecular machinery, we detected pangenome-wide associations with distinct flank clusters in *cry* loci. A total of 782 pangenome clusters for 9 flank types were detected (Table S44). Apart from the most frequent group of clusters with an unknown function, we revealed the following functional groups: (i) transcription regulators, (ii) transporters, (iii) phage and transposon-related components, and (iv) enzymes acting on molecular processes affecting DNA, including biosynthesis, repair, replication, and recombination. In the latter group, we found an association (*P* < 1e-11) with tyrosine recombinases/integrases of the XerCD family (Table S44). For a total of 7 flank models, four associations were found. One pangenomic cluster was shared by all flank groups, which fits with the presence of multiple signals in *cry* sequences (Fig. S21a and b). Three other clusters were associated with several flank types, engaging 1–3 representatives.

Distinct features of *cry* loci suggest that domain shuffling is maintained by conserved regions between the domains. Conserved flanks of diverse clusters are associated with XerCD recombinases that manage the resolution of circular chromosomes and plasmids. These proteins are highly prevalent among bacteria [65] and act by recognizing small 28-bp conserved sites. We propose that homologous intra-domain flanks (Fig. S21a and b) coupled with IS surrounding *cry* genes (Fig. 3a) serve as targets for XerCD recombinases, sparking the domain swapping. The widespread machinery orchestrating the formation of chimeras raises a question of how multiple recombination events affect the host specificity spectrum of toxins and strains.

### Domain swapping modifies the host specificity of chimeric toxins and alters the range of produced toxins in the affected strains

To characterize the effect of domain exchanges on host range, we analyzed the toxins and strains harboring them. The metadata for Cry proteins and strains were taken from BPPRC [12], reviews [60–62], and other literature sources, excluding better-studied toxins when assessing the number of affected species to adjust the patchy data (Supplementary Text S2.11, Fig. S23a–d, Tables S45–S47). We studied host orders and revealed that parents were generally different from children based on the host range. Major parents were more similar to recombinants in 65% of cases (Fig. 4a, Supplementary Text S2.12, Figs S24a–S26b, Tables S48–S51). Similarly, the species range was generally unequal; however, major parents contributed more to the activities of recombinants on the species level (Supplementary Texts S2.12–S2.13, Figs S26a–S29c, Tables S51–S58). Recombination did not cause traceable quantitative changes in host spectra but altered the composition of the affected species (Supplementary Text S2.14, Figs S30a–S31c, Tables S59–S62).

**Figure 4:**
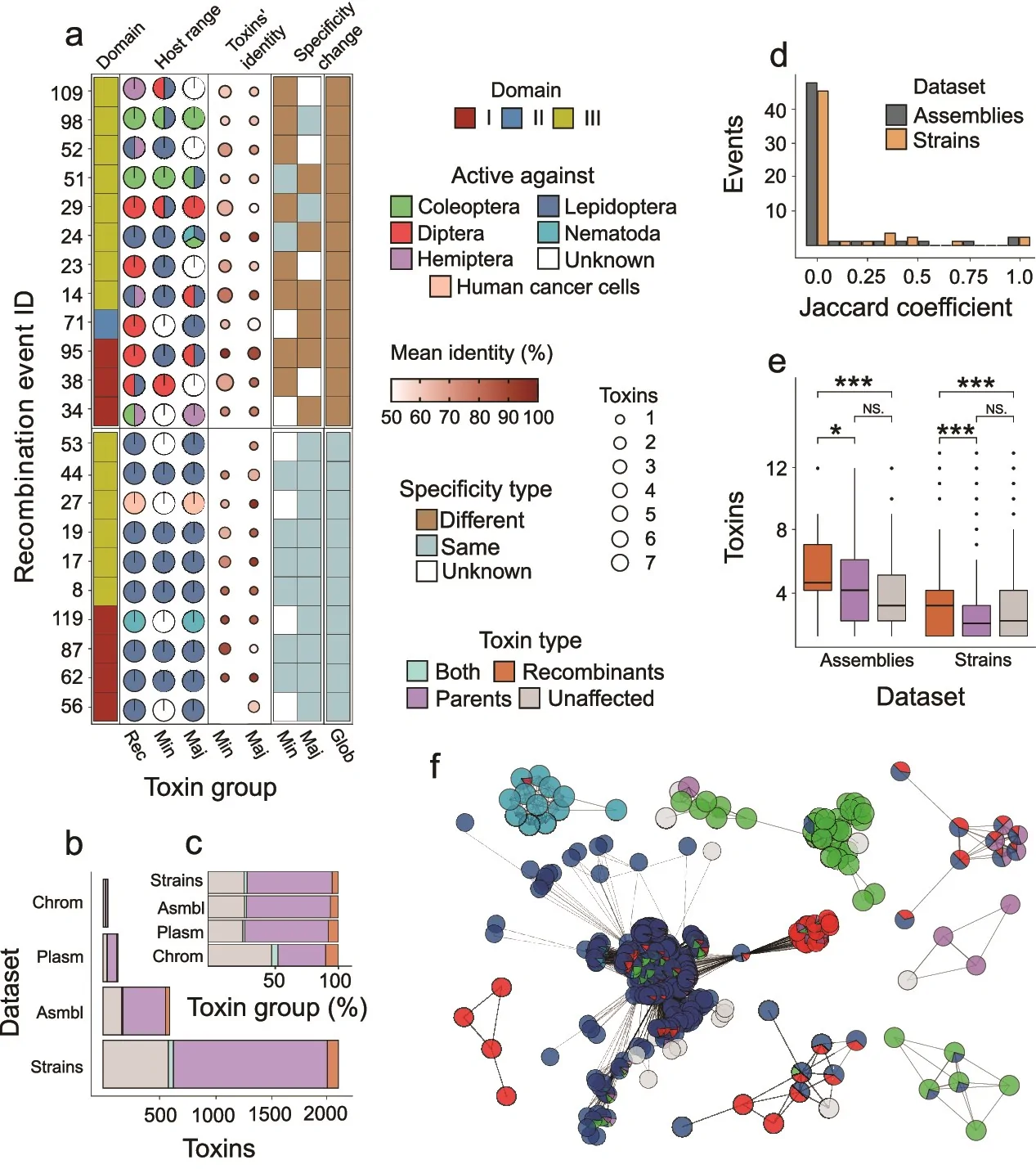
The impact of recombination on host specificity of Cry toxins and the toxin content in bacterial strains. (a) The percentage of orders against which recombinants and parents are active. The data on the toxicity spectrum are based on toxins only, without additional information for the strain. The toxicity spectra were processed using the ancestral reconstruction procedure. The portion of host orders within the pie charts is color-coded. The events are split into two panels, namely those in which at least one toxin exhibits activity against host orders differing from other types of parent and/or child. The left adjacent bar is designated for the domains transferred in the events. The right adjacent panel shows the number of toxins. “Rec” indicates recombinants, “Min”—minor, and “Maj”—major parents, respectively. The size of the dots relies on the number of participants, whereas the intensity of the color delineates the mean pairwise identity between the transferred domains. (b) The number of particular toxin types in the datasets, such as strains, assemblies, plasmids, and chromosomes containing recombinants, parents, and unaffected toxins, and the fraction (c) of these toxin groups. (d) The similarity between all sets of toxins that are housed by strains and assemblies in which at least one parent and recombinant reside determined using the Jaccard coefficient (the length of the intersection divided by the union of the sets). Event-wise comparisons refer to toxin compositions in the assemblies and strains attributed to recombinants and all parents of the recombination events. (e) The number of toxins comprised in the assemblies and strains bearing recombinants, parents, and cry loci devoid of recombination signals. Asterisks denote significant differences according to the Wilcox test with *P* values adjusted using the Benjamini–Hochberg procedure. (f) Selected connected components from the weighted specificity-wise graph of strains sharing the common cry toxins. The nodes represent strains, whereas edges are drawn if at least one toxin within the third rank is found in two connected strains. The weight of the edges is determined by the number of shared toxins. The color of the nodes visualizes the overall proportion of species attributed to the orders of the toxins produced by the strain that exerts the effect on.

To consider specificity changes in the context of ancestral exchanges, we applied a soft approach to calculate the similarity by at least one shared activity between toxins. In this mode, affected orders were common in most instances, especially when comparing minor and major parents with each other (Fig. S32a–d, Tables S63–S65). Regarding species, a higher percentage (85% vs. 38%) of child pairs with major and minor parents exhibited shared hosts (Supplementary Text S2.15, Fig. S32c–f, Tables S64 and S66), demonstrating the formation of the hybrid toxins from major parents in the same ecological niche. Although no significant differences were found when analyzing toxins per se due to scarce data, in 86.4%–93.5% of events, considering all parents and descendants, the number of hosts of recombinants either remained the same or was lower than in the respective parents (Table S67).

To ascertain whether site-specific recombination alters the composition of toxins, providing additional benefits to host adaptation, we classified strains/genomes according to toxins they contain (Supplementary Text S2.16, Fig. S33a and b, Table S68). Unlike unique toxins, 48.9% were associated with swapping events (Fig. 2d); the fraction of recombination-affected *cry* genes in all known strains reached 73%, indicating that engaging in domain exchanges is widespread in nature (Fig. 4b and c). Participants in recombination co-occur. Therefore, if a strain contains at least one recombinant, it would probably include parents and/or recombinants from other events (Supplementary Text S2.17, Fig. S33c–h, Tables S68–S70). The co-occurrence of the affected loci from the same events in one genome was scarce. Of the six identified instances, the respective genes fell into MGEs and were flanked by IS (Supplementary Text S2.18, Fig. S34a–c, Table S71). The composition of produced Cry toxins in isolates bearing recombinants and parents from the same events was wholly different for 70% of the instances (Fig. 4d, Supplementary Text S2.19, Fig. S35a and b, Table S72).

To test the relationships between toxin composition and host range, we collected specificity data for toxins within natural strains. The total number of toxins in strains/assemblies housing recombinants was significantly (*P* < .05) higher compared to those containing only parents or unaffected by recombination events (Fig. 4e, Supplementary Text S2.19, Fig. S35c, Table S73). On average, more hosts (*P* < .05) were attributed to recombination-subjected assemblies and strains compared to unaffected, and the maximum estimates were found in strains bearing major parents but not children (Supplementary Text S2.20, Figs S36a–S38 h, Tables S74–S83). On a closer look, entities with major parents produced more toxins, which corresponds to the relationships between the host range and the abundance of toxins (Supplementary Text S2.21, Fig. S39a, Tables S82–S84). The event-wise examination of strains corroborated that the host range of children depends primarily on major parents, but the number of the affected orders in descendants is commonly lower (Supplementary Text S2.21, Figs S39b–S40c, Table S85).

Analysis of Cry-producing bacterial strains illustrated that parents and recombinants stem from the same ecological niche, and the host spectrum of recombinants is delineated mainly by major parents, but the species-wise specificity is most probably altered, coupled with the changes in the toxin content. A lower host range in recombinant strains relative to major parents supports specialization. However, the signals of specialization were implicit, and tracing the overall trends requires examining larger groups of bacterial isolates to reveal which events shape the global population landscape.

### Domain exchanges accompany the adaptation to hosts during the emergence of closed specialist populations

We detected vague traces of reduced host range in the isolates with chimeric toxins affected by recombination. To obtain a general picture, we scaled to a population level using a specificity-wise graph with nodes corresponding to strains and edges denoting shared toxins (Fig. 4f, Fig. S41). The graph was composed of 948 nodes forming 169 connected components, and the largest one included 556 strains with 73 recombination-affected toxins (Supplementary Text S2.22, Fig. S42a and b, Tables S86–S87). Toxin composition in components containing parents and children was unequal, irrespective of the parent type (Fig. S42c, Table S87). Overall, the connected components encompassing toxins subjected to recombination exhibited a higher number of hosts and toxins as well (Supplementary Text S2.22–S2.23, Figs S42d–S43a, Table S86).

Donors and acceptors of individual domains in swapping events fell into the connected components with distinct strains, namely, specialists and generalists, active against only one host order or multiple hosts, respectively (Supplementary Text S2.24, Figs S43b–S44b, Tables S86–S88). The whole components were classified as generalists if they contained >70% of generalist isolates (Table S89). We analyzed both individual isolates and connected components in terms of generalism and types of toxins attributed to them. In components, all groups of toxins according to recombination events co-occur (Fig. S45a–c, Table S90), whereas in strains only, we found a significant (*P* < .01) negative correlation between the number of majors and recombinants, as well as between majors and minors (Fig. S45d, Table S91). This indicates that parental strains are generally conserved and remain in the global population, whereas certain recombination-prone strains collect chimeric toxins.

The whole connected components were grouped in the specialist category, whereas individual strains (either included in them or isolated) were predominantly generalists (Figs S46a–S47b, Table S92). More specifically, the fraction of specialist strains with recombinants exceeded 50%, whereas >90% of the isolates producing major parents were generalists, corroborating the link between specialization and recombination (Fig. S47b). Unlike the significant positive correlation between the number of toxins and hosts based on the dataset of all isolates, generalists infected significantly more (*P* < .001) host orders, whereas specialists produced more toxins in general (Fig. S47c and d, Table S93). A seeming contradiction is explained by the fact that, in generalists bearing major parents, the more toxins are produced, the lower the host range is (Fig. S48a and b, Tables S94–S95), signifying that at certain time points, the bacterial subpopulations lose toxins with a broad host range and, conversely, gather multiple host-specific toxic moieties.

To reveal population-wise consequences of specialization, we introduced strategies by taking all strains and connected components encompassing toxins (i.e. major/minor parents and children in recombination events) and defined their event-wise types as generalists if >70% of components/strains were generalists and specialists otherwise (Fig. S49a–f, Table S96). Qualitatively, most of the events represented the exchanges between specialists (Fig. S50a). Quantitatively, bacterial isolates most frequently contained toxins engaged in the events leading to the occurrence of specialist recombinants from generalist major parents (Fig. S50a and b, Table S97), which was supported by the reduction in the number of hosts in the strains/components encompassing recombinants (Fig. S50c and d, Tables S98–S99). In bacterial strains/components with more frequent recombination events, the percentage of generalists is lower (Fig. S51a–c, Table S100).

Population-wise landscape suggests that recombination-guided domain swapping is significantly associated with specialization. Recombining bacterial populations with an extended host range eventually form local subpopulations that gather chimeric toxins active against the same host range and subsequently separate from the initial pool of generalists. Such a process assumes that receiving novel domains is evolutionarily advantageous, which should be proven by analyzing evolutionary selection signals.

### Domain swapping is accompanied by purifying selection

Once recombination occurs, the chimeric sequences could be subjected to evolutionary selection, both positive and negative, with the latter indicating that the changes become fixed. We analyzed whether domain exchanges become conserved by detecting selection signals (Supplementary Text S1.10). To identify the association between recombination and evolutionary selection, we focused on the branch model by marking either children or parents on the trees reconstructed from individual domain sequences. We detected 83 signals, and only 4 of them represented positive selection, whereas others were under purifying selection (Fig. 5a, Fig. S52a, Tables S101–S102). The foreground ω (the non-synonymous to synonymous substitution rate ratio, dN/dS) was 0.367, which corroborates pronounced selection (Fig. 5b, Supplementary Text S2.25, Fig. S52a–e). Similar to generally unequal mutation rates in the domain III sequences, there were prominent differences in selection-subjected domains in recombinant and parental sequences mirroring ancestral exchanges (Fig. 5a, Supplementary Text S1.26, Fig. S52a, Table S101).

**Figure 5:**
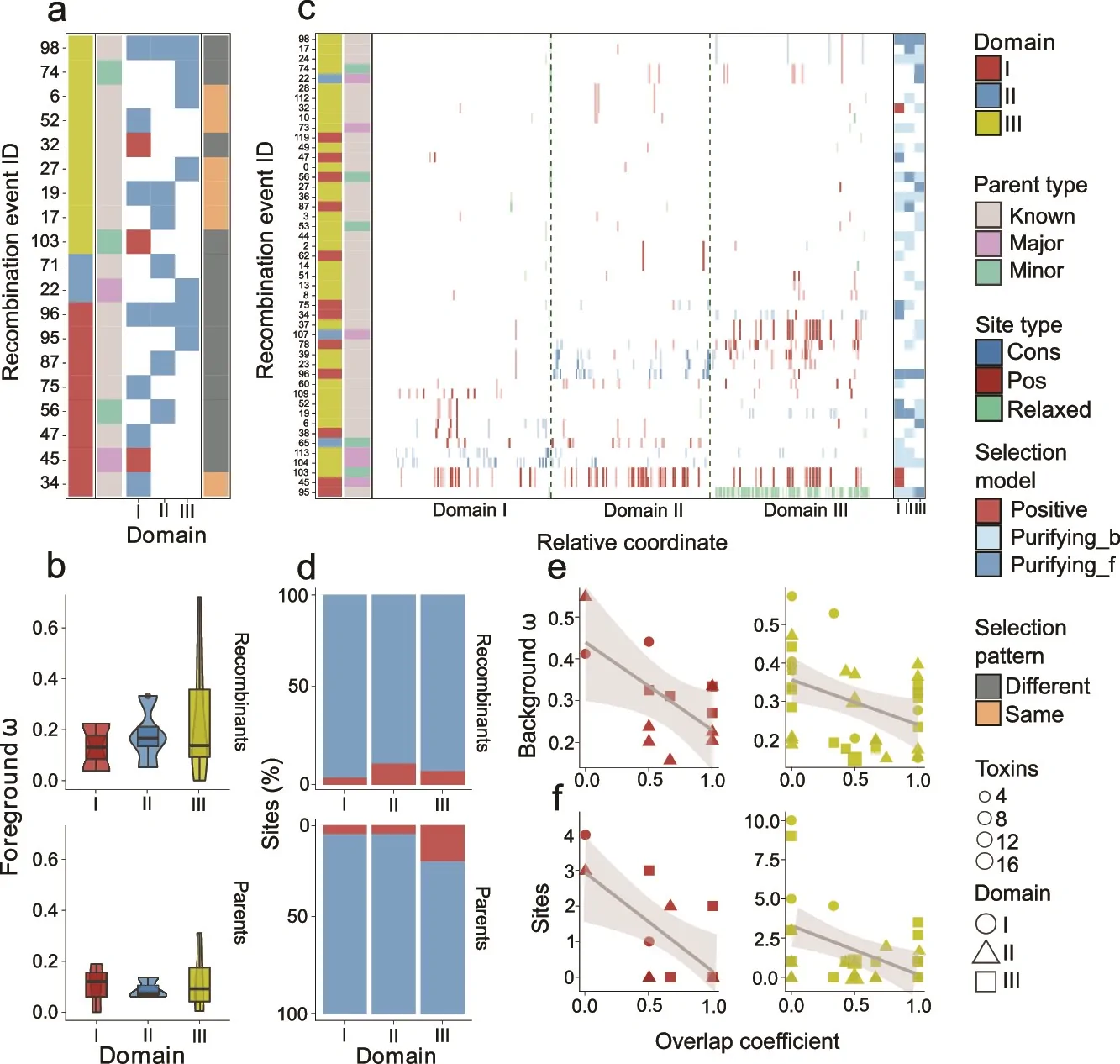
The relationships between domain exchanges and evolutionary selection signals in *cry* sequences. (a) The results of an evolutionary selection analysis calculated on the branches in which recombinants were marked. Here and in other figures, the selection pressure was determined on the sequences of different domains independently. The heatmap shows the models reported for the individual domains of recombinants. The color of the blocks indicates the presence of positive or purifying selection. The left adjacent subpanels correspond to the domain that was transferred from the minor parent and show whether a parent, either minor or major, is absent in the dataset. The right subpanel displays whether, for at least one domain, a different selection pattern from the respective parental sequence was identified. (b) The average ω values (the non-synonymous to synonymous substitution rate ratio, dN/dS) based on branch models for the events affecting the third domain of parents and children, respectively. (c) The distribution of sites with significant signals of evolutionary selection reported by best-fit site models selected by the LRT (likelihood ratio test). The left adjacent subpanels reflect the same information as presented in (a). The site color represents whether the site is positively selected, conserved, or relaxed. The intensity of the color depends on the probability, ranging from 0.95 to 0.99. The right subpanel illustrates the significant model on branches with recombinants marked, namely, positive and purifying selection either for the foreground or background if the ω on branches is equal. (d) The percentage of positively selected and conserved sites determined using branch-site models for recombinants and parents within the events in which the third domain was transferred. (e) The relationship between the set of common species that recombinants and parents are active against and the ω, or the number of positively selected sites (f), obtained from using branch and site models, respectively. The color represents the domain transferred, whereas the shape of the dots encodes the domain for which the analysis was carried out.

To infer the locations of evolutionary selection signals, we applied site models and identified 228 significant signals corresponding to the conserved and positively selected regions (Fig. 5c, Supplementary Text S2.27, Figs S53a–S54f, Table S103). A total of 59% of sites were subjected to positive selection (Fig. S53a, Table S103). We discovered that when the first and the third domains are exchanged, a higher fraction of conserved sites occurs in domain II (Supplementary Text S2.17, Fig. S53b–e). A positive selection was found on sites, but domain conservation was identified on branches; therefore, we carried out a joint analysis to study selection in sequences in the context of recombination. We detected a total of 246 branch–site-based signals clearly showing negative selection, like the site-based ones (Supplementary Text S2.28, Figs S55a–S56d, Tables S102–S103). Generally, the conserved sites prevailed with a slightly higher (*P* < .01) percentage of positively selected sites in the first and third domains in recombinants and parents when domains I and II were transferred, respectively (Fig. 5d, Supplementary Text S2.28, Fig. S55c and d).

To reveal the functional impact of purifying selection force in chimeric toxins, we examined whether the selection signals display host specificity changes. We found no relationships between the evolutionary selection and the changes in the affected host orders (Supplementary Text S2.29, Fig. S57a–e, Table S102), whereas the overlap coefficient in the context of host species provided a finer picture. In terms of branch models only, the selection strength was higher (the ω was lower) in toxins with common affected species (Fig. 5e, Fig. S57f, Table S102). In contrast, the negative correlation (*P* < .05) between the similarity of host species and the number of conserved sites (Fig. 5f, Fig. S57g and h, Table S102) implied that severe changes in host specificity are accompanied by intensive negative selection. Because recombination substantially affects the topology of phylogenies, we propose that site-based approaches characterize evolutionary selection in recombination-prone toxins more accurately than branch models.

By using distinct types of phylogeny and sequence-based evolutionary selection analysis, we revealed higher purifying selection pressure accompanying drastic changes in the host specificity spectrum. We suggest that negative selection serves as a major force in conserving the most efficient chimeric toxins. Strong purifying selection represents a potent tool to thrive in specific ecological niches, since selected exchanges are preserved as they maintain the adaptive potential of the strains.

## Discussion

Recombination is considered one of the key evolutionary forces in the bacterial world. Apart from maintaining genomic diversity, this phenomenon increases fitness, enabling organisms to thrive in novel environments [66]. In the case of Cry toxins, the evidence supports that HR orchestrates the formation of hybrid toxins through domain shuffling, enabling *Bt* to be a successful pathogen and colonize novel hosts [18]. In this work, we conducted a comprehensive study of Cry toxin sequences and detected 50 recombination events, predominantly referring to the third domain (Fig. 2a). We demonstrate that the exchanges also engage the first domain (Fig. 2a, Table 1) with >70% of *cry* loci in natural diversity being affected by domain swaps (Fig. 4b and c) and demonstrate the permanent flux of domains (Fig. S12). By leveraging bioinformatic approaches, we unveiled the mechanisms by which the immense diversity of distinct toxins is formed and which strategies Cry-producing isolates follow to occupy ecological niches.

As early as 1986, the chimeric nature of Cry toxins was proposed [19]. Later domain III swaps were explicitly introduced in 1997 [23] and detailed in 2001 [18]. Since then, not many examples have been described. In our dataset, the known instances modified with new instances from BPPRC were detected as three events (Table S12) with domain III exchanges, namely Cry1Cb (Cry1Ca as major parent and Cry1Eb + Cry1Be1 as minors), Cry1Fa (Cry1Fb and Cry1Ka + Cry1Ig), and Cry9Ga (Cry9Aa and Cry1Gb1 + Cry1Ga2). The other 47 events were undescribed examples, 32 of which affected putative toxins. We do not claim to extend the accepted nomenclature and aimed to broaden understanding of Cry toxin evolution; however, this observation illustrates the potential of including toxins with appropriate domain composition and, in certain cases, known toxicity spectrum, thus fitting the accepted criteria (Supplementary Text S2.2, Table S45). In addition to quantitative assessments, we showed that Cry toxins act as donors and acceptors simultaneously (Fig. 2a) and uncovered an intricate network of domain exchanges (Fig. S12).

The impact of domain swapping remains vague because the experimental ramifications for toxins range from enhancing activity to losing it [62, 67, 68]. To this end, we harnessed all available data on host specificity (Table S45). The functional effects of individual domain exchanges in Cry toxins are broad, ranging from preserving host range to visible changes relative to minor and major parents (Tables S49–S50). Regarding the third domain shuffling, Cry71Aa is active against *Spodoptera exigua* as a domain III donor Cry72Aa [69]. Similarly, the parasporin Cry41Ab retained toxicity toward cancer cells as its major parent, Cry41Aa, after obtaining the third domain (Table S12) from AXY11484.1 (57% identity with Cry32Ma). Contrarily, Cry52Ba affects Diptera, unlike Lepidoptera-specific Cry52Ca, providing domains I and II [70, 71]. The Cry1Ig toxin showed toxicity to lepidopteran pests similar to the minor parent Cry1Ka, in contrast to the wide host range major parent Cry1Ia associated with the orders of Coleoptera, Lepidoptera, and nematodes [62, 72, 73]. Likewise, hybrid Cry4Ba forms affect dipterans [74] as their distant major parent Cry19Aa [75], whereas donors of the domain III from the Cry30 family also exhibited anti-lepidopteran activity [76]. Combining toxicity spectra with strain-wise data (Table S50) allows us to predict specialization. For instance, in the strain JC291, producing Cry1Dc activity against Lepidoptera only was reported [77], similar to the major parent Cry1Da [78], in contrast to broad-host-range minors of the Cry1A family active against the orders of Lepidoptera, Diptera, and Hemiptera [79, 80].

Shuffling of domains I and II deserves attention, since they remain poorly documented, unlike domain III swapping. Three of four exchanges of the second domain turned to represent chimeras of three domains simultaneously, and the remaining event was characterized by lower similarity between parents and children (Supplementary Text S2.6, Fig. S14, Table S12). Therefore, the exchanges likely mirror multiple sequential swaps. We do not exclude the possibility of these swaps, although they are evidently rare. We identified 13 events engaging domain I (Table 1). Generally, the range of affected hosts on a species level when comparing toxins from events was highly similar (Fig. S28–S29a). Nevertheless, distinct examples indicate that the consequences of the exchanges vary. For example, the swap of the domain I from AXE15628.1 (85.6% identity to Cry14Ab) to Cry14Ab increased toxicity toward *Caenorhabditis elegans* twofold compared to the major parent Cry14Aa with EC50 of 7 ug/mL and 16 ug/mL, respectively [67]. At the same time, Cry2Am is toxic to *Aedes aegypti* in contrast to the major parents Cry2Ag + Cry2Ab, affecting multiple species of Diptera and Lepidoptera orders and lepidoptera-specific Cry2Al [81–83]. The found instances fit in with natural hybrids in the Cry2 family found by PCR screening, followed by experimental shuffling that proved the role of domain I in determining host specificity [22].

Certain well-studied toxins of Cry toxin families were seemingly unaffected by recombination events. We propose that such observations could stem from the multi-step filtration of the most plausible events and incomplete attributions of toxic activities. Toxins of three families (i.e. Cry3A, Cry5A/B, and Cry1A) were predicted to engage in domain shuffling; however, they were filtered out as partial exchanges and/or phylogenetically incongruent events (Tables S10–S12). Moreover, these toxins served as parents were frequently marked as major parents, whereas single domain-based comparisons revealed that they could not be defined as true majors due to local similarity with only one domain (Fig. S10a). We could not exclude that they occurred after recombination, albeit these events are likely ancestral and not traceable, which is consistent with the predicted diversity of the unknown Cry protein holotypes (Supplementary Text S1.5). Thus, despite collecting all the available data on Cry sequences and host specificity, we should admit that there are many white spots, and we believe that the reported events, both raw and most plausible, could serve as a roadmap to further clarify the mechanisms to adjust host specificity.

Using clustering of short conserved regions surrounding domains, we found that flanks of recombination breakpoints exhibit substantial similarity between parental sequences (Fig. 3f) and explicitly illustrated their associations with domain exchanges (Fig. S22b–e). Conserved blocks were generally viewed as structural regions [14]; however, they find application in designing chimeric toxins [84], implying their role in domain exchanges that were proposed to be a RecA-independent process [85]. We characterized the genomic surroundings of *cry* genes, revealing that they are flanked by IS (Fig. 3a). Previously, IS were suggested to guide the dissemination of *cry* loci [86–88]; however, the exact molecular mechanisms have never been proposed, and the role of IS in domain swapping has not been described. To disentangle the fundamental genetic bases governing the process of domain exchanges, we applied pan-genome-wide analysis and reported significant pangenomic associations between the inter-domain conserved flanks and the recombinases of the XerCD family (Table S44). These recombinases resolve dimers of circular DNA, both chromosomal and plasmid, and frequently surround mobile genes controlling their circulation in isolates [65, 89]. For instance, Xer recombinases controlled the transfer of *cry* genes in *B. thuringiensis* and *Lysinibacillus sphaericus* [90]. Recent research demonstrated that XerCD also directly targets IS [89], implicating that DNA regions flanked by these MGEs are transferred by XerCD activity.

The discovery of prevalent conserved regions and associations between them and XerCD proteins allows us to provide the molecular explanation of the origin of chimeric Cry toxins (Fig. 6a). Inasmuch as XerCD recombinases target IS, they were drivers of selfish IS propagation during plasmid exchanges, which did not provide an evolutionary advantage to bacterial isolates. At some point in time, a putative fusion between ancestral toxins formed a multi-domain structure; thus, the respective inter-domain sites stemming from IS became a powerful tool in enabling Cry diversity. The site-specific nature of domain swapping explains the striking inconsistency between the sets of produced toxins in the strains containing recombinants and parents (Fig. 4d). Therefore, domain exchanges could be considered a collateral process in plasmid exchanges that later became a critical tool in expanding the toxic arsenal by a kind of “insertion domestication”.

**Figure 6:**
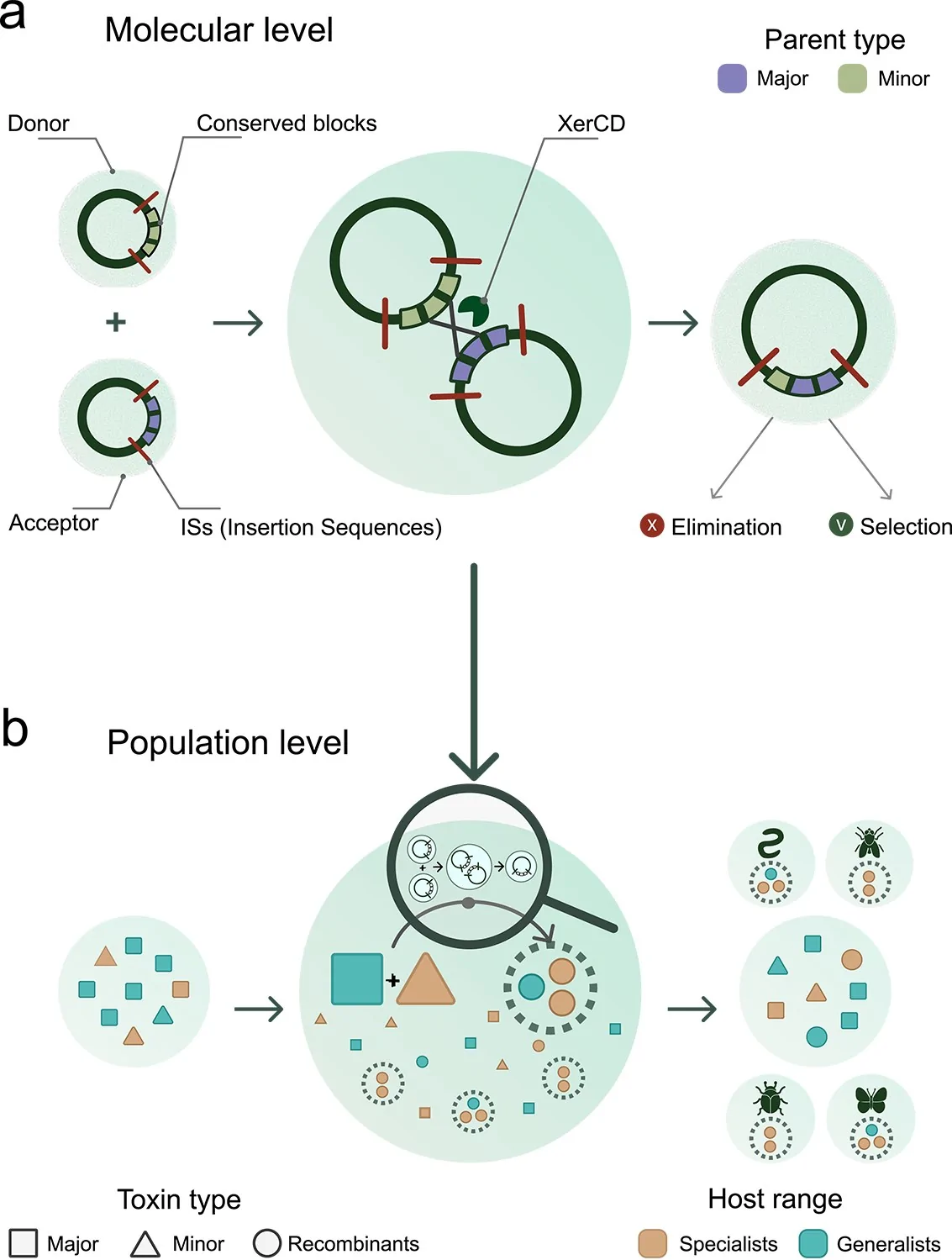
A general scheme uniting the mechanisms guiding the evolution of Cry toxins. (a) The model of molecular mechanisms controlling the formation of hybrid *cry* loci. The key process in the adaptation of Cry-producing isolates is the exchange of entire plasmids. The types of toxic genes are defined by the color. During conjugation, XerCD recombinases enable the resolution of circular DNA, including plasmids. Additionally, their ancillary activity lies in transferring insertions (ISs) flanking cry genes. The joint targeting of the conserved blocks with high similarity between the borders of the domains and insertion sites leads to the formation of hybrid loci. The resulting plasmids, either intact or those with chimeras, are then divided between the affected isolates. The site-specific nature of the origin of Cry toxins thus explains a visible dissimilarity in the produced toxins in parents and children. The hybrids are frequently non-functional and are eliminated, and the potent ones are conserved by negative selection. (b) The role of domain exchanges in the origin of specialist populations. Here, specialists are the isolates restricted to one host order. In contrast, generalists exhibit a wider host range, being toxic to more than one order. The strains are colored according to this classification. The shape defines the recombination-wise types of toxins in these strains. Although the most typical group of isolates is generalists bearing highly efficient toxins with a broad host spectrum, serving as major parents in recombination events, the sporadic occurrence of chimeras could limit host specificity, sparking the emergence of local specialist subpopulations. Initially, the subpopulations are not detached from the initial one; however, they accumulate toxins active against specific host groups and become isolated. In these small adapted populations, the exchanges do not alter affected orders and only change the host species range. Therefore, when analyzing Cry sequences and populations, we observe the prevalence of recombination events within specialists, whereas, in nature, the conversion of generalist major parents to specialist children is dominant.

We carried out a population-wise study to develop an original scheme elucidating the principal processes in recombination-affected isolates, because individual recombination events exert variable effects on host specificity (Fig. 6b). We identified the dominance of certain efficient toxins serving as major parents (Fig. S47a). Given the wide range of activities in these toxins, they are preserved in large groups that maintain their generalist nature, i.e. the ability to affect more than one host order (Fig. S47b). Domain swaps are likely to be non-functional, and only the beneficial ones remain through site-wise purifying selection (Fig. 6a), which has not been documented for Cry toxins previously, in contrast to positive selection [17]. Aside from Cry-producing bacteria, negative selection was reported to accompany recombination in pathogens and symbionts [91–93]. The presence of stabilizing selection on sites explains why some chimeras become fixed, unlike artificial hybrids that are eliminated [29]. In nature, novel hybrids could anchor specificity toward single hosts (Fig. S50b–d), resulting in local subpopulations still exchanging domains with generalists (Fig. 6b). Eventually, selected hybrid toxins, as well as other specialized toxins obtained after recombination, accumulate in these strains, forming isolated populations lacking contact with the initial generalist strains.

In contrast to branch and branch-site modes, examining evolutionary selection on sites only reported multiple positively selected sites (Fig. S53a–c), which is consistent with published studies employing site models [17]. Our data imply that the mutation process drives the evolution of Cry toxins alongside domain swapping, suggesting that the formation of new hydrids maintains the diversity of toxins, and the most potent ones are conserved in the population through negative selection on the whole domains. Positive selection, in turn, controls subsequent adaptation to hosts by point mutations modulating receptor–toxin interactions. Therefore, recombination-guided domain shuffling and point mutations both represent fundamental mechanisms of Cry-producing bacterial adaptation to hosts.

Pangenomic analysis [94] showed that *cry* loci attributed to the same hosts tend to co-occur and gather virulence factors enabling synergistic action against specific host groups. A significant association with mobile genetic elements, particularly IS [94], implies that the acquisition of these toxic moieties involves non-HR. Our results sufficiently extend these findings, demonstrating that acquiring novel toxins is tightly connected with domain shuffling. The more specialized the populations are, the more recombination-affected Cry toxins they produce (Fig. S48b). Along with *in vivo* experiments, we conclude that domain swaps are similar to mutations with variable effects, and it is the negative selection force that preserves host-specific toxins. Our model illustrates that domain swapping alongside obtaining whole *cry* genes is a key mechanism providing a source for specialization of bacterial strains to fit specific ecological niches and adapt to novel hosts within restricted areas.

By applying a comprehensive bioinformatics framework on a large scale, we provide a general evidence-based model elucidating the evolutionary trajectories of bacterial adaptation through preserving recombination-formed toxins with altered domain composition. The model is based on two simple principles discovered in this study: chimeric toxins evolve via XerCD-driven site-specific recombination, which are eventually filtered by strong negative selection, leading to specialization of the strains to specific hosts in isolated environments. This model deepens our understanding of how novel forms of Cry toxins are formed and preserved. Here, we analyzed the diversity of Cry toxins irrespective of the molecular techniques applied for constructing more potent toxins; therefore, we believe that our study, albeit being computational, is closer to the processes in nature. With the amount of evidence, we provide a new perspective on the evolution of Cry toxins. The formation of hybrids is more likely orchestrated by site-specific, but not homologous, recombination. On the population level, domain swapping with subsequent purifying selection results in a narrowed host range and specialization. Our findings demonstrate that these exchanges occur in bacterial species outside the *Bacillus thuginriensis* group, and all such events were characterized by numerous unknown parents, indicating the possible common origin of Cry toxins from domain shuffling of ancestral genes through this molecular mechanism (Table S104). Current studies report that multi-domain proteins, especially with toxic activities, have undergone multiple rounds of domain exchanges [95–100], whereas insertions were suspected to facilitate a site-specific horizontal gene transfer of chimeric loci [100]. Such phenomena are not constrained to bacterial species only. In coronaviruses, the receptor-binding domains (RBD) of the spike (S) proteins represent recombination hotspots; however, the events are relatively rare in present lineages, as recombination in their genomes is ancestral and leads to the emergence of distinct lineages [101]. The mentioned instances, in concert with our findings, prove that the recombination-assisted occurrence, dissemination, selection, and conservation of chimeric loci coding for host specificity and virulence determinants with subsequent specialization is universal for various pathogens across the tree of life.

## Supplementary Material

Supplementary_material_wrag218

## Data Availability

The resulting data are available in the Supplementary material. The raw data, including protein and nucleotide sequences, phylogenetic trees, required metadata, and source tables apart from those included in the Supplementary material, are presented at the git repository: https://github.com/lab7arriam/Cry_phylogeny.

## Code availability

The repository mentioned above (https://github.com/lab7arriam/Cry_phylogeny) also contains all the code used for data processing and visualization. All the required data and commands to run the scripts are provided to reproduce the results of the manuscript.
