# Supplementary material for: Site-specific recombination and purifying selection influence Cry toxin formation and specialization
Source: ISME J. 2026 Aug 26;20(1):wrag218. doi: 10.1093/ismejo/wrag218 (PMC13730063; doi:10.1093/ismejo/wrag218)
Supplement: Supplementary file 1 [file supplementary_material_wrag218.zip › Supplementary_clean.docx]

**Supporting Information for**

**Site-specific recombination and purifying selection influence Cry toxin formation and specialization**

Anton E. Shikov^1,2^, Ruslan O. Alagov^1^, Maria E. Belousova^1^, Anton A. Nizhnikov^1,2^, Kirill S. Antonets^1,2,*^

^1^Laboratory for Proteomics of Supra‐Organismal Systems, All‐Russia Research Institute for Agricultural Microbiology (ARRIAM), 196608 St. Petersburg, Russia;

^2^Faculty of Biology, St. Petersburg State University (SPbSU), 199034 St. Petersburg, Russia.

* **Correspondence**: Kirill S. Antonets, k.antonets@arriam.ru

**This file includes:**

Supporting text S1.1 to S2.29

Figures S1 to S57

Descriptions of Tables S1 to S104

SI References

**Supporting Information Text**

**S1. Supplementary Methods**

**S1.1. Initial data acquisition and processing**

All data-processing steps were performed using custom scripts written in Python v3.6.9. Statistical tests were carried out using the R v.3.6.3 programming language. Plots, phylogenetic trees, and graphs were visualized using libraries ggplot2 v3.3.5 [1], ggtree v1.16.6 [2], and pygraphviz v1.6 [3], respectively. Four datasets were the sources of Cry toxin sequences. These included protein sequences from the NCBI IPG database (Identical Protein Group, *Bacillus thuringiensis* taxonomy ID: 1428, accessed on the 3rd June 2020 (the same for all four datasets), 467 *Bt* assemblies from NCBI Assembly, BPPRC (Bacterial Pesticidal Protein Resource Center) [4], and NCBI Genbank (bacteria non-redundant). Three-domain Cry toxins were extracted with CryProcessor in the “-*do”* mode [5]. We used CD-HIT v4.8.1-2019-0228 [6] for deduplication, specifying the following parameters: word size of 5 and 95% identity threshold.

**S1.2. Analyzing Cry toxin sequences**

To get the nucleotide sequences of Cry toxins, a custom script implementing Biopython v1.73 [7] was utilized for identifying nucleotide accessions from the IPG annotations and downloading sequences. The validity of nucleotide entities was checked through the comparison of sequences translated via the Seqkit v0.10.1 utility [8] with the original proteins. The coordinates of the domains of Cry toxin clusters were obtained with CryProcessor v1.0, and the respective sequences were retrieved using the BEDTools v2.26.0 getfasta utility [9]. To exclude artificially modified toxins, we collected the sources of reference clusters based on all identical sequences from the IPG database. If the source of all entries belonged to the patents database, we additionally analyzed the descriptions of patents and discarded the clusters that were described as artificially modified toxins.

To get the overall descriptive statistics of Cry toxin sequences, we summarized the lengths of the domains and their mean sequence similarities. To estimate sequence conservation, we calculated the highest per-site frequency of a non-gap symbol in the multiple alignments of toxin sequences with a custom Python script and plotted the distribution accordingly. We also analyzed the mean pairwise similarities of the sequences within a cluster. Cry toxins were compared with the known diversity of toxins deposited in the BPPRC by calculating identity percentages with the closest toxin from the database. We paid particular attention to the clusters containing 10 sequences or more by characterizing the spectrum of mutations classified as synonymous, non-synonymous, and frameshift mutations and plotting their distribution on the toxin sequences.

**S1.3. Parameters for phylogeny reconstruction**

We aligned sequences of deduplicated reference clusters with the MAFFT aligner v7.429 [10] in the “localpair” mode with 1000 iterations and a gap penalty of 5. The alignments were trimmed with trimAl v1.4.rev22 utility [11] with the following parameters: (i) 30% minimum percentage of the positions in the original alignment to conserve, (ii) 0.3 minimum average similarity allowed, and (iii) the fraction of allowed gaps of 0.5 due to the high diversity of the Cry toxins. Best-fit evolutionary models were assessed by ModelTest-NG v0.1.7 [12] in the “ml” mode according to the BIC value (Bayesian information criterion). Maximum likelihood (ML) trees were reconstructed using RAxML-NG v1.1.0 [13] with 1000 bootstrap replications. Four phylogenies were based on the sequences of each domain and a full toxin core with three domains partitioned according to the boundaries of the domains. We started with protein-based trees, but retained nucleotide-based phylogenies due to better tree properties, namely, lower polytomy levels and higher bootstrap values. We compared the topologies of the reconstructed trees with the quartet distance metrics using the tqDist v1.0.2 software [14], Robinson-Foulds distance implemented in RAxML-NG [13], and cophenetic distance by the ape v5.6-2 package [15]. Tree quality metrics, namely consistency index (CI) and retention index (RI), were calculated with phangorn v2.5.5 [16], and tree balance indices, such as “CollessLike”, “Sackin”, and “Cophenetic” were identified using the CollessLike v2.0 package [17]. A graphical display of topological discrepancies was illustrated as tanglegrams produced by the dendextend v1.14.0 package [18]. We also plotted topological dissimilarity in a heatmap-wise way: the order of the toxins corresponded to the full core toxin-derived tree, with the values depicting the pair-wise identity of a certain domain.

**S1.4. Detecting and characterizing recombination events**

Three-step filtration of the detected events was carried out. We excluded events caused by evolutionary processes rather than recombination marked in the RDP4 output. After initial filtering, we considered only the events spanning more than 70% of the domain, and partial events were analyzed separately. After removing short sequences, we assessed the identity between the domains of parents and children and the phylogenetic congruence. Only the events in which the identity of the transmitted domain derived from a so-called minor parent (donor of the single domain) were higher than the same domain from the major parent from which the other two domains were obtained. To examine phylogenetic congruence, we transformed phylogenies as follows: we used the single domain-based trees and collapsed branches with bootstrap support of less than 70% and grouped recombinants and their parents in a single subclade (Fig. S1a). Subsequently, we checked if parents and children formed a compact phylogenetic branch on a transformed tree devoid of toxins unrelated to the analyzed event. We also updated the list of parents for a single domain if the clade contained extra toxins.

To assess the quality of filtering, we compared the identity difference between the transferred and non-transferred domains for RDP4-reported initial and refined datasets and compared the obtained distributions with the Wilcox test corrected for multiple comparisons with the Benjamini-Hochberg (BH) procedure. To reveal mutation rates in Cry sequences, we summarized the number of mismatches between recombinants and the respective major and minor parents using the BioPython v1.73 library [7] to assess the evolutionary changes following the exchange events. We also classified the traces of recombination events affecting the sequence composition of Cry toxins using fastGEAR [19], Gubbins v3.1.2 [20], and ClonalFrameML v1.12 [21] to calculate the recombination-to-mutation (r/m) ratio.

To disentangle multiple recombination events during the evolutionary history of Cry toxins, we employed two approaches to plot the map of recombination exchanges. We built a Circos plot using the circlize v0.4.13 [22] package to display each transfer between toxins arranged according to the reference tree. Using recombination event mappings, we created the unweighted directed recombination graph with a custom Python script implementing the networkX v2.4 [23] library and revealed connected components in the recombination graph.

**S1.5. Predicting the number of unknown toxins**

We assessed the number of toxins possibly absent in our dataset based on the proportions of recombination-affected toxins in trees based on the individual domains. To calculate the number of possible toxins representing unknown parents, we summarized the depths (d) of subtrees in a domain-wise way and obtained the sets of depths (D). The leaves with unknown events from collapsed trees were removed. We searched all subtrees with depth values in the respective trees and counted the number of nodes containing recombinants f(d) and revealed the number of leaves with parents p(d). In case unknown domains came from minor parents, we used the respective trees based on sequences of the transferred domains. For the instances related to major parents, we considered adjacent domains. We selected minor parents and major parents for adjacent domains, omitting the opposite ones to exclude distortion due to possible ancestral exchanges. For each event with parents of unknown origin, we predicted the number of toxins as follows: U(d) = n(d)*p(d)/f(d). Finally, we summed up all predictions roughly perceiving domains as full toxins, to get the total number of toxins potentially absent in the dataset

**S1.6. Assessing the relationships between domain swaps and mobile genetic elements**

To access the overall recombination rate, we reconstructed pangenomes using Panaroo v1.2.8 [24] of genome assemblies grouped according to the presence of *cry* genes and toxins subjected to domain swaps. Pangenome reconstruction was carried out with Panaroo v1.2.8 [24] with “--clean-mode strict” and “--remove-invalid-genes” options selected, and a 95% identity threshold for core genes with MAFFT aligner v7.429 [10] chosen for core genes extraction. The pangenomes were classified as open/closed according to the Heaps’ law alpha parameter. Estimates of the r/m ratio based on concatenated core gene alignments were obtained with the ClonalFrameML v1.12 program [21].

The genomic context of *cry* loci was analyzed by identifying mobile genetic elements (MGEs). Insertion sequences (IS) were detected with ISEScan v1.7.2.3 [25]. Prophages were predicted by Phigaro v2.3.0 [26], and genetic islands (GI) were found using the IslandPath-DIMOB v1.0.6 [27] utility. We collected the data on genomic coordinates for genes encoding Cry toxins and intersected them with the respective locations of mobile genetic elements (MGEs) using BEDTools v2.26.0 [9] getfasta. We further calculated the correlation between the number of different MGEs using the Pearson correlation coefficient and compared their abundance in assembly groups with the BH-adjusted Wilcox test. The portions of *cry* genes localized in the MGEs were compared with Fisher’s exact test.

**S1.7. Analysis of conserved flanks surrounding recombination breakpoints**

To identify conserved blocks in Cry toxin sequences, we took positions of breakpoints in the parental sequences from the RDP4 output and aligned sequences of parents within the events with subsequent excision of 100 bp regions downstream and upstream of the breakpoints. Two types of flanks were selected, namely, those located between the boundaries of the first and the second domains, and the second and the third domains, respectively. We calculated site-wise identity between parental sequences and ran the “rollmean” function from the R package zoo v1.8-9 [28] with center alignment and a window size of 7 accordingly. For clustering these blocks, we chose an optimal clustering pipeline using silhouette analysis implemented in the factoextra v1.0.7 [29] package and utilized it accordingly.

To perform clusterization of regions flanking recombination breakpoints, three metrics were used, namely Euclidean, cosine, and Manhattan distances. The dissimilarity matrices were built based on vectors representing per-site identities, both raw and with the roll mean procedure applied. Additionally, two clustering algorithms, such as hierarchical clustering and k-means, were tested. The best clustering pipeline was selected according to silhouette scores calculated with the factoextra v1.0.7 [29] package. The distribution of per-site identity within clusters was inspected to manually select peaks with high identity. The mean identity estimates in these peaks were compared with the overall similarity between transferred and adjacent domains using the Wilcox test.

**S1.8. Identifying molecular mechanisms of domain swapping**

To retrieve conserved regions in *cry* loci, the approach based on k-mers was employed. For each set of parental sequences in the upstream and downstream regions (100 bp) surrounding the recombination breakpoints, we used a sliding window with a k-mer size of 11 bp and evaluated the similarity within each k-mer. We specified the identity threshold as the median identity plus standard deviation for the estimates of all the k-mers in a certain recombination event. We picked the continuous k-mer chains satisfying the thresholds, chose those corresponding to sequences with at least 15 bp in length, and selected regions closest to the breakpoints.

Clusterization of the most conserved flanking sequences was performed to identify families of highly similar regions related to recombination. We chose the most appropriate method for the hierarchical clustering procedure with distinct statistical approaches. We tested a combination of multiple approaches based on the following parameters: (a) the aligner, namely, MAFFT v7.429 [10] and the default pairwise aligner implemented in the BioPython v1.73 library [7]; (b) alignment mode (global and local); (c) similarity evaluation method, namely global (i.e., the number of matches normalized by the length of the alignment), and local (matches divided by the length of the smaller sequence); (d) distance metrics, such as median, Mcquitty, centroid, complete, average, single, Ward.D, and Ward.D2; and (e) the number of clusters (from 5 to 10). To inspect the best clustering method, we considered several characteristics, namely, (a) the total size of the clusters; (b) the number of leaves in the subtrees from the dendrogram, containing all parents from a certain event; (c) the similarity between sequences in the cluster; (d) clustering quality estimates (Dunn, silhouette, and gap statistics) calculated using the factoextra v1.0.7 [29] and NbClust v3.0.1 [30] packages; and (e) the topological similarity with the reference ML phylogeny. The latter was reconstructed, comprising all conserved sequences followed by the alignment with MAFFT v7.429 [10] in the “localpair” mode with 1000 iterations. The ML tree was obtained using RAxML-NG v1.1.0 [13] launched with 1000 bootstrap replications. The topological similarity was assessed according to the quartet distance determined by the tqDist v1.0.2 [14] utility. We defined the best method that gained the set of clusters with the highest quality estimates, similarity to the reference phylogeny, and in-group identity, as well as the lowest range between the number of participants in the cluster and the number of leaves in the subclades uniting sequences from the same events.

Clusters of flanking regions located near recombination breakpoints were applied to build consensus models. To increase sensitivity, we focused on all toxins constituting the reference clusters generated by CD-HIT v4.8.1-2019-0228 [6] with a 95% identity threshold. All the representatives for the flanking sequences were disclosed for each conserved sequence and re-aligned with MAFFT v7.429 [10] with subsequent excision of the identified flanks. The sequences from each cluster were aligned again using MAFFT v7.429 [10] in the local mode. To ensure that the procedure does not affect the properties of the clusters, we compared the distributions of lengths and pair-wise identities with reference-only and extended clusters with the Wilcox test adjusted for multiple comparisons. Consensus models were reconstructed with the GLAM2SCAN v5.5.7 software from the MEME suite [31]. The suite was applied to search the flanks in all deduplicated nucleotide sequences of available Cry toxins. Raw signals were filtered according to the length and the identity of the hit with the cluster members. We picked only those regions that showed at least 70% and at least 70% of the total length relative to the best-match representative of the cluster. We then compared the similarity between flanks attributed to certain models in toxins classified according to their role in recombination events, considering parents, recombinants, and pairs of children/parents from the same events and other toxin pairs, either unaffected or from different domain exchange events. Statistical significance was inspected using the Wilcox test, followed by the Benjamini-Hochberg adjustment method. To explicitly show the link between the identity within present flanking regions and engagement in domain-swapping events, we used logistic regression to find cluster-wise dependence between the similarity of the flanks and participation in the same recombination events. The probabilities and significance were evaluated with the effects v.4.2-2 [32] package.

To identify pangenome-wise associations between the presence of flanking regions in bacterial genomes and specific accessory genes, we used the pangenome with all available *Bt* assemblies. Genomes were classified according to the consensus-wise signals with conserved flanks corresponding to distinct clusters of conserved regions. The presence of the cluster was attributed to an assembly as a trait if at least one *cry* gene encoded in the respective genome contained the signal(s) identified with GLAM2SCAN v5.5.7 [31]. The distribution of traits was then used to reveal pangenome associations with the Scoary2 v0.0.15 program [33]. We selected significant associations according to Fisher's *Q* values and empirical *P* values. Positive associations were filtered based on the allele frequency (at least 70% of genomes with a trait should contain the studied gene) and odds ratio (the percentage of the genomes with both trait and a pangenome cluster should be not less than 70% of the cluster-positive genomes).

**S1.9. Revealing the impact of domain swaps on host specificity and toxin composition**

To determine the influence of recombination on host specificity and toxin composition, we collected the metadata from the IPG database and attributed the toxins to the strains in which they are present. We then gathered the spectrum of activities from BPPRC and exhaustive reviews [34–36] and manually searched for research articles and patents to ascertain the hosts infected by the toxins as well. The information obtained was attributed to toxins based on the third rank according to the current nomenclature, and strains in case individual proteins were not tested. The percentage of orders to which individual species pertained was displayed as event-wise scatterplots via the scatterpie v0.1.8 [37] package.

To obtain the ancestral state host specificity spectra for the toxins, we used the phylogeny-wise procedure by reducing the host-annotated tree encompassing all parents of a certain type or recombinants within a specific recombination event. We chose trees corrected for recombination (Supplementary Text S2.4). For minor parents and recombinants, the trees based on the transferred domains were used. To deal with major parents, we considered the phylogenies of the adjacent domains. We performed the ancestral reconstruction procedure according to the following principles: (i) if the affected hosts were unequal, the resulting merged node contained both orders (e.g., Lepidoptera|Diptera); (ii) in case intersections were present, only common hosts were taken; (iii) when polytomy occurred, the annotations with the highest frequency were taken. The final annotations for the reduced nodes were saved for further analysis. The nodes of the trees were reduced with the ETE3 v.3.1.2 package [38].

To assess the similarity between activities of recombination-affected toxins, we compared toxic activities. The sets of affected hosts of recombinants and parents were compared using data for toxins and strains. We examined changes in orders, treating events as different if parents and recombinants had at least one uncommon order. To study the effect of recombination on the composition of toxins, we compared the sets of toxins present in the assemblies/strains comprising recombinants and parents using the Jaccard coefficient (the number of intersections divided by the length of the union). We summed up the number of toxins in the respective isolates, as well as in those devoid of recombination participants, and compared them by applying the Wilcox test adjusted for multiple comparisons. We performed similar comparisons in terms of the number of affected hosts and species. To exclude the possible impact of set size, we utilized Szymkiewicz–Simpson metrics (the number of intersections divided by the minimum length). Linear models were used to find factors (number of toxins, identity between domains, parent type, transferred domain) possibly delineating the intersection between the sets of hosts, and binomial regression to study the impact of factors on changes in the affected orders. To identify correlations between certain factors, we applied the Pearson correlation test, followed by *P* value correction using the FDR (False Discovery Rate) method.

To calculate the significance level of the recombination events impact on host specificity, we employed a bootstrap-like approach. We used several steps: (i) on each iteration, we calculated the difference ratio (e.g., the percentage of pairs marked as different); (ii) for a given dataset of n observations, we picked samples from n-5 to n from the observed dataset or randomly generated vector with ones and zeroes; (iii) for each sample, we calculated the difference ratio and repeated the operation 1000 times; (iv) experimental subsampling estimates and randomly generated ones were compared using the Wilcox test adjusted with the FDR procedure.

To study relationships between toxin composition and genomic context, we picked assemblies in which at least one recombinant and parent for the particular event co-occurred and visualized the genomic localization of MGEs and regular loci using the Proksee [39] web-based service. For revealing bacterial population with shared toxin content, we constructed a weighted graph using the networkX v2.4 [23] library with the nodes corresponding to strains, with the edges containing the list of toxins shared by the pair of strains, and the weight indicated the number of common toxins. We identified which connected components harbored the parents and the recombinants and compared the sets of hosts using both Jaccard and Szymkiewicz–Simpson coefficients.

To globally analyze if recombination broadens or narrows the host range, we classified strains and connected components in the strain graph depicting isolates with similar toxin content as generalists and specialists. Generalists affected more than one host order. Connected components were considered generalists if at least 70% of included strains were generalists. Using classified isolates and connected components, we explored global recombination strategies by assigning the list of toxins relative to their role in recombination events to the generalist/specialist group by summarizing the respective characteristics of the strains/connected components containing such toxins. The whole group was considered a generalist in case 70% of the strains/connected components in the set were generalists. The strategies were encoded by three letters in the following order: recombinants, minor parents, major parents. For example, the GGG strategy implies that the isolates containing recombinants, minor parents, and major parents were all generalists, respectively. The strains/components realizing distinct strategies were compared, considering the number of toxins produced and affected hosts.

**S1.10. Preparing the data for evolutionary selection analysis and assessing selection pressure**

We developed a multi-step pipeline to prepare the data for such calculations due to the high computational resources required for evaluating evolutionary selection (Fig. S1b). Before obtaining guiding trees and alignments, we discarded 100% duplicates for sequences of the domains within the reference clusters. We analyzed each of the domains separately. To obtain the background when dealing with recombination events, we set a threshold determining how many sequences should be included, no less than twice as many toxins as the sum of recombinants and parents of a certain domain. We utilized the ETE3 v.3.1.2 package [38] to get a subtree containing the common ancestor of recombinants and parents, and iteratively moved from the subtree to the upper nodes to get a set of sequences satisfying the threshold. During the procedure, we disclosed CD-HIT-based clusters of Cry toxins with a 95% identity threshold picking 5 random toxins or fewer for the cluster in case it incorporated more sequences. If the set satisfying the threshold exceeded 50 representatives, we collapsed nodes marked by bootstrap support of less than 70%, arranged nodes according to the similarity of protein sequences to the respective recombinant sequences, and iteratively added nodes until the length threshold was reached the required threshold.

To analyze evolutionary selection, we used optimal subtrees and domain sequences constituting background and foreground for each recombination event. For each event-wise data, we determined the optimal evolutionary model using ModelTest-NG v0.1.7 [12] and prepared the guiding tree with RaxML-NG v1.1.0 [13]. Because assessing evolutionary selection operates with codons, we aligned protein sequences with MAFFT v7.429 [10] and matched the nucleotide sequences to protein alignments with a custom Python script. We examined three types of models using the ETE3 v.3.1.2 package [38] (Fig. S1c). We applied two site models, “bneut”, and “btree”, indicating neutral selection and positive/negative selection, respectively, marking leaves harboring either recombinants or parents. Forasmuch as domain-wise parents were considered, we took all parents within the domain without omitting those that are not true majors. For detecting alterations in selection patterns over branches, we opted for the models exhibiting significantly different LRTs (likelihood ratio tests) when comparing “M0” with “b_free”, and “b_free” with “b_neut” models, implying different ratios on branches and that the evolution in the foreground branch is not neutral, respectively. Inferences on account of the non-synonymous to synonymous substitution rate ratio (ω = dN/dS) value were marked as being subjected to positive selection if the ω value on the foreground exceeded 1 and to purifying selection otherwise. Apart from branch models, different site models were examined, namely, “M0”, “M1”, “M2”, “M3”, “M7”, and “M8”. The sites, both conserved and positively selected, were aggregated for the best-fit models determined by the likelihood. To address both tree topology and sequence characteristics, we considered branch-sited models, namely, “BsA”, “BsA1”, and “BsB”, and summarized both sites and branch-based ω values for the events with significantly detected selection. To find relationships between different factors, including the number of toxins, ω on foreground and background branches, as well as the number of conserved and positively selected sites, we deployed linear regression. We also studied whether these factors delineate the sets of hosts using the same method accordingly.

**S2. Supplementary Results**

**S2.1. Cry toxins show an unequal mismatch rate in domain III sequences, indicating strong allele flow**

Of 941 unique Cry toxin sequences, 351 represented undescribed groups absent in the BPPRC (Fig. 1b, Tab. S1), with 203 of them being substantially similar (>97%) to the closest entity from BPPRC, implying point mutations and/or sequencing errors. In contrast, within reference clusters based on the 95% identity threshold, 93 toxins were less than 95% similar to the closest homolog, reaching 64% on average (Fig. 1c). According to pairwise identities between domains, the first one was slightly more conserved (53.2%) than the second (50.2%) and the third (51.8%) (Fig. S2a, Tab. S2). In addition, the first and the second domains were longer (612 bp on average) than the third one (418 bp) (Fig. S2b, Tab. S2) and contained a higher fraction of conserved sites (Fig. S2c, Tab. S3). There was no correlation between domain lengths and mean identity (Fig. S2d). Most of the clusters were singletons (298) or doubletons (24); however, 7 clusters included over 10 toxins (Fig. S3a, Tab. S1). Within-cluster pairwise identity between the sequences of the domains exceeded 99% and was independent of the cluster size (Fig. S3b-d, Tab. S4).

To explore the properties of frequent Cry protein groups, we summarized codon-wise differences in the cluster alignments. Non-synonymous mutations exceeded synonymous ones in all the domains (Fig. S3e-f, Tab. S5). Despite the first and the second domains harboring more substitutions (Fig. S3f), after normalizing the number of non-synonymous mutations by the length of the domains and the number of toxins, the third domain was more divergent, reaching 0.00051 compared with 0.0003 and 0.00035, respectively. (Fig. S3g). Differences between individual domain sequences indicate a more intensive mutation rate and/or recombination, causing greater allelic diversity.

**S2.2. Current databases contain toxins that can be included in the existing nomenclature**

To characterize the landscape of domain swaps between Cry toxins, we expanded the dataset through mining sequences bearing three functional domains and obtained 368 clusters based on the 95% identity threshold, with 110 of them sharing less than 95% homology with known sequences from the BPPRC database (Fig. 1c). A total of 30 toxins were attributed to other species and genera, with 14 of them marked as novel ones, and some being substantially different from the BPPRC references, such as toxins from *Clostridium botulinum* and *Brevibacillus brevis* with identity estimates of 32% and 23% to Cry4Ba1 and Cry1Ie2 sequences (Fig. S4a, Tab. S6), respectively. No interdependence between the length of the domain and the identity with the closest deposited reference was found, indicating that local identity metrics are not disturbed by unequal length, thereby excluding that they occurred through duplication but encountered other evolutionary mechanisms (Fig. S4b).

Analysis of all available Cry toxins with undescribed sequences shows that our computational approach sufficiently extends the list of chimeric toxins. It is not our aim to expand the existing nomenclature; however, it might be useful to consider some instances for reviewing and naming. Current criteria for consideration imply either >95% identity with the existing proteins or experimentally demonstrated activity against invertebrates [4]. Although self-titled toxins found in genome assemblies such as Cry13Ba1 [40] do not meet any of the criteria, some proteins like Cry3Aa12_81.9_AEZ53144.1 [41] were proven to form crystals and were toxic towards different hosts, and are worth consideration. The presence of Cry toxins in non-*Bt* species like *P. bifermentans* and *Paenibacillus popilliae* is a commonly known observation [42, 43]. We, however, revealed the presence of these toxins in diverse species within the *Bacillus* genus, members of the *Bacillales* order, and, most intriguingly, *Clostridium* *botulinum* (Fig. S4a), which corroborates the existing studies [44, 45]. Taxonomic distribution of Cry-producing bacteria allows us to assume the possible common origin of these toxins. Undescribed toxins and production of Cry proteins by non-*Bt* species underline the abundance of undiscovered insecticidal potential yet to be harnessed, and that at least some of the toxins deserve to be revised for inclusion in the current nomenclature.

**S2.3. Cry sequences and phylogenies hide multiple traces of recombination events**

Properties of domain-wise trees illustrate the effect of recombination events. Aside from the low consistency index (CI), the third domain-based tree possessed the shortest branches (Tab S9). Recombinant and parental sequences group on the respective tree related to the swapped domain, whereas in others, these recombination events are left unaccounted, causing intense homoplasy and distortion of the molecular clock, leading to longer terminal branches [46]. Our observations indicate that the recombination-driven domain III swapping is the most widespread type of exchange. Altogether, the results presume that the evolution of individual domains is independent. This extends the previous observations [47–49] and demonstrates that the swapping is not confined to the third domain only. These findings support the incongruity between tree topologies, disturbed branch lengths, and extreme homoplasy rates. Therefore, the results provided strong evidence that recombination affected the phylogenetic inferences, showing that domain exchanges drive the evolution of Cry toxins. These data corroborate the unequal mismatch rates in the domain sequences and the landscape of domain-wise identity estimates being incongruent with the topology of the reference tree.

**S2.4. Applying sequence- and phylogeny-based filters to infer the most probable recombination events**

To study the presence of domain shuffling, we utilized the RDP4 software and revealed 120 raw events (Tab. S10). After obtaining raw recombination signals, we performed a two-step filtration based on the length of the transferred region and the phylogenetic congruence between parents and children on the domain-based trees (see Supplementary Text S1.4). The number of reports was then reduced to 100 and 50 events, respectively (Tab. S11-12). There were more exchanges of the third (33) and first (13) domains. Only 4 events of the second domain were detected (Fig. 2a, Fig. S7a-c). Most of the breakpoints were arranged around the boundaries between the domains (Fig. 2b, Fig. S7d-f). The filtration allowed discarding the events with a large number of participants (Fig. S8a-b, Tab. S13), decreasing the percentage of unknown parents (Fig. S8c), and, conversely, increasing the similarity between recombinants and parents within the transferred regions (Fig. S9a, Tab. S14) as well as the difference with non-transferred domains (Fig. S9b, Tab. S15). This procedure lowered the number of minors and, conversely, leveled up the abundance of majors (Fig. S10a, Tab. S16). We also found that 14 events were characterized by more parents providing only a single domain, which most likely evolved from ancestral exchanges, especially engaging the third domain (Fig. S10b).

**S2.5. Cry sequences, per se, show a high recombination rate and hide putative ancestral exchanges**

To describe sequence differences between parents and children in recombination events, we analyzed the domain-wise number of mismatches between parents and recombinants within the transferred regions, normalized by the alignment length. There was a significant difference (*P* < 0.05) between the mismatch rate of minors and recombinants in the third domain compared with the respective rate within the first and the second domains between majors and recombinants, reaching the median values of 0.23, 0.14, and 0.18, respectively (Fig. S10c, Tab. S18). Inconsistent mismatch rates show intensive swapping of the third domain affecting both recombinants and parents in other putatively unaccounted events that are supported by the inflation of parents providing the third domain when correcting the list of parents depending on the content in the subtree of domain III (Fig. S10b) and by the higher normalized non-synonymous ratio in the clusters of toxins grouped according to the 95% sequence identity threshold pooling more than 10 toxins as well (Fig. S3g). The results prove that recombination sparks elevated levels of allelic diversity, and the more frequently exchanges happen, the more non-synonymous mutations emerge.

High frequency of domain exchanges is consistent with high global recombination rates calculated on Cry sequences. We studied the recombination rate using ClonalFrameML, Gubbings, and fastGEAR, which reported 13290, 369, and 1711 events, respectively (Fig. S11a-c, Tab. S19-21). The programs provided incommensurable ratios of effects of recombination and mutation (r/m), namely, 372.2 and 1.1, estimated by ClonalFrameML and Gubbings. Due to the high number of events, the latter seems to be an underestimation. A substantial incongruence might be explained by inaccurate predictions when dealing with extremely divergent and intensively recombining sequences [46]. However, the abundance of the reported domain swapping events in all cases, per se, mirrors the presence of extensive recombination between Cry toxins.

**S2.6. Linked recombination events suggest that the natural diversity of Cry toxin sequences remains poorly studied, and ancestral domain exchanges between the isolates**

To reveal traces of ancestral recombination events, we scrutinized the graph of domain exchanges. The combination of two approaches, namely, adding parents from domain-wise phylogenetic trees per recombination events and creating the exchange graph, was used for spotting linked events (Fig. S14, Tab. S24). In contrast to regular events with minor and major parents, toxins within such linked exchanges received each of the domains from distinct parents, possibly implying that true major parents are absent in the dataset of currently available sequences of Cry toxins. There were two events involving the second domain, which suggests that such findings could arise from two sequential swaps of the first and the third domains. Because two regular events in which the transferring of the second domain was more evident were present (Tab. S12), we do not exclude the probability of domain II exchanges; however, they seem to be quite rare if they exist. All the toxins representing CD-HIT generated clusters with a 95% identity threshold that included more than 10 representatives (Fig. S3e-g) were present in the recombination graph and belonged to the parental group. Therefore, we suggest that these toxins are conserved in the population and represent sources for novel sporadically occurring combinations of the domains. The fact of the continuous flow finely corroborates the altered mismatch rates both in the within-cluster sequences (Fig. S3g) and parents regarding individual recombination events (Fig. S10c), coupled with the inflation of single-domain parents when inspecting domain-wise phylogenetic trees (Fig. S10b).

**S2.7. The genomic analysis reveals that homologous recombination does not determine the process of domain swapping**

To revel mechanisms driving domain exchanges, we assessed the HR (Homologous Recombination) rate based on *Bt* genomes. We reconstructed pangenomes of 467 *Bt* assemblies, with 169 comprising Cry toxins and 65 including parents/children in recombination events. All the pangenomes were rich in accessory components, and only 7-10% of core genes were reported (Fig. S15a-c). They were open due to the alpha parameter ranging from 0.6 to 0.7, implying high genetic plasticity (Tab. S25). The ratio of rates of recombination and mutation (R/θ) ranged between 0.07 and 0.3, indicating that mutations, in general, happen more often than recombination (Tab. S26). However, the r/m scores were higher, reaching 2.5, 2.4, and 0.9 for the datasets, namely, all *Bt* assemblies, those having *cry* genes, and genomes harboring participants of recombination (Fig. S15d-f, Tab. S26), which indicates the different effect sizes. Nevertheless, the tendency and lower values in the genomes containing *cry* loci subjected to recombination were preserved.

We did not show direct relationships between HR and domain exchanges; thus, we proceeded with studying mobile genetic elements (MGEs), namely, prophages, genomic islands (GIs), and insertion sequences (ISs). The number of these elements was significantly (*P* < 2.2e-16) correlated, because the Pearson coefficient hovered between 0.39 and 0.49 (Fig. S16a-c, Tab. S27). Therefore, if the genome is rich in some groups of MGEs, it will likely house other classes as well. Assemblies harboring *cry* loci both with or without domain swaps, incorporated a significantly higher portion of MGEs (*P* < 1e-08) compared to all assemblies, and no difference between the former two groups was detected (Fig. 16d). We identified that of all 602 toxins, 172 Cry-encoding genes resided in GIs, and 15 were located within IS, whereas no relations with prophages were reported (Tab. S28). A total of 36% of *cry* genes that had undergone recombination were associated with GIs and IS vs. 28% of all toxins (Fig. S16e). However, Fisher's exact test demonstrated no significant difference (*P* = 0.1), indicating that these loci could hide unaccounted domain exchanges.

**S2.8. Characterizing the overall distribution of conserved blocks suggests unequally distributed distinct families**

To study the sequence identity within recombination breakpoints, we selected recombination events without unknown parents and aligned the respective sequences to get the coordinates of breakpoints in parental toxins. We re-aligned the parents only and, through mapping to the initial coordinates relative to full sequences, excised 100 bp flanking regions surrounding the breakpoints (Tab. S29). After that, we ran the rolling average function on the coordinate-wise per-site identity. A more detailed picture of the event-wise distribution of identity scores was obtained by clustering the respective distributions. We opted for the most suitable clustering algorithm and distance metrics by determining the highest silhouette score. The best option implied cosine distance applied on rolling mean-corrected identity scores with the following hierarchical clustering (Fig. S17a-b, Tab. S30). The optimal number of clusters reached 2 and 6 for the first and second breakpoints, respectively (Fig. S17c-f, Tab. S31). According to the inter-cluster identity distribution, distinct clusters displayed from one to three peaks offset from the breakpoints (Fig. S18a-b). The average within-peak identity constituted 81% and 73% for the first and the second breakpoints vs. 62-68% and 54% for the respective non-transferred adjacent and transferred domains (Tab. S32, Fig. S18c). The comparisons were significantly different according to the Wilcox test (*P* < 0.05).

**S2.9. Reconstructing distinct models for certain flank sequences in the inter-domain regions**

To characterize flanking regions of recombination exchanges related to different domain exchange events, we excised conserved blocks by picking the longest chains of 11 bp k-mers from event-wise parental alignments (Supplementary Text S1.8, Fig. S19a, Tab S33). A total of 91 candidate blocks were revealed (Tab S34, Fig. S19b-c), 37 of which were proximal to the breakpoints (Fig. S19b-c, Tab. S35), and were selected accordingly. The blocks corresponded to 111 sequences from parents. The filtered and chosen regions were rather homogenous in similarity length without statistical differences (Fig. S19d-e, Tab S36); however, for certain events, long regions up to 66 bp were located near the breakpoints (Tab S35). The mean length and similarity of the retained blocks reached 24 bp (15-66 bp) and 82.5% (67-98%), respectively (Tab. S35-36). We grouped these sequences using hierarchical clustering. According to the integral quality score, we obtained 10 flank clusters applying the Ward.D2 distance metrics (Supplementary Text S1.8, Fig. S20a-b, Tab. S37-38). The clusters comprised 6 to 22 sequences with 11 toxins on average (Tab. S38). To get a fuller picture, we disclosed the CD-HIT clusters with a 95% identity threshold for reference toxins and added all the representatives for each reference toxin, which did not distort the overall properties of the flank clusters (Fig. S20c-d; Tab S39-41). In the detected signals in Cry sequences, all 10 models formed from the final dataset of flanking sequences, except one, were equally distributed (Fig. S21a-c).

**S2.10. Domain exchanges are significantly connected with identity in the flanking regions**

To prove relationships between conserved regions in the domain sequences and recombination events, we compared domain-wise identities. We collected the pair-wise identities between toxins from the same events and other groups and found significant differences (*P* < 10e-16) between these groups (Fig. S22b-c). Upon closer analysis by classifying the toxins according to their role in the events, namely, parents, recombinants, and parent-child pairs (Tab. S43), we also found that similarity between flank regions is higher (*P* < 10e-16) in representatives from the same event, both total and cluster-specific (Fig. 3f, Fig. S22c-d). We then leveraged the logistic regression method based on the flank-wise similarity and participation of toxins in the same events. The model illustrated that the probability of engaging in recombination events directly (*P* < 10e-16) depends on the flank identities (Fig. S22e), indicating a tight connection between flanks and domain exchanges.

**S2.11. The available data on host specificity shows a skew in studying certain Cry types**

To assess the applicability of certain data sources of toxic activities, we collected host range data for 284, 184, and 148 proteins within the fourth and the third ranks, and strains, respectively (Fig. S23a). The number of individual assays predominantly included orders Lepidoptera, Diptera, and Coleoptera, accounting for 75-85% of the examples (Fig. S23b, Tab. S46). For most cases, proteins and strains exhibited activity against a single order, whereas there were two broad-host-range instances, such as Cry1Ba and the C18 strain related to four different orders (Fig. S23c, Tab. S47). A similar picture was obtained for species (Fig. S22d). However, three Cry toxin families, namely, Cry1Aa, Cry1Ab, and Cry1Ac, being the most studied, were toxic to a broad range of host species, ranging from 48 to 65 (Fig. S23d).

**S2.12. Host specificity of Cry toxins at the order level shows high dissimilarity, especially relative to minor parents**

To reveal global specificity changes after recombination events, we examined affected host orders for acceptors and donors of the domains. When examining host specificity changes, we focused on the data for toxins and strains, corresponding to 22 and 30 annotated events, respectively (Fig. S24a-j, Tab. S48-50). We observed distinct trends in host specificity change in Cry toxins attributed to different host groups. Anti-lepidopteran and nematocidal toxins were mostly unaltered. In contrast, those infecting hemipteran and dipteran pests showed a non-identical host range (Fig. S24a-j). The initial fraction of the events represented those with different orders affected by parents and recombinants, reaching 57-59%. After employing ancestral reconstruction, the number of different events dropped to 50-55% (Fig. 4a, Fig. S25a-j). A detailed analysis revealed that the unequal host range when comparing minor parents with recombinants exceeded 50%, whereas major parents were similar in 65% of cases (Fig. S26a-b, Tab. S51).

To reveal specific effects of recombination events on host range, we studied hosts on the species level. The affected species range was incongruent in almost all events, including ancestral states (Fig. S26a-b, Tab. S51). The distributions of difference ratios (the fraction of events with host specificity changes) diverged from random simulations except for minor parents at the order level of hosts after ancestral reconstruction (Fig. S26c, Tab. S52). The presence of host specificity switching was significantly associated (p<0.04) with the number of toxins (Fig. S27a-b, Tab. S53). Furthermore, the number of participants was significantly (*P* < 0.05) higher when comparing recombinants with minor parents when host changes occurred, and the pair-wise identity between toxins was lower as well (Fig. S27c-d, Tab. S54). The number of participants was significantly (*P* < 0.05) higher when comparing recombinants with minor parents when host changes occurred, and the pair-wise identity between toxins was lower (Fig. S27c-d, Tab. S54).

**S2.13. The similarity of the sets of affected hosts shows the primary role of major parents in delineating the activities of recombinants**

To reveal how domain exchanges contribute to the range of affected species and handle patchy data, we employed the Szymkiewicz-Simpson coefficient to reduce imbalance for certain Cry types (Fig. S28a-b, Tab. S55). According to the similarity distributions, major parents contributed more to the activities of recombinants (Fig. S29a, Tab. S56). At the same time, only for comparisons between minor parents and children, the coefficient was significantly higher (*P* < 0.05) in the events with an unaltered host orders range (Fig. S29b, Tab. S57). The observations were confirmed by linear models illustrating that minor parents of the third domain are generally more abundant and would likely exhibit unequal host specificity with recombinants (Fig. S29c, Tab. S58).

**S2.14. Recombination, in general, does not affect the absolute number of affected hosts in affected toxins**

To test the functional effect of recombination, we analyzed the net number of affected hosts in recombination-affected toxins. Generally, recombination events do not influence the absolute number of toxic activities (Fig. S30a-d, Tab. S59). The mean number of host orders in recombinants only in the group with unequal host specificity was slightly yet significantly (*P* < 0.05) higher in recombinants when compared with major parents, whereas the set of species remained unchanged (Fig. S31a-c, Tab. S59-62).

**S2.15. Major parents delineate the host specificity of children on a species level**

We summarized the distribution of intersections between hosts on the species level between parents and children in recombination events to find, which parents contribute more to functional activities. When analyzing species, 85% of cases were equal for comparisons of children with major parents, and only 38% of observations relative to minor parents were similar (Fig. S32c-d, Tab. S64). The distribution of affected species alterations when comparing minor parents and children resembled random simulations (Fig. S32e-f, Tab. S66).

**S2.16. Using the host specificity data for the third rank of Cry toxins is more accurate due to sequencing errors**

To carefully deal with summarizing Cry toxin content, we delineated on which level, according to the existing nomenclature, the toxins should be taken. Considering *cry* sequences in the fourth rank-wise rank led to overestimations sparked from sequencing errors, especially if *cry* genes of the strain were sequenced both by Sanger and NGS methods (Fig. S33a-b, Tab. S68). The extensive number of *cry* genes was reduced sufficiently if using the third rank, and we retained this level (Tab. S68).

**S2.17. Donors and acceptors of domains from multiple events co-occur in recombination-affected strains**

To understand if and how the presence of recombinants in the isolates shapes the composition of toxins subjected to recombination or unaffected ones, and also to characterize the patterns of co-occurrence of domain donors and acceptors, we classified assemblies and strains concerning the types of toxins they possessed (Tab. S68-69) and evaluated the inter-group intersections. We considered the datasets in two ways: (i) the strains/assemblies were attributed to toxin types (parents, recombinants, and the unsubjected ones) if they comprised at least one toxin of a certain type regardless of the other types; (ii) the strains/assemblies containing exclusively one type were considered (e.g., those including recombinants but not parents and/or non-chimeric toxins).

We found that parents and children from diverse events tend to co-occur in the same assembly/strain (Fig. S33c-d). Of those harboring only a single type of toxin, the ones containing parents prevailed (324 strains). The second largest group encompassed both parents and intact *cry* sequences (254 strains). According to median estimates of the content of Cry toxins, strains, and contigs were singletons, assemblies bore 3 *cry* genes, whereas plasmids and chromosomes bore 2 (Fig. S33e, Tab. S70). The observations reflect the absence of genome sequences for a plethora of strains, high fragmentation of assemblies, resulting in a lower number of *cry* loci related to contigs and strains, and a limited number of available *cry* sequences, implying that multiple potential events are left undetected.

In reference clusters obtained using CD-HIT with a 95%, 48.9% of toxins were affected by recombination (Fig. 2d). Unlike the data for reference toxins only, when summarizing which toxin types in a third rank-wise way are produced by the known natural isolates, we found that the fraction of recombination-affected toxins within the datasets reached 73% (Fig. 4b-c). Distinct recombination-specific groups prevailed in the datasets containing certain toxin types. More specifically, recombinants constituted 30%, parents – 79%, and unaffected by recombination toxins – 65% of the data within the respective subsets (Fig. S3f-h).

**S2.18. Genes encoding parents and children from the same events reside in close genomic regions**

We identified six events, with five of them displaying recombinants with one of the minor parents and one with both types of parents (Tab. S71). Toxin-encoding genes were located either close to each other, falling into GIs, or in distant regions. In all the cases, the *cry* genes described were flanked by insertion sequences, and several strains were generally enriched in MGEs (Fig. S34a-b). The strain HS18-1 was associated with two events with *cry* genes residing within two large pathogenic islands (Fig. S34a). Genetic loci coding for toxins Cry71Aa (recombinant) and Cry72Aa (minor parent) from event 8 were linked with the first island, as well as Cry30Ga (minor parent) from event 29 with the respective child (Cry50Ba) being adjacent to another minor parent (Cry30Ea). The second pathogenic island also housed a minor parent from event 109 (Cry69Ab). We also counted the overall number of MGEs in the genomes accordingly. Strains HS18-1, C15 (event 104), and MYBT18246 (event 73) were enriched with GIs, and the latter two were also rich in IS and exhibited nematocidal activities. The remaining assemblies were congruent with median values (Fig. S34b). The sequences of the domain donors and acceptors for all 8 events forming compact sister clades on the reference tree are relatively recent (Fig. S34c). The mentioned observations jointly illustrate that domain exchanges are associated with quick alterations in the toxin content of the strains, facilitating further propagation of hybrids in other strains.

**S2.19. Recombination is associated with an increase in the number of produced Cry toxins and strikingly alters their composition**

We found that bacterial strains in general are enriched with parents and children. To get a more detailed picture, we examined how recombination modifies the sets of *cry* genes. We picked all strains or assemblies in which parents and children resided. We considered all toxins produced by these strains and assessed the similarity between sets when comparing children with parents or different parents with each other. Almost 70% of the events for both analyses (34 and 35) lacked any overlaps (Fig. 4d, Fig. S35a-b, Tab. S72). The presence of parents and especially recombinants is significantly (*P* < 0.05) associated with a greater number of toxins, in contrast to the unsubjected isolates reaching median values of 4.5, 4, and 3, respectively (Fig. 4e, Tab. S73). However, in strains, only representatives including recombinants bore 3 toxins vs. one and two, respectively (Fig. 4e). If scrutinizing strains containing recombinants, parents, or intact toxins exclusively without other types, the presence of parents is associated with more Cry toxins as well (Fig. S35c).

**S2.20. The distribution of major parents in bacterial strains implies the intensive selection of functional recombinants**

To characterize the effect of recombination events on bacterial isolates, we compared strains containing toxins belonging to different domain exchange events. In terms of affected host orders, recombination-affected strains significantly (*P* < 0.05) outperform non-subjected representatives (Fig. S36a, Tab. S73-75). More specifically, isolates bearing major parents exhibit more activities than other groups (Fig. S36b, Tab. S76), which is more visible when specificity changes occur (Fig. S36c, Tab. S77). In contrast, the number of toxins is higher in strains with recombinants, especially when the host range is altered (Fig. S36d-e, Tab. S78-79). In strains producing recombinants, 75% of representatives harbored *cry* loci with the same activities as in major parents. In contrast, the reverse was true for those containing major parents relative to recombinants (Fig. S37a-c, Tab. S80-81). This can be explained by the prevalence of major parents in the isolates, which sporadically engage in domain exchanges, forming less frequent chimeras probably due to the eradication of ineffective moieties (Fig. S38a-h, Tab. S82-83). The conservation of host orders was associated with similarity in spectra of affected species and produced toxins (Fig. S38e-h).

**S2.21. Individual properties of recombination-affected strains and event-wise comparisons imply narrowing the host range with a sporadic increase in affected hosts**

We studied the links between distinct features of the strains housing *cry* loci participating in recombination as parents and children (Tab. S82-84). Predictably, the more toxins they produce, the broader the host range is (Fig. S39a), except for the strains producing minor parents with different affected orders compared to recombinants (Fig. S39a). This observation could be explained by the fact of multiple minor parents per single event and hidden ancestral exchanges. If descents of recombination events would concentrate in the strains containing minor parents, one would expect the accumulation of toxins and an increase in host range, whereas the absence of these relationships supports the free exchange of domains from minor parents. The similarity between host species in children and parents was directly connected (*P* < 0.05) with the overall set of produced toxins in the isolates encompassing them, indicating that exchanges occur within the same ecological niche (Fig. S39b-c). The number of affected hosts and toxins in the isolates carrying minor parents was inversely proportional (*P* < 0.05 according to the linear model) to the respective values in those producing recombinants. This reflects consequent events because the presence of recombinants coincides with the strain potency to recombine and collect chimeric toxins, whereas the strains holding minor parents are conserved (Fig. S39d-40b, Tab. S85). Additionally, several recombination-affected isolates produce more toxins and affect a wider host order range compared with parental strains. However, most of the cases, contrarily, imply a narrowed host range after recombination (Fig. S40c).

**S2.22. Larger connected components of the strain graph are enriched with toxins, affected hosts, and recombination events**

To identify global population parameters of Cry-producing isolates, we analyzed the strain graph, uniting isolates with overlapping toxin content. In the strain graph, 114 of the connected components were singletons (Fig. S42a). The largest component included 556 strains, 23 – 4 to 36 strains, and the rest – 2-3 strains (Tab. S86). The number of components possessing participants in recombination events was 73 (Tab. S87), and the largest one incorporated 16 events. Significant dependence (*P* < 0.01) between the component size and the number of events was revealed with the linear regression model) (Fig. S42b). The components devoid of recombination signals housed significantly fewer toxins (*P* < 0.01 according to the Wilcox test) than those associated with domain exchanges (Fig. S42d-f). The components were mostly linked with only a single host order related to toxins in them. Nevertheless, there were examples of generalist groups with plenty of strains (Fig. S41, Fig. S43a). The proportion of generalist strains was slightly higher in components with toxins undergone recombination (Fig. S43a). Moreover, this group comprised more components with at least one known host (Tab. S86), namely 63% vs. 50% (p<0.02 according to Fisher’s exact test).

We analyzed the toxin content of connected components in the strain graph into which parents and children fall using the Jaccard coefficient. A strikingly high inconsistency between the sets of toxins produced by strains within connected components containing recombinants and parents was found. More specifically, for more than 50% of the events (26-31), no intersections were reported (Tab. S88).

**S2.23. Toxins of the Cry32 and Cry7 families are prospective biopesticides with multiple pesticidal activities**

Among the widespread Cry families in the strain graph, members of the abundant Cry32 class were reported to be toxic against Lepidoptera, Diptera, and Nematoda, and they belonged to 5 recombination events, implying that this Cry group is highly promising and deserving of further investigations. Another prospective Cry family is Cry7, forming a distinct connected component in a host-wise strain graph (Fig. S41). Although the host spectrum of these toxins remains unknown, they were reported to have certain domains, Cry7Ca1, the only known protein to act against *Locusta migratoria manilensis* Orthoptera [50]. Therefore, the Cry7 family on the whole deserves testing for anti-orthopteran activity as a prominent source of possible biocontrol agents.

**S2.24. Domain exchanges in connected components link specialists and generalists, reflecting the process of specialization**

To reveal the population-wise effect of domain exchanges, we scrutinized a combined graph of strains producing shared sets of Cry toxins. The distributions of order-wise toxicity for particular events indicate that certain toxins fell into both specialist and generalist categories, which was most evident for anti-dipteran and anti-lepidopteran strains (Fig. S43b). We calculated the similarity between the hosts for toxins/strains within the components harboring parents and children. Using the Szymkiewicz-Simpson coefficient, we demonstrated that the components overlap sufficiently. At the same time, the Jaccard coefficient was generally lower (Fig. S43c-d, Tab. S88). Because the Jaccard coefficient reflects whether one set of activities could be a direct subset of the other, whereas the overlap coefficient reflects the fact that two sets have at least one item in the intersection, the similarity possibly reflect the exchanges between generalists and specialists. To prove our suggestions, we combined the strain graph with recombination events and linked the connected components by edges attributed to domain transfers (Fig. S44a-b). In the graph, larger connected components with an extensive host range contained more edges linking them with smaller components with specialized strains.

**S2.25. The filtration of the events reduces the foreground ω estimates** **and does not affect the background**

To get significant evolutionary selection signals using branch models, we applied the LRT (likelihood ratio test). The two-step filtration of the events based on the LRT significantly changed the mean ω for the foreground branches, reaching 0.52, 0.71, and 0.16 for all events, non-neutral yet identical within branches, and with unequal rates in the foreground branches (Fig. S52b, Tab. S101), being associated with the number of marked toxins (Fig. S52c-d). This observation fits with a pronounced negative selection rate with the preservation of the acquired domains. At the same time, the mean ω for the background remained unchanged and hovered around 0.367. The foreground ω within the threes built on diverse domain sequences exhibited no significant differences (Fig. 5b, Fig. S52e).

**S2.26. The patterns of evolutionary selection signals in parents and children tend to differ due to ancestral exchanges**

To analyze detailed patterns of evolutionary selection, we compared results for each domain of parents and children. Using domain-based trees gained patterns absent in the respective data for parents (e.g., when the purifying selection in recombinants affected the third domain in recombinants and the first domain in parents). We reported such inconsistencies in 13 events, especially if the first domain was transferred (Fig. 5a, Fig. S52a, Tab. S101). Two instances subjected to positive selection were presented by chimeric events (103 and 45) in which the recombinant obtained domains I and II from distinct parents, with an unknown source of the third domain (Supplementary Text S2.6, Fig. S14).

**S2.27. Site models associated with positive selection detection are prevalent in the dataset**

To identify which parts of the exchanged domains are subjected to evolutionary selection, we applied site models. A total of 228 significant signals with one M1, 69 – M3, and 157 – M8 optimal site models were chosen, corresponding to relaxation and positive selection for the latter two models, respectively (Tab. S103). For event 95 associated with the M1 model, the enrichment with relaxed sites was reported. For most cases belonging to the M3 model, relaxed sites were located on the boundaries between the domains opposite from the ones transferred from the minor parents (Fig. 5c). The highest portion of evenly distributed positively selected sites was found in all linked events, (i.e., those possessing parents from three distinct sources (Fig. S14)), whilst in other events, the sites were concentrated in the center of the domains (Fig. 5c). The total number of positively selected and conserved sites reached 369 and 259; thus, a total of 59% of sites were subjected to positive selection (Fig. S53a, Tab. S103). We revealed that the exchanges of the first and the third domains are associated with a higher fraction of conserved sites in domain II (Fig. S53b-c). Nevertheless, we have not found significant differences between the total number of positively selected and conserved sites in the domains reflecting individual but not average patterns in the particular events (Fig. S53d-e).

To figure out the factors delineating the number of positively selected sites, we compared the respective values with the number of toxins and the ω, both background and foreground, in trees reconstructed to calculate branch models for parents and recombinants. Neither the total number of toxins nor the marked ones has shown any impact on the number of sites, possibly indicating that obtaining a tree for revealing selection on sites did not influence the assessment procedure (Fig. S54a-b, Tab. S103). The foreground ω, both when considering all signals and those with significant selection signals, has no relationship with the number of sites (Fig. S54c-d), whereas a positive correlation with the background ω was detected (Fig. S54e-f). Given that the length of the constructed clade did not affect the number of sites and the ω estimates, we might conclude that choosing the tree does not disturb the selection estimates.

**S2.28. Branch-site models report numerous signals of negative selection reflecting the conservation of efficient domains**

To obtain a detailed picture of the evolutionary selection effect, we combined the distribution of parents and children on phylogenetic trees and characteristics of their sequences by using branch-site models. Branch-site analysis tends to inflate the number of positive selection signals for some events, marking the whole domain instead of providing particular sites (Fig. S55a-b). The events with sporadic relaxation sites were instead enriched with conserved regions, except for event 95, which had prolonged relaxation regions and turned to a positive selection signal for the whole domain (Fig. S55a-b). We compared the proportion of positively selected and conserved sites per domain in the parents and children, omitting inferences with the over-estimation. A significantly higher percentage of positively selected sites (*P* < 0.01) was found in the recombinants within domains I and III when considering the transfer of the first domain, as well as domain II of recombinants and domain III of parents for the events leading to the swapping of the third domain (Fig. 5d, Fig. S55c-d). Neither the amount of positively selected nor conserved sites was related to the number of all and marked toxins, which can be perceived as a further indication that the instances calculated reflect the evolutionary dynamics rather than the properties of subtrees chosen (Fig. S56a-b). Linear models also exhibited that the respective abundance of both site types was significantly and positively associated with the background ω (Fig. S56c-d). Therefore, the properties of the trees were not related to the number of sites, further proving the validity of selection estimates. On the whole, conserved sites prevailed; however, a significantly higher percentage of positively selected sites (*P* < 0.01) was found in the recombinants within domains I and III when considering the transfer of the first domain as well as domain II of recombinants and domain III of parents for the events leading to the swapping of the third domain (Fig. 5d, Fig. S55c-d).

**S2.29. Evolutionary selection does not reflect changes in affected orders**

To reveal the relationships between the functional consequences of domain exchanges and the strength of evolutionary selection, we compared sets of events with or without changes in affected host orders. We considered whether the model parameters evaluated contribute to unequal sets of host orders between recombinants and parents (Tab. S102). The inferred distributions provided some evidence that the higher ω and number of positive sites were associated with differences in host sets. In contrast, the events with the same affected orders were enriched with conserved sites. However, the amount of data was not enough to gain significance except for the positively selected sites according to site models only (Fig. S57a-e).

**Supporting Figures**

**Fig. S1**. Schemes of methodological approaches used in the study. (**a**) Filtering recombination events regarding the position of parents and recombinants on the phylogenetic tree. The analysis is performed for each domain-based tree independently, and the parent is determined by the type of transferred domain. In the first step, all nodes whose bootstrap supporting values are less than a predefined threshold of 70 are removed. We unified leaves with recombinants and parents in single branches by resolving polytomy. In case toxins missed by RPD4 predictions were found in the clades, the toxins were interpreted as parents of the individual domain according to the domain-wise tree. If the branches involving parents and recombinants were found in the root of the tree, their phylogeny was considered incongruent, and the events were filtered out accordingly. Letters “r”, “p”, and “u” denote recombinants, parents, and unaffected toxins, respectively. (**b**) Data preparation scheme for evolutionary selection analysis. Domain-wise phylogenetic trees corrected for recombination, as previously described, were used to extract subtrees including the least common ancestor within the event. During the procedure, we disclosed clusters obtained with CD-HIT software. We then iteratively moved to upper branches and retained subtrees if their length exceeded the threshold defined as the number of recombination participants multiplied by two to get the background. If the procedure satisfied the threshold, gained subtrees with more than 50 leaves, we screened all the subtrees with disclosed clusters arranged by the identity with the domain sequence of recombinants, and iteratively added tree nodes until the required threshold was reached. The sequences in the trees were then aligned with MAFFT. Evolutionary models were selected using Modeltest-NG, and phylogenetic trees were reconstructed with RAxML-NG. The evolutionary selection was analyzed based on the alignments and guiding trees using the ETE3 package. (**c**) Shown are the evolutionary models tested. Data prepared during the previous steps were analyzed for each event separately. The best-fit models were chosen according to the LRT (likelihood ratio test). Unlike site models, when examining branch and branch-site models, either recombinants or parents were marked as foreground branches and evaluated separately, which are displayed as apostrophes on the scheme.

**Fig. S2**. Properties of Cry toxin sequences identified in four publicly available datasets, namely, NCBI Assembly [51], GenBank, IPG (identical protein groups), and the BPPRC (Bacterial Pesticidal Protein Resource Center) [4]. (**a**) The total frequency of domain-wise similarity between all Cry toxin sequences used in the study. The color code depicts the type of the dataset, namely, all toxins and reference clusters obtained using CD-HIT with a 95% identity threshold. (**b**) Distribution of the domain lengths in the analyzed dataset. (**c**) Per-site conservation scores in the sequences of the domains calculated as the maximum frequency of non-gap symbols in the alignment of the particular domain. The first domain has a higher conservation score with a mean of 0.23 in comparison with the second and third domains (0.16). The number of highly conserved sites with conservation scores exceeding 0.85 reached 16, and 41 conserved sites for the first, second, and third domains, respectively. (**d**) The dependence between the length of the domain and mean pair-wise identity for the respective toxin with all toxins from the dataset. The color corresponds to the species attributions, whereas the shape encodes the domain type.

**Fig. S3**. Characteristics of CD-HIT clusters of Cry toxins obtained using a 95% identity threshold. (**a**) Frequency of clusters according to the number of toxins within them. (**b**) The mean pairwise similarity of the domain sequences within all CD-HIT clusters obtained using a 95% identity threshold. (**c**) The same distribution of domain-wise identities in clusters containing more than 10 toxins. (**d**) Dependence between the abundance of the cluster and the mean domain-wise identity between toxins within the cluster. The shape of points denotes the domain. The size encodes the number of toxins. (**e**) The distribution of pairwise identities of the domains within clusters encompassing more than 10 toxins. Codon-wise mismatches between the sequences of the domains within clusters with more than 10 toxins. The left panel shows the coordinates of mismatches colored according to their type (synonymous, non-synonymous, and indels) with a green dashed line designating the boundaries between the domains. The middle panel summarizes the number of such mismatches per cluster, and the right panel denotes the abundance of the cluster. (**f**) The total number of substitutions identified in the particular domain. (**g**) The normalized non-synonymous ratio for the particular domain calculated as the sum of non-synonymous substitutions and indels divided by the number of synonymous mismatches, multiplied by the length of the cluster-wise domain-based alignment and the number of toxins within the cluster. Asterisks denote statistical significance using the Wilcox test when comparing the respective values of domain sequences.

**Fig. S4**. Properties of Cry sequences of the analyzed dataset concerning species in which toxins were identified and the identity with the respective known homologs. (**a**) Distribution of species attributions of the Cry toxins dataset. Shown are the data for all toxins and reference clusters obtained using CD-HIT with a 95% identity threshold. Upper plots display the absolute numbers, The lower plots depict square root-adjusted values for better details. A total of 30 toxins were attributed to other species, including *Bacillus mycoides*, *B. cereus*, *B. wiedmannii*, and other not-*Bt* species, or pertained to different genera, such as *Paraclostridium bifermentans* and *Clostridium botulinum*. (**b**) The relationship between the length of the domains and the identity with the respective homolog from the *Bt* nomenclature for all toxins and only novel ones. The color corresponds to the species attributions. The shape encodes the domain type.

**Fig. S5**. Phylogenetic inferences based on the sequences of the domains in the analyzed dataset. The upper phylogenies correspond to the reconstructed ML trees (maximum likelihood) obtained from the alignments of the first (**a**), second (**b**), and third (**c**) domains. Color code denotes the type of toxins, with the red color corresponding to known toxins deposited in the *Bt* nomenclature and the blue color designating novel proteins. The lower figures depict tanglegrams demonstrating topological differences of the reference ML phylogeny based on the concatenated alignments of the domains with partitioned evolutionary models, with the respective trees of the first (**d**), second (**e**), and third domains (**f**). The optimal evolutionary model for the third domain differed from the other two domains (HKY+G4 vs. TVM+G4).

**Fig. S6**. Heatmap with the pair-wise identity of sequences of the first (**a**), second (**b**), and third (**c**) domains. The order of rows and columns corresponds to the order in the reference ML phylogeny based on the concatenated alignments of the domains with a partitioned evolutionary model. Arrows indicate the zones in which local identity differs from the expected order.

**Fig. S7**. Recombination events reported by RDP4 launched on MAFFT-aligned nucleotide sequences of Cry toxins. (**a**) The total number of raw events identified with RDP4. (**b**) The number of events after discarding partial exchanges, i.e., affecting less than 70% of the domain lengths. (**c**) Types of events in the final dataset with filtering based on tree congruence. The color corresponds to the transferred domain. (**d**) Distribution of breakpoint coordinates for recombination events, as well as those devoid of partial exchanges (**e**), and filtered based on tree congruence (**f**). On the X-axis, the relative coordinate in the sequence is presented. Plotted on the Y-axis is the mean *P* value from all recombination detection tests included in the RPD4 software. Grey dotted lines represent the boundaries of the domains. For a detailed description of the filtration process, see Fig. S1a.

**Fig. S8**. Properties of recombination events detected with RDP4 launched on MAFFT-aligned nucleotide sequences of Cry toxins. Three datasets are considered: “Raw” index encodes all events detected by RDP4, “Filt_partials” denotes the set of events affecting more than 70% of the respective domain, whereas “Filt_tree” marks the final dataset with filtering based on tree congruence. For a detailed description of the filtration process, see Fig. S1a. (**a**) Event-wise characteristic of recombination exchanges within three datasets. Each point represents the set of toxins colored according to their type in the event. i.e., recombinants, and parents. Parents are marked as major or minor, with the latter providing two domains, and the former transferring one domain. The size of the point is proportional to the number of toxins. The first adjacent line encodes the type of the event (the transferred domain). The second line provides information about whether the parent is unknown, i.e., absent in the analyzed dataset. (**b**) Distribution of the number of toxins within the events. The color of the bar represents the type of recombination participants. Plotted on the X-axis is the number of toxins, whereas the Y-axis denotes the number of events. In the final dataset, 45 events included one recombinant per event, whereas 4 events encompassed two toxins, and 1 event comprised 5 participants. The same goes for parents. Of all events, 31 were those with a single minor and major parent, 13 possessed either two or one major or minor parent (with 3 parents in total), and the rest of the events (6) were related to a greater number of parents. (**c**) The number of unknown parents within the recombination events. The total number of events grouped according to the transferred domain is presented. Color encodes the type of the parent (minor/major). The reduction in the number of events with unknown parents was from 46 (38%) to 8 (16%). In the final set, 5 major and 3 minor unknown parents remained.

**Fig. S9**. Distribution of mean sequence identity and mismatch rates of recombination events detected with RDP4 launched on MAFFT-aligned nucleotide sequences of Cry toxins. Three datasets are considered: “Raw” index encodes all events detected by RDP4, “Filt_partials” denotes the set of events affecting more than 70% of the respective domain, whereas “Filt_tree” marks the final dataset with filtering based on tree congruence. For a detailed description of the filtration process, see Fig. S1a. All the measurements refer to three groups of recombination events according to the swapped domain. (**a**) Average domain-wise nucleotide identity between the sequences of recombinants and parents. Asterisks denote statistical significance using the Wilcox test when comparing the respective values of major and minor parents. The mean similarity between the transferred domain of recombinants and minor parents reached 80%, 66%, and 71% for events in which the first, the second, and the third domains were swapped, respectively. (**b**) The dataset-wise mean identity difference between the transferred and non-transferred domains of major and minor parents compared with the domain sequences of recombinant toxins. Asterisks denote statistical significance using the Wilcox test when comparing three sets of recombination events. Error bars represent mean square error. Difference estimates reached 19%, 12%, and 16% when considering minor parents, and 6%, 5%, and 16% concerning majors for events with the first, second, and third domains, respectively.

**Fig. S10**. Properties of the final dataset of recombination events detected with RDP4 launched on MAFFT-aligned nucleotide sequences of Cry toxins. For a detailed description of the filtration process, see Fig. S1a. (**a**) Event-wise characteristic of recombination exchanges. Each point represents the set of toxins colored according to their type in the event. i.e., recombinants, and parents. Parents are marked as major or minor, with the latter providing two domains, and the former transferring one domain. Parents identified when considering domain-wise trees for each domain are also shown. The size of the point is proportional to the number of toxins. The first adjacent line encodes the type of the event (the transferred domain), whereas the second line provides information about whether the parent is unknown, i.e., absent in the analyzed dataset. (**b**) The effect of tree-wise filtration on the number of parents within recombination events. Plotted on the X-axis is the number of toxins reported by RDP. The Y-axis represents the number of parents after filtration. The shape and the color of the points correspond to the type of parents. A total of 35 events remained unchanged, whereas for 8 events the number of majors increased, and for 7 events the number of minors was reduced. (**c**). Domain-wise number of mismatches between parents and recombinants normalized by the corresponding alignment. Mismatches were calculated only for transferred domains within the final filtered set of events. Asterisks denote statistical significance using the Wilcox test between domain-wise mismatch rates.

**Fig. S11**. Distribution of regions affected by recombination using ClonalFrameML (**a**), Gubbings (**b**), and fastGEAR (**c**). The order of rows corresponds to the reference phylogeny, and the positions refer to the MAFFT-generated alignment of processed toxins (regions from the beginning of the first and the end of the third domain). Putative exchanged areas are marked with blue and red colors.

**Fig. S12**. The graph of domain exchanges between Cry toxins. The color of the node corresponds to the role of the toxin in recombination events, i.e., recombinants, parents, and those participating in multiple swaps, thus being parents and recombinants simultaneously. Edge colors denote the domain transferred, namely, one domain for minor parents and two domains for major parents, respectively. Black edges connect toxins if a particular event contains multiple recombinants.

**Fig. S13**. Properties of weakly connected components of the recombination graph. (**a**) The number of recombination events within each connected component colorized according to the types assigned regarding the transferred domains. (**b**) The number of toxins (nodes) within each connected component. The color corresponds to the role of the toxin in recombination events, i.e., recombinants, parents, and those participating in multiple swaps, thus being parents and recombinants simultaneously. (**c**) The total number of graph edges within all connected components. Colors denote the domain transferred, namely, one domain for minor parents and two domains for major parents, respectively. Black edges connect toxins if a particular event contains multiple recombinants following the distribution of event types; most of the edges refer to the third (161) and the first (41) domains, as well as those presenting links from major parents amidst events involving the third domain with the first and the second domain transferred together (42).

**Fig. S14**. Schemes of domain exchanges of linked recombination events, namely, those affecting the same recombinant. Shown are the initial parents reported by RDP4 and the corrected sets of parents for such events taken from the recombination graph. The color corresponds to the role of the toxin in recombination events (i.e., recombinants, parents), and unknown parents are shown as grey circles (unknowns are omitted in Fig. S11). The colors of the edges denote the domain transferred, namely, one domain for minor parents and two domains for major parents, respectively. In total, there were 3 pairs of events affecting 3 toxins: Cry8Pa1, Cry52Ca1, and Cry40Da1_65.0_CAH33957.1, with the latter demonstrating 65% similarity with the closest reference from the BPPRC (Bacterial Pesticidal Protein Resource Center) database (see Tab. S24).

**Fig. S15**. Genetic diversity and recombination intensity of *Bt* assemblies bearing Cry toxins or lacking them. (**a**) The number of gene groups (core, soft core, shell, and cloud) within pangenomes reconstructed using Panaroo in all publicly available *Bt* assemblies, as well as those containing Cry toxins (**b**), and participants of recombination events (**c**). (**d**) Regions susceptible to homologous recombination among all *Bt* genomes analyzed, assemblies bearing Cry toxins (**e**), and those harboring recombinants and parents involved in the domain exchanges (**f**). Recombination events detected with ClonalFrameML are marked with a blue color and placed among concatenated core gene alignments obtained by Panaroo. Adjacent phylogenetic trees represent ClonalFrameML-corrected guiding trees reconstructed with FastTree from the core gene alignments.

**Fig. S16**. The relation between mobile genetic elements (MGEs) and recombination events. The colors of points and box plots correspond to the sets of *Bt* assemblies, namely, all genomes (“all”), assemblies bearing Cry toxins (“cry”), and those incorporating participants in domain exchanges (“rec”). (**a**) Dependence between the total number of insertions (IS) and genetic islands (GIs), prophages and genetic islands (**b**), insertions and prophages (**c**) within the aforementioned sets of genomes. (**d**) Statistical comparison between the average number of certain MGEs in the *Bt* assemblies described above. Asterisks denote significant differences according to the Wilcox test with *P* values adjusted using the Benjamini-Hochberg procedure. (**e**) The percentage of Cry toxins associated with the regions of IS and GIs. Shown are all Cry toxins and recombinants/parents involved in the domain exchanges

**Fig. S17**. Determination of optimal clusterization approach for event-wise grouping of regions flanking breakpoints. The distance matrix used was based on integral estimates of per-coordinate identities between the sequences of major and minor parents within 100 bp regions surrounding the breakpoints. Three distance metrics were applied on the respective coordinate-wise estimated, namely Euclidean distance, Manhattan distance (the sum of the absolute distances), and cosine distance. The estimates are marked with prefixes “eucl”, “sub”, and “cos”, respectively. These methods were also used on the moving average of the identity values within the vectors to compare with a period of 7 bp, which is delineated with the “roll” postfix. (**a**) Plotted is the most optimal number of clusters when using particular metrics according to the highest silhouette score calculated for the flanks located between the first and the second domain, and between the second and the third domains (**b**). The shape of the points encodes the clustering algorithm, namely, hierarchical clustering and k-means. Arrows indicate the best metric chosen for further analysis. (**c**) The distribution of silhouette scores for a diverse number of clusters within the best method for the first and the second flanks (**d**). (**e**) Dendrogram visualizing hierarchical clustering patterns for the first and the second flanks (**f**). The numbers correspond to recombination events. The Grey dashed line indicates the threshold for cluster delineation.

**Fig. S18**. Mean identity within regions surrounding recombination breakpoints. (**a**) Coordinate-wise mean identity between parental sequences in recombination events within two regions, namely between the second and the third domains and between the first and the second domains (**b**). Identity is calculated for 100 bp flanking regions before and after the respective breakpoints. Dashed lines indicate the location of breakpoints. Grey areas represent the standard error. The data are grouped according to the clusters of events obtained using the most optimal clustering pipeline and algorithm, namely hierarchical clustering based on the cosine distance metrics applied on the rolling average of the vectors with coordinate-wise identities. (**c**) The comparison between the mean identity of peaks within the event-wise clusters of flanking regions and sequences of the whole domains, either those that were transferred from the minor parent to the respective recombinant, and the adjacent non-transferred domains. Shown are comparisons for regions surrounding the first and the second domains (“Flank 1”) as well as the second and the third domains (“Flank 2”), respectively. Asterisks display significant differences according to the Wilcox test with *P* values followed by the Benjamini-Hochberg correction.

**Fig. S19**. The search for the compact conserved regions using a k-mer-based approach. (**a**) Mean identity estimates for the k-mers when comparing the sets of minor and major parents in recombination events. The dot represents a k-mer with a length of 11 bp. Plotted on the Y-axis is the mean coordinate-wise identity. The Y-axis depicts the position of the k-mer in the regions surrounding the breakpoints (100 bp before and after). Here and in other figures, the breakpoint is marked with the green vertical dashed line. The horizontal grey dashed line indicates the identity threshold used to infer the conserved regions presented by the consecutive k-mers with the identity estimate above the shown threshold. The threshold is calculated as the median, summed with the general deviation of the mean identity values. The bold numbers represent the identity of recombination events. Here, only four representatives are shown. The full list of the k-mer chains is presented in Tab. S33. The plots for all the events are available at the GitHub repository (https://github.com/lab7arriam/Cry_phylogeny). (**b**) The conserved regions formed as the consecutive chains of k-mers satisfying the above-described identity threshold for 100 bp breakpoint-flanking regions between the first and the second, and between the second and the third domains (**c**), respectively. The dots display the beginning and end coordinates relative to the breakpoints, with the color being proportional to the identity between parental sequences within the regions. The plots are split into two parts, namely, all regions satisfying the determined threshold (“All flanks”) and those closest to the breakpoints (“Closest flanks”). (**d**) The comparison between the chosen flanks proximal to the breakpoints and other conserved regions in terms of coordinate-wise sequence identity estimates and the lengths (**e**) of the flanks, respectively.

**Fig. S20**. The process of picking the most appropriate clustering method for uniting similar conserved regions presented in Fig. S18b-c. (**a**) The distribution of quality metrics for obtaining the clusters to reconstruct consensus models. Plotted on the Y-axis is the integral quality score described in Supplementary Text S2.8. On the X-axis, the method for the hierarchical clustering procedure is presented. The shape of the points reflects the aligner (BioPython pairwise alignment suite and MAFFT) for calculating the metrics for preparing the distance metrics. The size is proportional to the total number of clusters. Finally, the color denotes the means for calculating the similarity between flank sequences. The first word from the pairs indicates the alignment mode (Global or Local), whereas the second one designates the way for evaluating the similarity (i.e., if specified as global, the number of matches is divided by the length of the alignment), whereas the local method implies that the denominator is the length of the shortest sequence. The chosen method is marked with the black arrow. More specifically, using MAFFT in the local mode with subsequent global-based identity evaluation and clustering based on the “Ward.D2” distance with a cutoff of 10 clusters was applied. (**b**) Visualized distance matrix between the flank sequences arranged according to the best method and the mode for obtaining the similarity score. The color of the tiles is proportional to the pairwise sequence identity. (**c**) The impact of adding the members of the reference clusters from the CD-HIT clusters based on the 95% identity threshold on the pair-wise similarity and flank lengths (**d**) in the clusters encompassing conserved regions.

**Fig. S21**. The summary of consensus-wise hits with conserved flank-based models using the utility GLAM2Scan within the dataset of all deduplicated Cry toxins. (**a**) The distribution of hits per toxin, colored according to the consensus model, represents the cluster of conserved blocks displayed relative to all toxins and presented in a certain cluster only (**b**). Grey dotted lines correspond to the borders between the domains. (**c**) The total number of hits attributed to flank clusters within the dataset of all toxins and those participating in domain-swapping events as donors and/or acceptors.

**Fig. S22**. Relationships between the presence of signals representing the consensus models of conserved flank clusters and recombination events. (**a**) The distribution of flank-wise hits revealed with the GLAM2Scan software classified according to the domain in which they are located. The presented pie charts illustrate the hits for all toxins and participants of recombination events. (**b**) Comparisons between global pair-wise identity between toxins from the same recombination events, irrespective of their role (“RA”) and other toxins (“U”). The leftmost plot refers to all similarity estimates, whereas the biplots on the right are grouped according to individual clusters. Here and below, asterisks denote significant differences according to the Wilcox test with *P* values adjusted using the Benjamini-Hochberg procedure. In the case of within-cluster comparisons, certain clusters are divided with grey dashed lines. (**c**) Similarity value between toxin pairs regarding their role in recombination. The groups are as follows: recombinants (“R”), parents (“P”), children compared to the parents (“RP”), and other toxin pairs, unaffected or not from the same events (“U”).

**Fig. S23**. Overall distribution of activities of studied Cry toxins and strains encompassing them. (**a**) The total number of instances having at least one known host to which the activity was shown. Three groups are considered, namely proteins within the fourth (100% identity) and the third rank (95% identity within the toxin group), and strains. (**b**) The proportion of individual bioassays made for toxins and strains. The colored within-bars fraction corresponds to the number of unique species attributed to higher taxonomical categories. (**c**) The frequency of strains and proteins in the context of the amount of known affected host orders and species (**d**).

**Fig. S24**. The properties of parents and recombinants in terms of the hosts attributed to them. (**a**) The percentage of different orders against which recombinants and parents are active. The data on the activity are based on the third rank of the toxins. The portion of particular orders within the pie charts is color-coded. The left adjacent bar corresponds to the domain transferred in the particular event. (**b**). The number of toxins in the event (recombinants and the parents, either major or minor ones). “Rec” indicates recombinants, “Min” – minor, and “Maj” – major parents, respectively. The size of the dots is proportional to the number of participants, whereas the intensity of the color delineates the mean pair-wise identity between the transferred domains. (**c**). The classification of the events according to the affected orders. The events are called different in terms of host specificity if they are not completely equal, and the same otherwise. The data are encoded by the color of the tiles. Shown are comparisons between recombinants and minor/major parents and the ultimate classification (“Glob”). The whole event is marked as different if, at least for one child-parent pair, different host ranges were reported. Figures (**d**) and (**e**) represent similar information as is presented in (**a**) and (**c**), but the proportion of the hosts is calculated according to the species that are attributed to the orders. The difference in affected hosts is evaluated using the species-wise data. Figures (**f-g**) describe the same data as in the aforementioned figures using an extended source for the host range. The annotations from the strains are taken if the respective protein-wise host spectrum is unknown.

**Fig. S25**. The properties of parents and recombinants in terms of host specificity regarding the ancestral reconstruction of the affected species/orders. (**a**) The percentage of different orders against which recombinants and parents are active after the ancestral state reconstruction procedure. The data on the activity are based on the third rank of the toxins. The portion of particular orders within the pie charts is color-coded. The left adjacent bar corresponds to the domain transferred in the particular event. (**b**). The number of toxins in the event (recombinants and the parents, either major or minor ones). “Rec” indicates recombinants, “Min” – minor, and “Maj” – major parents, respectively. The size of the dots is proportional to the number of participants, whereas the intensity of the color delineates the mean pair-wise identity between the transferred domains. (**c**). The classification of the events according to the affected orders. The events are called different in terms of host specificity if they are not completely equal, and the same otherwise. The data are encoded by the color of the tiles. Shown are comparisons between recombinants and minor/major parents and the ultimate classification (“Glob”). The whole event is marked as different if, at least for one child-parent pair, different host ranges were reported. Figures (**d**) and (**e**) represent similar information as is presented in (**a**) and (**c**), but the proportion of the hosts is calculated according to the species that are attributed to the orders. The differences in affected hosts is evaluated using the species-wise data. Figures (**f-g**) describe the same data as in the aforementioned figures using an extended source for the host range. The annotations from the strains are taken if the respective protein-wise host spectrum is unknown.

**Fig. S26**. The distribution of recombination events regarding the changes in affected host orders and species. (**a**). The number of events classified according to alteration in host range in recombinants relative to minor and major parents. The parent-child comparisons were considered different if the affected set was not completely identical. Both species and orders were included. Certain datasets were used depending on the data source (toxins only – “Toxins” or the spectrum complemented with the information available for strains – “With strains”) and the ancestral reconstruction procedure (the data per se, “Unchanged”, or the ancestral state, “Ancestral”, was studied accordingly). (**b**) .The percentage of the recombination events attributed to changes in affected hosts. (**c**). The distribution of bootstrap-like sampling of the events from the aforementioned datasets with 1000 iterations. The estimates represent the percentage of the items marked as different. The plots are colored according to the origin of the data, i.e., random simulated samples and bootstrap subsamples from the dataset.

**Fig. S27**. Descriptive comparisons between Cry toxin nucleotide sequences attributed to the events according to changes in the range of affected orders. The parent-child pairs are considered different if at least one host was unequal between recombinants and parents. Here, major and minor parents are compared with recombinants independently. Two data sources for the host range were considered, namely: the data for toxins only (“Toxins”) or the extended dataset with the known spectrum of strains (“With strains”). Both the available host spectrum for toxins/strains (“Unchanged”) and the reconstructed ancestral state are presented (“Ancestral”). Finally, the number of toxins and their pair-wise identity is calculated relative to all participants in recombination events (“All”) or only those with known activities (“With hosts”). (**a**). Dependence between the pairwise identity between recombinants and certain parents based on the host spectrum for toxins only, or the strains if the respective information for toxins is unavailable (**b**). The shape of the points encodes the type of the parent. The color indicates the change in affected orders. (**c**). Mean estimates of the total number of parents and recombinants in recombination events attributed to alterations in host species. Here and after the asterisks correspond to significant differences between the mean values using the Wilcox test corrected for multiple comparisons with the Benjamini-Hochberg procedure (**d**). Average estimates of the pair-wise sequence similarity between parents and children from the sets of events in which the host range differed or remained intact, respectively.

**Fig. S28**. The distribution of the event-wise similarity between the affected species hosts in recombinants and major/minor parents. Two datasets are presented, namely: the data for toxins only (**a**) or the combined data with strain annotations in case the respective data are available for strains only (**b**). In addition, we applied the ancestral state reconstruction procedure (“Ancestral”) or used the activity spectrum as it stands (“Unchanged”). Each panel consists of three major blocks, namely: (i) the transferred domain for each recombination event plotted on the Y-axis; (ii) the changes in orders, including parent-child comparisons and the global classification (“Global”); (iii) the similarity between the sets of affected host species. To assess similarity, the Szymkiewicz–Simpson coefficient (overlap coefficient) was used. Both the size and intensity of the color are proportional to the calculated coefficient. In the middle plots, the events are considered different if at least one order affected by children and parents was unequal.

**Fig. S29**. The global characteristics in the composition of affected host species for parents and children within recombination events. The similarity between host sets is calculated using the Szymkiewicz–Simpson (overlap) coefficient. Several approaches were applied to consider the host range data, namely: (i) annotations or toxins only (“Toxins”) or the data complemented with strain activities (“With strains”); (ii) the usage of the ancestral reconstruction procedure (“Ancestral”) or considering the data per se (“Unchanged”); (iii) performing pair-wise comparison between all sets of recombinants and parents using the joint host sets (“Events”) or considering single parent-child comparisons between toxin pairs independently (“Toxins”). (**a**) Density plots showing the frequency of overlap coefficient estimates within the events implying exchanges of the first and the third domains. The color of the plot corresponds to the type of parents, and the panels from top to bottom are determined by the sets of events depending on the source of host metadata. The distribution for the second domain was unavailable to calculate, given the scarcity of the data. (**d**) Mean overlap coefficient estimates between hosts and parents from recombination events grouped according to changes in host orders. The events are classified as the same only if the sets of affected orders are completely identical. Significant differences according to the Wilcox test with *P* values adjusted using the Benjamini-Hochberg procedure are marked with asterisks. (**c**) The relationship between the intersection of the host species attributed to recombinants and parents. The size of the points is proportional to the number of toxins. The shape corresponds to parent types, and the color is determined by the transferred domain.

**Fig. S30**. The number of affected hosts of toxins unaffected by domain swapping and those susceptible to recombination exchanges, namely, recombinants and parents of certain types. The toxicity spectrum was taken from the data for toxins only (“Toxins”) or complemented with strain activities as well (“With strains”). Furthermore, host sets were taken without any ancillary procedures (“Unchanged”) or after performing ancestral state reconstruction (“Ancestral”). Toxins that are associated with fewer than 15 species were taken into account to exclude bias. (**a**) The mean number of the affected host and orders of different toxin types, namely recombinants, parents, and toxins without recombination signals, determined by the color of the violins/box plots. (**b**) Average estimates for the number of affected host species for the mentioned groups. (**c**) The number of host orders and species (**d**) for a more detailed dataset considering major and minor parents independently. Significant differences according to the Wilcox test with *P* values adjusted using the Benjamini-Hochberg procedure are marked with asterisks.

**Fig. S31**. Factors delineating the number of affected host species and orders in recombinants and parents. Here, several datasets were considered, namely: (i) annotations or toxins only (“Toxins”) or the data complemented with strain activities (“With strains”); (ii) the usage of the ancestral reconstruction procedure (“Ancestral”) or considering the data per se (“Unchanged”); (iii) performing pair-wise comparison between all sets of recombinants and parents using the joint host sets (“Events”) or considering single parent-child comparisons between toxin pairs independently (“Toxins”); (iv) activities against orders only (“Orders”) or individual species (“Species”). (**a**). Total average estimates of the joint number of affected hosts for all parents and recombinants classified according to alterations in host sets. The events are called different if the sets of host species/orders are not completely identical between parents and children. (**b**). The mean estimates of the affected hosts according to the role of the toxins in recombination events, i.e., recombinants, minor, and major parents. (**c**). Linear models with associations between the similarity of the affected species (overlap coefficient) and the number of affected hosts. The shape of the dots corresponds to the type of parent with which recombinants are compared accordingly. The color displays the domain transferred from the minor parent in the event.

**Fig. S32**. Revealing the common origin of the affected host species and orders for parents and recombinants. In all plots, several data sources were used, namely: (i) host range for orders (“Orders”) or species (“Species”); (ii) activities for toxins only (“Toxins”) or toxins coupled with strains (“With strains”); (iii) host groups for toxins subjected to the ancestral reconstruction procedure (“Ancestral”) or without such processing (“Unchanged”). The pairs are considered the same in the case that at least one host species or order is present in any representative in the compared pair. The types of pair-wise comparisons were conducted: (i) recombinants with minor parents (“Rec_Min”); (ii) recombinants with major parents (“Rec_Maj”); (iii) minor and major parents with each other (“Min_Maj”). (**a**) Event-wise distribution of the intersections in the sets of affected hosts based on the data for toxins only and strains (**b**) with toxins jointly. (**c**) The total frequency of toxins marked as different and the same according to the presence of common hosts, and the percentage (**d**) of such groups. (**e**) The comparison of the distributions of different and same toxin pairs considering host orders and species (**f**) using bootstrap-like subsampling with 1000 iterations from the experimental and random datasets. The percentage of pairs marked as different is plotted.

**Fig. S33**. The properties of assemblies and strains in terms of the types of toxins they contain in the context of recombination events. (**a**) The distributions of the number of toxins in the assemblies and strains (**b**) regarding the rank of the respective Cry proteins. (**c**) Upset plots displaying the number of assemblies and strains (**d**) in the context of the toxin types they include. The respective instances are classified according to the composition of toxins, namely, parents, recombinants, and the unaffected Cry proteins. Intersections indicate that the set of assemblies/strains contains different types of toxins. (**e**) The mean number of Cry toxins within different datasets, namely, strains, assemblies, and genomic regions within the assemblies, single contigs, plasmids, and chromosomes. (**f**) The fraction of particular toxin types in the datasets containing recombinants, parents (**g**), and unaffected toxins (**h**).

**Fig. S34**. Genomic context of genes encoding parents and recombinants from one event found in the same assemblies. (**a**) Loci surrounding recombinants and parents from individual recombination events. Shown are regions, plasmids, chromosomes, and contigs bearing the genes corresponding to chimeric toxins. Three tracks are presented, namely, genes, insertion sequences (IS), and genetic islands (GIs). The color of the blocks on the first track corresponds to the toxin role in recombination events, e.g., major and minor parents, recombinants, and unaffected toxins. (**b**) Comparison of the total number of IS and GIs presented in genome assemblies that contain genes coding for recombinants and parents from the same events. Grey bars display median estimates for all assemblies studied, genomes with *cry* genes, and with toxins subjected to recombination, which are marked with letters “a”, “c”, and “r” on the X-axis, respectively. Numbers represent IDs of recombination events. Event 52 was associated with genome assemblies; however, the genomic context was highly similar, thus, only one example is given. (**c**) Relative positions of Cry proteins on the full-length partitioned phylogeny that co-occur in the same assembly. The color of circles defines individual recombination events.

**Fig. S35**. Comparison between the amount and compositions of Cry toxins belonging to strains and assemblies, including participants in domain exchanges and the unaffected proteins. (**a**) The measure of similarity of all sets of toxins that strains and assemblies in which at least one parent is present. Event-wise comparisons using the Jaccard coefficient (the length of the intersection divided by the union of the sets) refer to toxin compositions in assemblies and strains attributed to minor and major parents of the recombination events. (**b**) Comparison between the above-described Jaccard coefficients exceeding zero when comparing recombinants with parents and only parents, respectively. (**c**) The number of toxins within assemblies and strains that contain toxins of the particular type exclusively, such as recombinant, parental, and unaffected Cry proteins. Asterisks denote significant differences according to the Wilcox test with *P* values adjusted using the Benjamini-Hochberg procedure.

**Fig. S36**. The number of affected host orders and encoded toxins by strains and assemblies with or without toxins subjected to domain exchanges. Only orders were used to prevent overestimation caused by the imbalanced data on species. For the host range, the data only for toxins (“Toxins”) or the mixed information for toxins and strains (“With strains”) were considered. Significant differences according to the Wilcox test with *P* values adjusted using the Benjamini-Hochberg procedure are marked with asterisks. (**a**). Distributions of the number of affected orders for recombination participants and unaffected (“Unaff”) toxins in strains and assemblies uniting all parents in a single group or splitting them according to their role (**b**) in recombination events. (**c**). Average values of the affected hosts in strains/assemblies containing recombinants and parents of a certain type regarding the change in the affected host range. The parent-child pairs are called different if the list of host orders is not equal. (**d**). The average number of toxins in the isolates containing toxins of a certain group, according to their role in recombination events. (**e**). Mean estimates of the number of toxins produced by isolates containing recombinants and parents classified according to alterations in affected host orders.

**Fig. S37**. The description of strains and assemblies according to the toxins they contain in the context of changes in host order ranges after recombination events. The events are viewed as different if at least one host in recombinants and parents, either major or minor ones, differs. Two main datasets are used, namely, (i) the host range for toxins only (“Toxins”) or the joint data for toxins and strains (“With strains”); (ii) plain host sets as they come (“Unchanged”) or ancestrally reconstructed activities (“Ancestral”). (**a**). The frequency of strains and assemblies containing recombinants and parents of a particular type, with or without host specificity changes, and the total proportion (**b**) of these isolates in the whole dataset. (**c**). The bootstrap simulations with 1000 iterations of the difference ratio estimates. The ratio represents the percentage of parent-child pairs defined as different in terms of host specificity. Three groups of density plots are presented, namely comparisons between recombinants and minor parents (“Rec_Min”), recombinants and major parents (“Rec_Maj”), and the random distributions (“Rec_Simulated”).

**Fig. S38**. The similarity between strains and assemblies from the same recombination events in terms of the produced toxin sets and activities. The isolates are classified according to the toxins they contain regarding their role in recombination events and alterations in host specificity. They are called different if the affected orders for toxins acting as parents or children are not entirely identical. The toxicity spectrum is taken from the metadata available for toxins only (“Toxins”) or is complemented with the respective information from strains (“With strains”). (**a**) The density plots describing the distribution of similarity measures between the sets of affected host species using the overlap and Jaccard (**b**) coefficients. (**c**) The mean estimates of the overlap and Jaccard (**d**) coefficients for the sets of host species attributed to pairs of parent-child comparisons regarding the type of the parent in the events with or without host specificity changes. Significant differences according to the Wilcox test with *P* values adjusted using the Benjamini-Hochberg procedure are marked with asterisks. Figures (**e**-**h**) represent the identical information describing the similarity between the spectra of produced Cry toxins within the respective datasets.

**Fig. S39**. Relationships between the properties of the strains attributed to recombination-affected toxins with the same or distinct host specificity. The isolates are considered the same if the sets of affected orders in parental and child toxins are completely identical, and different otherwise. The activity spectrum is based on two data sources, namely, toxins only (“Toxins”), and the combined information from toxins and strains (“With strains”) if the respective toxin-wise data are unavailable (**a**). Linear models illustrating the dependence between the number of toxins and host orders in strains producing recombination-affected Cry toxins. The color of the dots encodes the type of the toxins (i.e., recombinants, minor and major parents). The shape corresponds to the domain transferred from the minor parent. (**b**). The dependency between the similarity estimates between the sets of affected host species using the Jaccard and overlap coefficients; associations between the similarity of the produced toxin spectra (**c**); and the number of recombinant and parental toxins (**d**). The shape of the dot determines host specificity alterations. The color indicates the type of toxins. The alpha channel and the size of the points correspond to the total number of Cry toxins in the isolates containing recombinants and parents, respectively.

**Fig. S40**. The key factors delineating the number of affected hosts in the isolates producing toxins participating in recombination events. The strains are classified according to host specificity changes, i.e., they are called the same if the sets of host orders in recombinants and parents are identical and different otherwise. The two datasets are considered, namely, the usage of annotations for toxins only (“Toxins”) or a joint dataset complemented with the data from strains (“With strains”). (**a**). Linear models demonstrating the dependence between the number of host orders in the isolates producing recombinants and parents from domain swapping events. The shape of the dots codes for specificity alterations. The color corresponds to the type of the parent. The points are sized according to the number of toxins in the isolates producing parental toxins, whereas the transparency indicates the number of recombinants. (**b**). Global correlation plot between certain factors in the isolates attributed to certain parents. Each dot demonstrates correlation estimates when comparing recombinants and minor/major parents in the respective isolates. The following characteristics are considered: the number of host orders in parental (“Hosts_P”) and recombinant strains(“Hosts_R”), the number of toxins (“Tox _P” and “Tox_R”), the similarity in the sets of affected host species using the overlap and Jaccard coefficients (“Sim _sp” and “Jac _sp”), and the same estimates relative to the sets of toxins (“Sim _tox” and “Jac _tox”). The color is proportional to the Pearson correlation estimates. The shape and the size indicate the significance level with the FDR (False Discovery Rate) adjustment. (**c**). The main linear models showing integral associations between the strains containing recombination-affected toxins. Plotted on the X- and Y-axes are the similarities in the sets of affected species using the overlap and Jaccard coefficients, respectively. The color indicates the parent type relative to which comparisons between the isolates encompassing recombinants and parents of a certain group. The shape and transparency of the dots encode the number of hosts in the isolates attributed to recombinants and parents. The size of the dots is proportional to the number of toxins in recombinant isolates.

**Fig. S41.** Weighted specificity-wise graph of strains sharing the common Cry toxins. The nodes represent strains, whereas edges indicate that at least one toxin within the third rank is shared by the strain. The weight of the edges depends on the number of common toxins. The color of the node represents the overall proportion of species that are affected by the toxins found in the strain. Shown are connected components containing at least four nodes. The IDs of the respective components are presented in numerical form.

**Fig. S42**. Overall properties of connected components of the strain graph and comparison between components. (**a**) The frequency of connected components according to the number of nodes (strains) forming them, the arrow points to the largest component. (**b**) The relationship between the number of toxins within the components and the number of recombination events, which are associated with them. The grey dashed line shows the dependencies estimated by the linear model. The size of the dots is proportional to the decimal logarithm of the number of nodes. (**c**) Distribution of the overlaps between the connected components in which parents and recombinants from the particular event reside. The color indicates the type of comparisons, namely, recombinants with parents, either minor, major, or all, and only between parents, respectively. The similarity is calculated using the Jaccard coefficient. (**d**) Comparisons between the components containing parents and children with the ones devoid of them in terms of the number of nodes (strains), individual toxin types (**e**), and orders affected (**f**). Significant differences according to the Wilcox test are marked with asterisks.

**Fig. S43**. Features of the connected components, including recombinants, parents, and unaffected toxins, in terms of the hosts they exert an effect on. (**a**) The percentage of host species attributed to their orders for strains within the connected components comprising toxins that have undergone recombination or do not contain them. Plotted on the X-axis are individual components. (**b**) The distribution of affected orders for the components associated with toxins from recombination events. The size of the sectors of the pie charts is proportional to the fraction of host species belonging to particular orders. The left adjacent panel indicates transferred domains within the events. (**c**) Density plots showing distributions of similarity estimates regarding the sets of orders and species (**d**) affected by recombinants and parents, either minor or major. The plots are presented only for the first and the third domains due to the scarcity of data regarding the second domain. Two similarity measures are considered, namely the Jaccard and overlap coefficients.

**Fig. S4****5**. Combined graph showing relationships between subpopulations of bacterial strains based on recombination events between toxins they produce. In the graph, nodes represent the connected components in the weighted specificity-wise graph of strains sharing the common Cry toxins (Fig. S41). The color of the node represents the overall proportion of species that are affected by the toxins found in all strains within the connected components. Edges show domain exchanges between the toxins within the connected components, colored according to the transferred domains. The edges represent all parents grouped according to domains (**a**) or the recombination events classified according to domains obtained from minor parents (**b**).

**Fig. S45**. Co-occurrence of toxin types in strains and strain graph-based connected components encompassing recombinant and parents from recombination events. (**a**) Linear models showing relationships between the number of recombinants and major parents, recombinants and minor parents (**b**), and recombinants and distinct types of parents (**c**) themselves. (**d**). A total correlation plot showing pair-wise correlations between the number of different toxin types found in isolates and connected components, namely, recombinants (“Rec”), minor parents (“Min”), and major parents (“Maj”). The color indicates the Pearson correlation statistics. The size and shape of the points are proportional to the FDR-adjusted *P* values (False Discovery Rate).

**Fig. S46**. The distribution of generalist and specialist strains and connected components in the dataset. The units are attributed to the generalist group if they affect more than one host order, and are called specialists otherwise. In addition, the isolates and components are classified according to the toxin types they contain, namely, recombinants (“Rec”), minor parents (“Min”), and major parents (“Maj”). Most of the tiles are colored according to the unit groups. The leftmost strip encodes the domain transferred from the minor parents in domain exchanges. (**a**). Event- and toxin-wise distribution of generalist and specialist connected components from the strain graph. The transparency depends on the number of components in which the respective toxins reside. (**b**). The total number and the percentage (**c**) of toxin-containing connected components that are marked as specialists and generalists. Plotted on (**d**-**f**) is similar information based on the individual strains.

**Fig. S47**. Key properties of the strains and connected components classified as generalists and specialists. Each strain is considered a generalist in the case that it affects more than one host order. An additional grouping is based on the host specificity change in parents and children residing in the analyzed isolates. The events are called different if the sets of host orders in parents and children are not identical. If the specificity spectrum is unavailable, the events and the respective isolates are placed in the unknown group. (**a**). The total frequency and the percentage (**b**) of generalist/specialist strains/connected components containing toxins of a certain type according to their role in recombination events. (**c**). The number of affected host orders and produced toxins (**d**) in the isolates and connected components according to their attributions to generalists/specialists, the type of toxins they encompass, and host specificity changes. Significant differences according to the Wilcox test are marked with asterisks.

**Fig. S48**. The key factors in generalist/specialist isolates and connected components in the strain graph determining the number of affected hosts. The units are grouped according to: (i) generalism (generalists/specialists), (ii) the type of toxins they produce according to recombination events, and (iii) host specificity changes. The units are considered generalists in case they affect more than one host order. (**a**). The linear models representing associations between the number of affected hosts and toxins for the isolates containing parents and recombinants. The color of the points codes for the type of toxins considered. The shape corresponds to host specificity alterations. The size is proportional to the total number of independent recombination events attributed to toxins within the units. (**b**). The global correlation plot describing associations between certain properties of the isolates and connected components in which recombination-affected toxins reside. The color indicates the correlation statistics calculated with the Pearson test. The size and the shape jointly indicate the significance level according to FDR-corrected (False Discovery Rate) *P* values. The following characteristics are considered: the number of host orders for the isolates (“Num_hosts”) and recombination-affected toxins per se (“Num_hosts_tox”), the number of produced toxins (“Num_toxins”), recombination events in the units (“Num_events”), and the percentage of generalist strains/components related to certain recombination events (“Gen_percent”).

**Fig. S49**. The comprehensive characteristics of recombination strategies in terms of host specificity changes. The considered strains and connected components are classified as generalists/specialists according to the number of host orders they infect. If the unit is associated with more than one host order, it is called a generalist. Furthermore, the groups of isolates and larger populations are viewed in the context of host specificity changes in toxins participating in recombination events. They are termed the same only if the sets of activities in parents and children are completely equal. (**a**). The distribution of recombination events according to host specificity alterations is defined above. The leftmost plot indicates the domain obtained from the minor parent. (**b**). The characteristics of strains/connected components containing recombination-affected toxins, with the total frequency and percentage of generalists. The transparency depends on the number of units related to the toxin described. (**c**).Strategies attributed to the recombination events. The order goes as follows: recombinant-minor parent-major parent. The total characteristics of the isolates depend on the percentage of generalists. At least 70% of strains/components should be generalists to attribute the whole group to this class. Therefore, the SSS strategy implies that the units containing recombinants and both types of parents are specialists (**d**). Host specificity spectrum of toxins and isolates/components related to domain exchanges. Two proportions are given, namely, the number of orders only or the percentage of species attributed to the respective host orders. (**e**). The number of host orders in recombination-affected toxins, as well as the mean estimates for strains/components, is coupled with the average number of toxins they encompass. The dots are colored according to the class of the toxins in recombination events, whereas the size defines the number of hosts or toxins. (**f**). The total number of individual recombination events related to toxins found in the isolates/connected components, and the number of such units.

**Fig. S50**. The common properties of the strains and connected components attributed to distinct recombination strategies. Each strategy depends on the final type of the units in which certain toxin types are found, following the order recombinants, minor-parents, major-parents. Thus, the SSS strategy implies that the exchanges were constrained by specialists only. The individual unit is termed a generalist if it is associated with more than one host order, whereas the whole group is considered a generalist if at least 70% of the units in it are generalists accordingly. (**a**) The distribution of the strategies assigned to the individual recombination events and strains/connected components (**b**). Each strategy is studied independently for distinct types of toxins found in the isolates, namely, recombinants (“Rec”), minor parents (“Min”), and major parents (“Maj”). (**c**) The average number of affected host orders in the isolates and connected components classified according to recombination strategies, and the number of hosts in recombination-prone individual toxins in these population units (**d**). Significant differences are marked with asterisks according to the Wilcox test.

**Fig. S51**. The properties of the population groups attributed to recombination strategies according to the toxins they contain, regarding associations between certain factors. The classification of the strategy depends on the final attribution of populations containing toxins of specific types as generalists/specialists. If at least 70% are generalists, the whole group is called a generalist. The order of the naming depends on the type of toxins, namely, recombinants, minor parents, and major parents. If the strategy is termed GGG, the exchanges occur between generalists (**a**). The average number of independent recombination events and toxins (**b**) in diverse population groups containing toxins following the defined recombination strategies. Significant differences according to the Wilcox test are marked with asterisks. (**c**). The integral correlations between the properties of the isolates and connected components grouped according to the recombination strategies they engage in. The color is proportional to the correlation estimate evaluated with the Pearson correlation test. The size and the shape of the points display the significance level based on FDR-adjusted *P* values (False Discovery Rate). The following characteristics are considered: the number of host orders for the isolates (“Hosts”) and recombination-affected toxins per se (“Hosts_tox”), the number of produced toxins (“Toxins”), recombination events in the units (“Events”), and the percentage of generalist strains/components related to certain recombination events (“Gen_percent”).

**Fig. S52**. Results of evolutionary selection assessment calculated on branches. (**a**) Optimal evolutionary models calculated on branches in which recombinant and parental sequences were marked. The selection pressure was determined on the sequences of different domains independently. The heatmap shows the models reported for the particular domain. The left adjacent subpanels correspond to the domain that was transferred from the minor parent within domain exchanges and show whether a parent, either minor or major, is unknown, i.e., absent in the dataset analyzed. The color of the blocks represents the model chosen, namely, positive and purifying selection. The panel is divided into two blocks, with the first one associated with parents and the second with recombinants, respectively. The right subpanel indicates whether, for at least one domain, a different selection pattern from the respective parental sequence exists. (**b**) Mean ω values (the nonsynonymous to synonymous substitution rate ratio, dN/dS) based on branch models. “All” denotes non-filtered inferences, “bfree” indicates events for which the LRT (log-likelihood test) revealed significant differences from the neutral model, “bneut” marks the set of events for which selection within the foreground branches differs from the overall background (**c**) Relationships between ω and the number of marked toxins (recombinants and parents) or the total number of toxins (**d**) within the subtrees tested. The shape of the points indicated the domains for which models were assessed. The color corresponds to the domain transferred during recombination. The ω value is positively and significantly correlated (*P* < 0.05 according to the linear model) with the number of marked toxins in the tree (both parents and recombinants), which, however, was backed only by several examples with a larger number of proteins (**c**). If all toxins are considered (**d**), significance is retained for parents only. (**e**) Average omega ω values based on branch models for the events affecting the first and the third domains of parents and children, respectively.

**Fig. S53**. Distribution of sites subjected to evolutionary selection according to site models. The selection pressure was determined on the sequences of different domains independently. (**a**) The total proportion of conserved and positively selected sites revealed by best-fit site models determined by the LRT (likelihood ratio test). (**b**) The number of positively selected and conserved sites among domains within the sets of events grouped according to the transferred domain and the fraction of these sites (**c**) in exchanges of the first and the second domains. (**d**) The overall number of positively selected and conserved sites (**e**) for events affecting the first and the third domains. The color corresponds to the domain for which the selection model was determined.

**Fig. S54**. Revealing factors possibly delineating the number of positively selected sites according to evolutionary selection models calculated on sites. Best-fit site models are determined by the LRT (likelihood ratio test). The selection pressure was determined on the sequences of different domains independently. The shape of the points indicated the domains for which models were assessed. The color corresponds to the domain transferred during recombination. (**a**) Relationship between the number of positively selected sites and the total number of toxins in the subtrees for which evolutionary selection was assessed, number of recombinants or parents (**b**), ω values (the nonsynonymous to synonymous substitution rate ratio, dN/dS) based on branch models within the foreground for all events (**c**) and those with a significant difference between branches (**d**). Figures (**e**) and (**f**) show the same estimates for background ω.

**Fig. S55**. Inferences of positively selected and conserved sites calculated on branch-site models in which recombinant and parental sequences were marked. The selection pressure was determined on the sequences of different domains independently. (**a**) The heatmap sites with significant signals of evolutionary selection reported by best-fit branch-site models revealed by LRT (likelihood ratio test) in the cases when recombinants and parents (**b**) are marked. The left adjacent subpanels correspond to the domain that was transferred from the minor parent within domain exchanges and show whether a parent, either minor or major, is unknown, i.e., absent in the dataset analyzed. The site color represents whether the site is positively selected or conserved. The intensity of the color depends on the probability, ranging from 0.95 to 0.99. The right subpanel indicates the significant model on branches with recombinants marked, namely, selection and purifying selection, either for the foreground or background only, if the ω on branches is equal. (**c**) The number of positively selected and conserved sites determined using branch-site models among domains of recombinants and parents within the sets of events grouped according to the transferred domain and the fraction of these sites (**d**).

**Fig. 56** Analysis of factors possibly delineating the number of positively selected sites according to evolutionary selection models calculated using branch-site models in which recombinants and parents were marked. Best-fit site models are determined by the LRT (likelihood ratio test). The selection pressure was determined on the sequences of different domains independently. The shape of the points indicated the domains for which models were assessed. The color corresponds to the domain transferred during recombination. (**a**) Relationship between the number of positively selected/conserved sites and the total number of toxins in the subtrees for which evolutionary selection was assessed, number of recombinants or parents (**b**), ω values (the nonsynonymous to synonymous substitution rate ratio, dN/dS) based on branch models within the background (**c**) and foreground in branches with marked recombinants and parents (**d**).

**Fig. S57**. Dependence between host specificity changes and features obtained when evaluating evolutionary selection. Best-fit branch, site, and branch-site models are determined by the LRT (likelihood ratio test). The selection pressure was determined on the sequences of different domains independently. Relationship between changes in host orders (when at least one recombinant and one parent affect non-equal sets of orders) and ω values (the nonsynonymous to synonymous substitution rate ratio, dN/dS) based on branch models within the background (**a**) and foreground in branches with marked recombinants and parents (**b**), the number of positively selected sites reported by site models (**c**), branch-site models (**d**), and the number of conserved sites (**e**). The relationship between the set of common species against which recombinants and parents are active and ω on foreground (**f**), and the number of positively selected (**g**) and conserved (**h**) sites revealed by branch-site models. The color indicates the domain transferred. The shape of the dots corresponds to the domain for which the calculations were carried out.

**Supporting Tables Description**

**Table S1.** Reference clusters of Cry sequences used for the analysis. Presented are reference clusters obtained with CD-HIT software with a 95% identity threshold. Proteins from the BPPRC database are marked with the “BT_” prefix, whereas putative novel toxins contain the “NOV_” prefix with identity to the closest homolog. The postfix “all” indicates sequences based on full protoxin sequences, whereas the “_uniq” postfix indicates unique sequences of the activated toxins, excluding C- and N-terminal regions.

**Table S2.** Properties of the domain sequences of the studied toxins. The mean pair-wise identity with all domains in the dataset (based on unique sequences of the activated toxins, excluding C- and N-terminal regions), as well as the length of the domain nucleotide sequences, is presented. The “prot_type” column shows whether the toxin represents a reference sequence within the cluster (“ref”) or the whole dataset, including within-cluster toxins (“all”).

**Table S3.** Coordinate-wise conservation scores for nucleotide sequences of active toxins. Coordinate refers to the coordinate in the nucleotide alignments. The “prot_type” column shows whether the toxin represents a reference sequence within the cluster (“ref”) or the whole dataset, including within-cluster toxins (“all”). To estimate sequence conservation, the highest per-site frequency of a non-gap symbol in the multiple alignments of toxin sequences was calculated.

**Table S4.** Identity percent within reference clusters. The number of unique sequences of activated toxins with the respective mean pair-wise identity of the individual domains is given.

**Table S5.** Characteristics of reference clusters containing more than 10 toxins. The coordinates represent the relative positions of the mutations in the alignments of nucleotide sequences of activated toxins. The type of mutations is determined according to the respective protein sequences as indels, synonymous, and nonsynonymous substitutions. The alignment lengths and the number of toxins in the cluster are shown as well.

**Table S6.** Distribution of bacterial species in which the analyzed Cry toxin sequences were found. Presented are identities with the closest homolog from the BPPRC database in the case of putative novel toxins. The “prot_type” column shows whether the toxin represents a reference sequence within the cluster (“ref”) or the whole dataset, including within-cluster toxins (“all”).

**Table S7.** Topological comparisons of phylogenies based on sequences of the individual domains and a concatenated, partitioned alignment of all three domains. The former are marked with Arabic numbers, whereas the latter is encoded by the “f” index. Used metrics are quartet distance metrics (“Quart_dist”), Robinson-Foulds distance (“RF_dist”), and cophenetic distance (“Coph_cor”). The numbers represent topological similarities defined as 1 – distance.

**Table S8.** Mean pair-wise comparisons of nucleotide sequences of individual domains among the studied reference clusters.

**Table S9.** Properties of phylogenies based on sequences of the individual domains and a concatenated, partitioned alignment of all three domains. The former trees are marked with Arabic numbers and “dom” prefixes, whereas the concatenated partitioned alignment of all three domains is called “full”. Shown are mean tree support (“Supp”), the total length of branches (“Brlen”), consistency index (“CI”), retention index (“RI”), and tree balance indices, namely, Colles-like, Sackin, and Cophenetic.

**Table S10.** Raw output from RDP4 software with predicted recombination events. Coordinates of the breakpoints refer to the nucleotide alignment of the activated toxins.

**Table S11.** Recombination events revealed with the RDP4 tool. The presented set is cleaned of inferences marked as unrelated to recombination. Parents are classified as minor (“Min_par”) and major (“Maj_par”) according to the fraction of the sequence received by recombinants. Coordinates of the breakpoints refer to nucleotide alignments of the activated toxins. The column “Unknown_flag” indicates the type of parent absent in the dataset. The event is classified according to the transferred domain. Events containing “_partial” postfix represent exchanges affecting less than 70% of the domains. Coordinates of the domains within the recombinant sequences are marked with “d1-d3” prefixes. Mean *P* values for the sets of statistical tests reported by RDP4 are also presented. The remaining columns show mean pair-wise identity between sequences of individual domains when comparing recombinants with minor and major parents, respectively.

**Table S12.** A refined set of recombination events reported by the RDP4 software. Parents are classified as minor (“Min_par”) and major (“Maj_par”) according to the fraction of the sequence received by recombinants. Coordinates of the breakpoints refer to nucleotide alignments of the activated toxins. The event is classified according to the transferred domain. The dataset is devoid of partial exchanges affecting less than 70% of the domains. Columns with the “parent_” prefix represent parents based on the phylogenetic trees of the individual domains. For a detailed description of the filtering procedure, see Fig. S1a. Parents with the “_new” postfix indicate refined sets of minor and major parents according to the congruence in the phylogenetic trees. The column “Unknown_flag” indicates the type of parent absent in the dataset. Coordinates of the domains within the recombinant sequences are marked with “d1-d3” prefixes. Mean *P* values for the sets of statistical tests reported by RDP4 are also presented. The remaining columns show mean pair-wise identity between sequences of individual domains when comparing recombinants with minor and major parents, respectively.

**Table S13.** Properties of the sets of recombination events revealed using RDP4. The number of minor (“Num_mins”) and major parents (“Num_majs”), as well as recombinants (“Num_recs”) for each event, is given. Three datasets are considered, namely, all reported events (“raw_data”), the set of toxins devoid of partial exchanges affecting less than 70% of the domains (“filt_partials”), and refined events based on the congruence in domain-wise phylogenetic trees (“filt_tree”). For a detailed description of the filtering procedure, see Fig. S1a. The column “Unknown_flag” indicates the type of parent absent in the dataset.

**Table S14.** Domain-wise identity of nucleotide sequences between recombinants and parents within sets of recombination events revealed with RDP4. Three datasets are considered, namely, all reported events (“raw_data”), the set of toxins devoid of partial exchanges affecting less than 70% of the domains (“filt_partials”), and refined events based on the congruence in domain-wise phylogenetic trees (“filt_tree”). For a detailed description of the filtering procedure, see Fig. S1a. Domains are classified as “Transferred” and “Non_transferred” for the respective fractions of sequences that recombinants receive from the respective parents.

**Table S15.** Mean differences between the identities of the domains between recombinants and parents. The difference is calculated between transferred and non-transferred domains of major and minor parents compared with the domain sequences of recombinant toxins. Three datasets are considered, namely, all reported events (“raw_data”), the set of toxins devoid of partial exchanges affecting less than 70% of the domains (“filt_partials”), and refined events based on the congruence in domain-wise phylogenetic trees (”filt_tree”). For a detailed description of the filtering procedure, see Fig. S1a. The column “sd” represents the standard deviation.

**Table S16.** Comparison between the number of parents in two sets of events, namely, raw inferences reported by RDP4 and the refined dataset based on the congruence in domain-wise phylogenetic trees. For a detailed description of the filtering procedure, see Fig. S1a. The column “Unknown_flag” indicates the type of parent absent in the dataset. The initial sets of minor (“Min_par”) and major (“Maj_par”) parents are presented, as well as new parents marked with a “_new” postfix. Columns with the “parent_” prefix represent parents based on the phylogenetic trees of the individual domains. The total number of parents within the raw and refined datasets is given in columns “Par_all_init” and “Par_new”, respectively.

**Table S17.** Prediction of toxins absent in the dataset based on events with parents of unknown origin. To calculate the number of possible toxins representing unknown parents, the depths (d) of subtrees in a domain-wise way for each event with unknown parents were summarized. After that, leaves with unknown events were removed. All subtrees with depth values were analyzed, and the number of nodes containing recombinants f(d) and the number of leaves with parents p(d) were calculated. The total number of predicted toxins U(d) was calculated as follows: U(d) = n(d)*p(d)/f(d).

**Table S18.** The number of mismatches between the domain sequences of parents and recombinants. The analysis was performed on nucleotide alignments of individual domains from recombinants and minor or major parents. The type of event (“Domain”) is classified according to the domain transferred from the minor parent. The mismatch rate (“Mism_rate”) is calculated as the number of mismatches divided by the length of the alignments.

**Table S19.** Coordinates of the recombination events obtained using ClonalFrameML software. Coordinates refer to nucleotide alignment of the activated toxins. Inferences marked with the “NODE_” prefix indicate possible ancestral events.

**Table S20.** Recombination events predicted using Gubbins software. Coordinates refer to nucleotide alignment of the activated toxins.

**Table S21.** Recombination events revealed using the fastGEAR tool. Coordinates refer to nucleotide alignment of the activated toxins. Events are classified as recent or ancestral.

**Table S22.** Properties of connected components within the graph of recombination exchanges with nodes representing toxins and edges denoting domain exchanges. Presented are the IDs of recombination events from Table S12 included in the respective component. The column “Edges” includes the list of edges classified according to the transferred domain. Major parents contain two Arabic postfixes with the respective domains. The prefix “recs” indicated links between recombinants in case the events contain multiple descendants of domain exchanges. Node types refer to the type of toxins in recombination events, namely, recombinants (“rec”), parents (“par”), and those being both parents and recombinants in different events (“mixed”).

**Table S23.** Distribution of toxins according to their role in recombination events. The toxins are classified as parents, recombinants, and those being both parents and recombinants in different events (“both”), as well as toxins without signals of domain exchanges.

**Table S24.** Linked recombination events with chimeric toxins that received three domains from different parents. The upper table is a subset of Table S12. Minor (“Min_par”) and major (“Maj_par”) parents reported by RPD4 are shown. Columns with the “parent_” prefix represent parents based on the phylogenetic trees of the individual domains. For a detailed description of the filtering procedure, see Fig. S1a. The lower table summarizes all toxins considering domain-wise parents.

**Table S25.** Properties of pangenomes based on genome assemblies attributed to *Bacillus thuringiensis*. Three groups were considered, namely all assemblies, those containing Cry toxins, and those bearing participants of recombination. The number of pan-genes according to their abundance in genomes is presented. The alpha parameter is calculated according to Heaps’ law.

**Table S26.** Estimates of recombination level using ClonalFrameML software within genome assemblies attributed to *Bacillus thuringiensis.* Three groups were considered, namely all assemblies, those containing Cry toxins, and those bearing participants of recombination.

**Table S27.** The number of mobile genetic elements (MGE) within genome assemblies attributed to *Bacillus thuringiensis*. Three types of MGEs are considered, namely, prophages, genetic islands (GIs), and insertion sequences (IS). Three groups were considered, namely all assemblies (“all”), those containing Cry toxins (“cry”), and bearing participants of recombination (“rec”).

**Table S28.** Total number of genetic elements (MGE) within genome assemblies attributed to *Bacillus thuringiensis*. Two types of MGEs are considered, namely, genetic islands (GIs) and insertion sequences (IS). Two groups were considered, namely, assemblies containing Cry toxins (“cry”) and bearing participants of recombination (“rec”). The number of Cry toxins and participants of recombination is presented in columns “Cry_num” and “Rec_num”, respectively. The number of mobile genetic elements in which genes encode Cry toxins and the participants of recombination events are included in columns “Cry_mge” and “Rec_mge”.

**Table S29.** Coordinate-wise identity between sequences of the flanking sequences (100 bp long) surrounding breakpoints within recombination events. The respective alignments are based solely on parents. Two types of flanks are considered, namely, between the first and the second domains (“flank1”) and the second and the third domains (“flank2”).

**Table S30.** Revealing the optimal number of clusters based on the vectors with coordinate-wise identities between parental sequences within the flanking sequences (100 bp long) surrounding breakpoints within recombination events. Two types of flanks are considered, namely, between the first and the second domains (“flank1”) and the second and the third domains (“flank2”). The quality of clusterization is evaluated using silhouette analysis. Three metrics were used, namely Euclidean (“eucl_vec”), cosine (“cos”), and Manhattan distances (“sub_vec”). The dissimilarity matrices were built based on vectors representing per-site identities, both raw and with the roll mean procedure applied (marked with “_roll” postfix). Two clustering algorithms, such as hierarchical clustering (“hclust”) and k-means, were tested.

**Table S31.** The distribution of recombination events within clusters of regions flanking recombination breakpoints. Two types of flanks are considered, namely, between the first and the second domains (“flank1”) and the second and the third domains (“flank2”). Recombination event IDs correspond to Table S12.

**Table S32.** Comparison between the mean identity of peaks within the analyzed flanking regions and sequences of the whole domains, either those that were transferred from the minor parent to the respective recombinant, or the adjacent domain. Peaks (“peak”) taken from Fig. S18 within the analyzed flanking regions and sequences of the whole domains, either those that were transferred from the minor parent to the respective recombinant (“trans_ident”) and the adjacent domain (“adj_ident”). Two types of flanks are considered, namely, between the first and the second domains (“flank1”) and the second and the third domains (“flank2”).

**Table S33.** The distribution of k-mers in the alignments of the parental sequences for certain recombination events in the 100-bp regions before and after breakpoints. The column “Flank_type” indicates whether the analyzed region is located between the first/second (“flank1”) or second/third domains (“flank2”). For each numbered 11 bp-length k-mer, the identity within the k-mer region (“Kmer_ident”) is given. The columns “Median_ident” and “Std_ident” represent the mean and standard deviation estimates for all k-mers in the region. The “Threshold” column shows the threshold for saving a given k-mer to pick the longest chains of the conserved regions. It is calculated as the sum of the median and standard deviation values.

**Table S34.** Chosen conserved blocks according to the k-mer distribution. The k-mers are presented in the Tab. S33. The appropriate chains of consecutive k-mers are constructed from k-mers satisfying the identity threshold (“Threshold”). Presented are the beginning (“Block_start”) and end (“Block_stop”) relative coordinates and the length of the flank (“Block_len”). The “Block_seq_ident” column shows the identity for the obtained sequences, whereas the “Block_kmers_ident” column indicates the mean identity of the consecutive k-mers that was employed when finding conserved regions. The “Total_blocks” and “Break_pos” describe the total number of conserved regions for a certain recombination event and the minimal distance to the breakpoint, respectively.

**Table S35.** The description of the picked flanks closest to the breakpoints. The flanks are constructed according to the k-mer distribution from Tab. S33. All columns are identical to those described in the Tab. S34.

**Table S36.** Comparison between the conserved regions according to their position relative to the recombination breakpoints. Described are the basic statistics (mean and standard deviation – “SD”) for length and pair-wise identity of the sequences of the selected flanks closest to the breakpoints and the remaining ones. The full list of the flanks is available in the Tab. S34, whereas those proximal to the breakpoints are presented in Tab. S35.

**Table S37.** The assessment of clustering procedures applied for grouping conserved flanks for constructing consensus models. For a detailed description of the score, see Text S2.8. Each column represents the ratios of the method-wise estimates to the minimum/maximum values of the variables, depending on the type of clustering approach as well as the number of clusters included in the “Clustering_type” column. Each clustering method is denoted as follows: “<number of clusters>_<aligner>_<aligner mode>_<similarity evaluation mode>_hclust_<distance metrics>”. For most of the scores, basic statistics are taken into account, namely, median, mean, maximum, minimum, and standard deviation, marked with the respective prefixes/infixes. The prefix “GCA” corresponds to the ratio of the number of leaves (sequences) containing all the representatives from a certain event to the number of parents in the event per se. The “GCA_norm” column designates the percentage of events in the subtree of the diagram containing sequences from these events only. The “flanks” infix corresponds to estimates of the size of the clusters containing all participants of a certain recombination event. The columns with the “ident” infix represent local or global similarity values between the members of clusters. The “Quart_dist_score” column describes the topological similarity between the obtained dendrogram and the reference ML tree. “Dunn_hc_score” and “Shil_hc_score” columns comprise the data on the Dunn and silhouette estimates calculated with the distance metrics taken into account. The “Shil_total_score” and “Gap_total_score” correspond to the quality assessments using the silhouette and gap methods, irrespective of the distance metrics. The “num_clust” infix signifies the size of the individual clusters. Finally, the “Sum_score_all” is a sum of all the columns, whereas the “Group_score” contains the same estimate with the proportion calculated not relative to the whole distribution but to the values within a certain clustering type, i.e., only the difference between the number of clusters is considered. The “Integral_score” is evaluated as “Sum_score_all” + “Group_score” / 2.

**Table S38.** The composition of flank clusters according to the best clustering pipeline. The column “Flank_type” indicates whether the analyzed region is located between the first/second (“flank1”) or second/third domains (“flank2”).

**Table S39.** The properties of the pair-wise identities within flank clusters in the references and all toxins. The identity is calculated for each toxin pair from a certain cluster. The column “Cluster_type” denotes which of the two sets is presented, namely, only reference clusters (“Ref”) and all the toxins from the deduplicated dataset when disclosing the CD-HIT clusters obtained with a 95% identity threshold.

**Table S40.** The distribution of the length of the clustered conserved regions of the reference only and all Cry toxins. The column “Cluster_type” denotes which of the two sets is presented, namely, only reference clusters (“Ref”) and all the toxins from the deduplicated dataset when disclosing the CD-HIT clusters obtained with a 95% identity threshold.

**Table S41.** Descriptive statistics of small conserved flank clusters regarding toxins or all Cry sequences, respectively. Presented columns are the basic statistics, i.e., median, mean, maximum, minimum, and standard deviation estimates. The column “Cluster_type” denotes which of the two sets is presented, namely, only reference clusters (“Ref”) and all the toxins from the deduplicated dataset when disclosing the CD-HIT clusters obtained with a 95% identity threshold.

**Table S42.** The summary of matches with consensus models of flank clusters obtained using the GLAM2Scan program. For each toxin, all the hits (“Hit”) are given. The coordinates (“Start” and “Stop”) of the matches are given according to the length of the toxin. In the “Score”, GLAM2Scan-provided estimates are presented. The “Flank_ident” column shows the highest identity of the found region with the representatives of the respective cluster. Domain mappings are given in the columns starting from “D1”-“D3” prefixes. Each flank is classified according to the domain of the toxin into which the signal falls. In this context, the list of all region types intersecting with the coordinates of the signal is presented in the “Flank_dom_range” column, whereas the final classification according to the overlap percentage is given in the “Flank_dom_type” column. Each toxin is marked in the column “Ref_flag” as the representative of reference CD-HIT clusters obtained with a 95% identity threshold (“REF”) or the non-reference member of the cluster (“CLUST”). If the toxin is a participant in recombination events, it is coined as “Rec” in the “Rec_type” column. In such a case, the list of events (“Events”), the number of the exchanges (“Events_num”), their types according to the domain transferred from the minor parent (“Domains”), and the roles of the toxin in these events (“Rec_class”) are presented. Toxins devoid of recombination signals are marked as “Unaffected”.

**Table S43.** Summary description of the pairwise identity estimates for the members of the conserved flanks clusters regarding the toxin role in recombination events. Each value is based on the comparisons between a certain toxin group within a certain cluster. Toxin groups are presented in the “Toxin_class” column. Included are recombinants, parents, and pair-wise comparisons between parents and recombinants from the same events. If the toxins are devoid of recombination signals or do not belong to the same event, they are placed into the “Unaffected” category.

**Table S44.** Pangenome-wise associations with the presence of flank clusters in *cry* sequences from the dataset of *Bt* genomes. The associations are revealed using the Scoary2 software. The presence of at least one signal from a certain cluster (“Cluster”) in any *cry* gene in the *Bt* assembly is perceived as the positive phenotype. Most of the columns correspond to the standard output of the program except for the odds ratio (“_ratio” postfix) and the allele frequency (“_percent” postfix) evaluated for the genomes with the trait (“Pos_”) or without (“Neg_”) it. Yellow lines highlight associations with site-specific recombinases from the Xer family.

**Table S45.** Toxicity spectrum of analyzed toxins and strains. The infected species (“Host_sp”) and Orders (“Host_order”) are taken from literature sources. The column “Rank” corresponds to the established nomenclature of Cry toxins according to the overall identity between protein sequences exceeding 95%.

**Table S46.** Total number of affected hosts within strains and toxins within ranks according to the established nomenclature. The number represents the orders to which particular species are attributed.

**Table S47.** The number of affected orders and species for toxins and strains. Data for toxins are presented in a rank-wise way for the fourth (individual toxins) and third (group of proteins within pair-wise 95% identity) ranks.

**Table S48.** Total event-wise annotation of recombination participants in the context of their host range. Changes in the compositions are defined as “Different” and “Same” in case the activities are not the same or differ accordingly. Host change is calculated using four modes: (i) – the difference between recombinants and at least one parent – “Change_flag_global” (could be either for the sets of orders or species according to the “Count_type” column being equal to “Orders_only” or “From_species”, respectively), (ii) –the difference between recombinants and a certain parent – “Change_flag_ local”, (iii) “Const_change_flag” – similar to the “Change_flag_global” defines the difference between recombinants and any parent is considered but only orders are considered constantly, and (iv) “Orders_local_flag” – the difference between recombinants and parents of a certain type in terms of affected orders. The “Tox_type” column defines the type of toxins according to recombination events, namely, recombinants (“Rec”), minor parents (“Min”), and major parents (“Maj”). The number of recombinants, minor parents, major parents, and all toxins are presented in columns “Num_recs”, “Num_mins”, “Num_majs”, and “Num_all”. For convenience, given the long format of the table, the number of toxins defined in the “Tox_type” column is specified in the “Num_self” column. The data source is defined in the “Mode” column. The two modes are considered, namely, the usage of the metadata for toxins only (“Rank3”) or complemented with the data for strains containing these toxins (“Strains_add”). Rows without ancestral reconstruction are called “Recent” in the “Ancestral_flag” column, whereas ancestrally reconstructed data are named “Ancestral”. The number of toxins is calculated for all toxins (“All”) or those containing activity annotations (“Host_only”), which is specified in the “Num_type” column. In the “Identity” column, mean pair-wise identity estimates between parents and children are displayed. The columns “Sim_rec_min”, “Sim_rec_maj”, and “Sim_min_maj” indicate whether any host is common for the groups under consideration, i.e., minor parents with recombinants, major parents with recombinants, and parents of distinct types, respectively. The presence of intersections is called “Same”, whereas the absence of any common items is called “Different”.

**Table S49.** Distributions of affected hosts for recombinants and parents among revealed recombination events within the third rank of the established nomenclature. Shown are the species and orders to which they are attributed. The respective annotation refers to toxins within the third rank. The column “Same_flag” indicates whether at least one order differs between recombinants and parents.

**Table S50.** Distributions of affected hosts for recombinants and parents among revealed recombination events within the strains. Shown are the species and orders to which they are attributed. The respective annotation refers to toxins within the third rank, supplemented by data for strains in which toxins are found, in case the set of hosts is unknown for the proteins. The column “Same_flag” indicates whether at least one order differs between recombinants and parents.

**Table S51.** Total counts of recombination events according to host specificity changes in the affected toxins. The parent-child pairs are called the “Same” in the “Change_flag_local” column if the host composition is equal, and “Different otherwise. ” The “Mode” column defines the data source. Using the host range for toxins only is marked as “Rank3”, whereas the joint data for toxins and strains are named “Strains_add”. The rows marked as “Ancestral” in the “Ancestral_flag” column are those processed with the ancestral state reconstruction procedure. Contrarily, “Recent” pairs are considered as they are. The type of parental toxin sets compared to recombinants is presented in the “Tox_type” columns, namely: minor parents with recombinants (“Min”) or major parents with recombinants (“Maj”). The taxonomy rank for the affected host orders in the “Count_type” column corresponds to orders (“Orders_only”) or species (“From_species”), respectively.

**Table S52**. Bootstrap simulations of the ratio of events with or without host changes between recombinants and distinct parent groups. In the “Mode” column, the data source is described. The data were taken from toxin annotations only (“Rank3”) or toxins complemented with strains (“Strains_add”). The “Ancestral_flag” column indicates whether the toxicity spectrum was ancestrally reconstructed (“Ancestral”) or taken as it is (“Recent”). The type of parent with which recombinants are compared is specified in the “Tox_type” column: minor (“Min”) and major (“Maj”) parents are considered. The taxonomic level of the host range is presented in the “Count_type” column. Orders are marked as “Orders_only” whereas species are called “From_species”, respectively. The “Diff_flag_exp” and “Diff_flag_boot” contain difference ratio estimates for the observed and randomly simulated datasets, respectively. The difference ratio was calculated as the proportion of pairs with specificity changes. The “Raw_pval” column contains the *P* values using the Wilcox test, whilst in the “P_adj” column, the FDR-adjusted (False Discovery Rate) values are presented.

**Table S53**. Logistic regression results show the key factors affecting host specificity changes. The parent-child pair is considered different if the host ranges are not identical. The formula is based on three variables: (i) the type of the parent in comparison, (ii) the number of toxins in the event, and the average pair-wise identity between parents and (iii) recombinants. The datasets are based on four characteristics as follows: “<ancestral reconstruction>_<host specificity data source>_<taxonomic level of affected hosts>_<toxin group>”. Ancestrally reconstructed host ranges are marked as “Ancestral” and “Recent”. If the host specificity is taken for toxins only, it is called “tox”, whereas the combined metadata with strains are named “strains”. Species (“species”) and orders (“orders”) of hosts are specified in the third part. Finally, either all (“all”) or only annotated toxins with host specificity data (“hosts”) are studied. Factors significantly affecting host specificity alterations are written in bold and highlighted with a yellow background.

**Table S54**. Descriptive statistics of recombination events with or without host specificity changes when comparing recombinants with distinct parent groups. The activity changes are specified in the “Change_flag_local” column. Pairs in comparison are considered the same in the case that activity spectra are identical, and different if at least one inconsistency is found. The “Mode” column specifies whether the host range was taken from toxin annotations only (“Rank3”) or the merged data from toxins and strains were considered (“Strains_add”). The “Ancestral_flag” column indicates that the ancestral state reconstruction procedure was conducted (“Ancestral”) or the data were taken without changes (“Recent”). The parent-child type is defined in the “Tox_type” column, namely, comparisons between recombinants and major parents (“Min”) or recombinants with major parents (“Maj”). In the “Num_type” column, the toxin group considered is described with either all (“All”) toxins or only those with known host specificity (“Host_only”) being taken. Mean, median, and SD (Standard Deviation) estimates for the number of toxins in the groups (“Mean_tox”, “Median_tox”, and “SD_tox”) and pairwise parent-children identities (“Mean_ident”, “Median_ident”, “SD_ident”) are presented.

**Table S55**. Integrative characteristics of recombination events in the context of the similarity in affected species. The similarity measure is the overlap coefficient based on the sets of affected host species presented in the “Simpson_coeff” column. Host change is calculated using four modes: (i) – the difference between recombinants and at least one parent – “Change_flag_global” (could be either for the sets of orders or species according to the “Count_type” column being equal to “Orders_only” or “From_species”, respectively), (ii) –the difference between recombinants and a certain parent – “Change_flag_ local”, (iii) “Const_change_flag” – similar to the “Change_flag_global” defines the difference between recombinants and any parent is considered but only orders are considered constantly, and (iv) “Orders_local_flag” – the difference between recombinants and parents of a certain type in terms of affected orders. The “Mode” column represents the data source, such as the usage of the annotations for toxins only (“Rank3”) or a combined dataset for toxins and strains they reside in (“Strains_add”). The “Ancestral_flag” column indicates whether the toxicity spectrum was ancestrally reconstructed (“Ancestral”) or taken as it is (“Recent”). In the “Num_type” column, the toxin group considered is described with either all (“All”) toxins or only those with known host specificity (“Host_only”) being taken. The columns “Sim_rec_min”, “Sim_rec_maj”, and “Sim_min_maj” indicate whether any host is common for the groups under consideration, i.e., minor parents with recombinants, major parents with recombinants, and parents of distinct types, respectively. The presence of intersections is called “Same”, whereas the absence of any common items is called “Different”. The “Tox_rec” and “Tox_par” columns show the child-parent pair used for calculating the similarity estimates. The similarity is calculated between recombinants and parents, namely, minor (“Min”) and major (“Maj”) are specified in the “Tox_type” column. In the “Simpson_type” column, the dataset for calculating the overlap coefficient is specified. The data are used for a single toxin-toxin pair (“Toxins”) or comparing the whole sets of the affected host species for all parents of a certain type and recombinants presented in a single recombination event (“Events”). In the latter case, only the types of toxins are specified in the “Tox_rec” and “Tox_par” columns. The “Simpson_coeff_local” column contains the pair-wise estimates for pair-wise comparisons only (with a specific parent group), whereas in the “Simpson_coeff” column, comparisons with the whole sets of recombinants and parents are conducted. The “Num_hosts” column displays the number of affected host orders and species according to the data specified in the “Host_type” column.

**Table S56**. The averaged estimates of the similarity between affected hosts for recombinants and parents in the domain exchange events classified according to the domains (“Domain”) transferred from the minor parents. The data for the second domain are omitted due to the scarcity of observations. The similarity is calculated using the overlap coefficient based on the sets of host species against which recombinants and parents are active. The “Mode” column points to whether host specificity spectra were taken from the data for toxins only (“Rank3”) or supplemented with the activities of the strains (“Strains_add”). The “Ancestral_flag” column signifies whether the data were subjected to the ancestral state reconstruction procedure (“Ancestral”) or remained unchanged (“Recent”). In the “Simpson_type” column, the means of evaluation are described. More specifically, presented are the averaged estimates based on host ranges for parent-child pairs (“Toxins”) or more general comparisons uniting all host species from all parents and recombinants jointly (“Events”). Mean, median, and SD (Standard Deviation) values are available in the “Mean_simp”, “Median_simp”, and “SD_simp” columns.

**Table S57**. The averaged estimates of the similarity between affected hosts for recombinants and parents in the domain exchange events classified according to host specificity changes. The columns are mostly identical to what is described in Table S56, except for the grouping principle. In the “Const_change_flag” column, the description of the events according to alterations in host specificity at the order level is given. The events are considered different if at least one order associated with recombinants and parents differs. The similarity is calculated using the overlap coefficient based on the sets of host species against which recombinants and parents are active. The “Mode” column points to whether host specificity spectra were taken from the data for toxins only (“Rank3”) or supplemented with the activities of the strains (“Strains_add”). The “Ancestral_flag” column signifies whether the data were subjected to the ancestral state reconstruction procedure (“Ancestral”) or remained unchanged (“Recent”). In the “Simpson_type” column, the means of evaluation are described. More specifically, presented are the averaged estimates based on host ranges for parent-child pairs (“Toxins”) or more general comparisons uniting all host species from all parents and recombinants jointly (“Events”). Mean, median, and SD (Standard Deviation) values are available in the “Mean_simp”, “Median_simp”, and “SD_simp” columns.

**Table S58.** Relationships between the overlap of common hosts affected by parents and recombinants and the number of toxins or mean identity when comparing nucleotide sequences of the domains of parents and recombinants. The associations are found with standard linear regression models. The overlap is calculated using the Szymkiewicz-Simpson coefficient based on the sets of host species (“Simpson_coeff_local”). The linear model formula is based on four variables: (i) the type of the parent in comparison (minor/major), (ii) the average pair-wise identity between parents and recombinants, (iii) the type of the domain transferred from the minor parent, and (iv) the number of toxins in the event. The datasets are based on three characteristics as follows: “<host specificity data source>_<ancestral reconstruction_ <toxin group>”. If the host specificity is taken for toxins only, it is called “Rank3”, whereas the combined metadata with strains are named “Strains_add”. Ancestrally reconstructed host ranges are marked as “Ancestral” and “Recent”. Finally, either individual toxins (“Toxins”) or host sets from all toxins of a certain type (“Events”) are studied. Variables that are significantly associated with the overlap coefficient are written in bold and highlighted with a yellow background.

**Table S59.** Comparison of the number of affected hosts by different types of toxins, namely, unaffected by recombination, recombinants, and parents (minor and major). These groups are marked as “Rec”, “Min”, “Maj”, and “Unaffected” in the “Tox_type” column, respectively. The two datasets of host specificity data are used, namely, the data for toxins only or merged activities for toxins (“Rank3”) and strains (“Strains_add”) as specified in the “Mode” column. The “Ancestral_flag” column indicates if the ancestral state reconstruction was performed (“Ancestral”) or not performed (“Recent”). The host taxonomy ranks are specified in the “Host_type” column. To overcome the bias caused by considering the best-studied Cry toxins, the units affecting more than 15 species were excluded. Mean, median, and SD (Standard Deviation) estimates are available in columns “Mean_hosts_num”, “Median_hosts_num”, and “SD_hosts_num”.

**Table S60.** Descriptive statistics of the number of affected hosts for toxin sets grouped according to the patterns of host specificity changes. The classification is given in the “Const_change_flag” column. The events are considered different if the sets of affected orders for recombinants and parents are not exactly equal, and the same otherwise. The “Mode” column represents the data source, such as the usage of the annotations for toxins only (“Rank3”) or a combined dataset for toxins and strains they reside in (“Strains_add”). The “Ancestral_flag” column indicates whether the toxicity spectrum was ancestrally reconstructed (“Ancestral”) or taken as it is (“Recent”). The taxonomic rank of the hosts is described in the “Host_type” column. Mean, median, and SD (Standard Deviation) values are presented in the columns “Mean_hosts_num”, “Median_hosts_num”, and “SD_hosts_num”, respectively.

**Table S61.** Extended averaged data on the number of affected hosts for Cry toxins from the recombination events grouped according to the toxin type and the alterations in host changes. The table combines classifications from Tables S59-60. The “Mode” column points to whether host specificity spectra were taken from the data for toxins only (“Rank3”) or supplemented with the activities of the strains (“Strains_add”). The “Ancestral_flag” column signifies whether the data were subjected to the ancestral state reconstruction procedure (“Ancestral”) or remained unchanged (“Recent”). Toxin groups according to recombination events are marked as “Rec”, “Min”, “Maj”, and “Unaffected” in the “Tox_type” column, respectively. The “Host_type” column indicates the taxonomic rank of hosts. Alterations in host specificity are present in the “Const_change_flag” column. The parent-children comparison results are called the same in case the sets of affected host orders are identical, and different in the other case. Mean, median, and SD (Standard Deviation) estimates are presented in the columns “Mean_hosts_num”, “Median_hosts_num”, and “SD_hosts_num”, respectively.

**Table S62.** Correlations between the similarity in affected host sets for parents and children and the number of affected hosts. Each dataset is encoded according to the scheme described in Table S58: “<host specificity data source>_<ancestral reconstruction_ <toxin group>”. If the host specificity is taken for toxins only, it is called “Toxins”, whereas the combined metadata with strains are named “Strains_add”. Ancestrally reconstructed host ranges are marked as “Ancestral” and “Recent”. Finally, either single parent-child pairs (“Toxins”) or the whole spectra for all recombinants and parents of a certain type within specific recombination events (“Events”) are analyzed. The correlation estimates (“Pearson_corr”) are obtained using the Pearson correlation method. Significant associations are highlighted in a yellow background and written in bold.

**Table S63.** The total event-wise characteristics of overlaps in affected host range lists for recombinants and parents of certain types. In the “Sim_flag” columns, the overlap is marked as “Same” in the case that at least one host is common for parents and recombinants or minor and major parents between themselves. The comparison type is presented in the “Sim_type” column, namely: recombinants with minor parents (“Rec_min”), recombinants with major parents (“Rec_maj”), and parents between themselves (“Min_maj”). The “Mode” column represents the data source, such as the usage of the annotations for toxins only (“Rank3”) or a combined dataset for toxins and strains they reside in (“Strains_add”). The “Ancestral_flag” column indicates whether the toxicity spectrum was ancestrally reconstructed (“Ancestral”) or taken as it is (“Recent”). The taxonomic rank of the hosts is described in the “Host_type” column with “Orders_only” and “From_species” indicating the analysis of orders and species, respectively.

**Table S64.** The total frequency of parent-children pairs in terms of changes in host specificity in the context of recombination events. The pairs are classified according to the presence of common hosts, i.e., the “Same” characteristic in the “Sim_flag” column stands for the presence of intersections in the lists of affected hosts, whereas rows marked as “Different” do not contain any common activities. The “Sim_type” column specifies which toxin types are considered, namely, comparisons between recombinants and minor parents (“Rec_min”), recombinants and major parents (“Rec_maj”), and different parents per se (“Min_maj”). Three columns indicating the data source go as follows: (i) the toxicity spectrum (“Mode”) taken from toxins only (“Rank3”) or toxins complemented with strains (“Strains_add”), (ii) the presence (“Ancestral”) or absence (“Recent”) of the ancestral state reconstruction procedure (“Ancestral_flag”), and the taxonomic classification of the hosts (“Count_type”), i.e., orders (“Orders_only”) and species (“From_species”).

**Table S65.** The characteristics of recombination events regarding host specificity after the ancestral reconstruction procedure. The host order range for recombinants, minor parents, and major parents is given in the “Recs”, “Mins”, and “Majs” columns. The “Common_all” column describes the intersections between host ranges or all toxin groups. In the “Type” column, the data source for host specificity is presented. The rows marked as “Toxins_only” contain annotations based on toxins only, whereas those specified as “With_strains” consider the joint data for toxins and strains accordingly. The yellow background highlights unequal host ranges between certain toxin types.

**Table S66.** Experimental and simulated distribution of the difference ratio obtained using a bootstrap procedure with 1000 iterations. The comparison type is presented in the “Sim_type” column, namely: recombinants with minor parents (“Rec_min”), recombinants with major parents (“Rec_maj”), and parents between themselves (“Min_maj”). The “Mode” column represents the data source, such as the usage of the annotations for toxins only (“Rank3”) or a combined dataset for toxins and strains they reside in (“Strains_add”). The “Ancestral_flag” column indicates whether the toxicity spectrum was ancestrally reconstructed (“Ancestral”) or taken as it is (“Recent”). The taxonomic rank of the hosts is described in the “Host_type” column with “Orders_only” and “From_species” indicating the analysis of orders and species, respectively. The “Diff_flag_exp” and “Diff_flag_boot” contain difference ratio estimates for the observed and randomly simulated datasets, respectively. The difference ratio was calculated as the proportion of the pairs with specificity changes, i.e., without any common hosts. The “Raw_pval” column contains the *P* values using the Wilcox test, whilst in the “P_adj” column, the FDR-adjusted (False Discovery Rate) values are presented.

**Table S67.** The total summary of recombination events in the context of host specificity changes. Four data sources were considered, namely: 1) the annotation source, such as the usage of the annotations for toxins only (“toxins”) or a combined dataset for toxins and strains they reside in (“strains”), and 2) performing the ancestral recomstruction procedure (“Ancestral”) or taken the data without modifications (“Recent”). Shown are the number of events with the same or different host ranges, with the latter having or lacking intersections in the sets of affected host orders. The yellow color highlights the summary estimates related to the events in which the number of affected hosts of recombinants is the same or lower than in the parents.

**Table S68.** Distribution of Cry toxins among strains of *Bacillus thuringiensis*. Shown are toxins found in each strain and the total number of these toxins.

**Table S69.** Distribution of Cry toxins among genome assemblies of *Bacillus thuringiensis*. Presented are assembly completeness levels (“Level”), and accession numbers of Cry proteins (“Prot_accession”) and contigs in which respective toxin-encoding genes reside (“Contig_accession”).

**Table S70.** The number of Cry toxins within different datasets, namely, strains, assemblies, and genomic regions within the assemblies, single contigs, plasmids, and chromosomes.

**Table S71.** Distribution of toxin-encoding genes within genomic regions of assemblies in which recombinants and parents from particular events are present. Accession numbers of assemblies, the respective strains, and genomic regions (chromosomes, plasmids, and contigs) are presented.

**Table S72.** The measure of similarity of all sets of toxins that strains and assemblies with recombination participants contain. Event-wise similarity measure (“Coeff”) is calculated using the Jaccard coefficient based on toxin compositions in assemblies and strains when comparing recombinants and parents (“Jaccard”) or only minor and major parents (“Par_coeff”).

**Table S73.** The number of toxins within assemblies and strains that contain toxins of the particular type exclusively, such as recombinants, parents, and unaffected Cry proteins. These groups are marked as “rec”, “par”, and “unaff”, respectively. Two datasets are considered, namely, assemblies and strains that are allowed to contain different types of toxins (“soft”) or bear particular types of toxins exclusively (“strict”).

**Table S74.** The number of affected host species and orders by strains and assemblies with or without toxins subjected to domain exchanges. Three types of toxins are considered, as stated in the “Tox_type” column, namely unaffected by recombination (“Unaffected”), recombinants (“Recombinants”), and parents (“Parents”). A more specific classification with minor parents (“Minor parents”) and major parents (“Major parents”) is given in the “Tox_rec_type” column. Strains and genome assemblies are considered as specified in the “Dataset_type” column, whereas the respective identifiers are given in the “Unit” column. The number of affected hosts and produced Cry toxins is stated in the “Host_num” and “Tox_num” columns, respectively. The “Mode” column describes the source of the host specificity data, i.e., the usage of the annotations for toxins only (“Rank3”) or combined metadata for toxins and strains they reside in (“Strains_add”). The “Dataset_tox_type” indicates that the data were used for general comparisons (“Common”), in which all parents were combined, or specific annotations for minor and major parents separately (“Parent_types”). The taxonomic level of hosts is described in the “Host_type” column.

**Table S75.** Mean estimates of the number of affected host orders for the isolates containing distinct Cry toxin types. Host specificity spectra were taken from the data for toxins only (“Rank3”) or joint annotations for toxins and strains (“Strains_add”) as displayed in the “Mode” column. The “Dataset_type” column defines whether genome assemblies (“Assemblies”) or strains (“Strains”) are described. Strains/assemblies are grouped according to toxins relative to recombination events, namely, recombinants, parents, irrespective of their type, or unaffected toxins, which is given in the “Tox_type” column. The mean, median, and SD (Standard Deviation) values are presented in the “Mean_hosts_num”, “Median_hosts_num”, and “SD_hosts_num” columns, respectively. The total number of isolates containing certain toxins is specified in the “Num_units” column.

**Table S76.** Mean estimates of the number of affected host orders for the isolates containing distinct Cry toxin types. Host specificity spectra were taken from the data for toxins only (“Rank3”) or joint annotations for toxins and strains (“Strains_add”) as displayed in the “Mode” column. The “Dataset_type” column defines whether genome assemblies (“Assemblies”) or strains (“Strains”) are described. Similar to Table S75, strains/assemblies are grouped according to toxins relative to recombination events specifying certain parent roles, namely, recombinants, minor parents, major parents, or unaffected toxins as specified in the “Tox_type” column. The mean, median, and SD (Standard Deviation) values are presented in the “Mean_hosts_num”, “Median_hosts_num”, and “SD_hosts_num” columns, respectively. The total number of isolates containing certain toxins is specified in the “Num_units” column.

**Table S77.** Mean estimates of the number of affected host orders for the isolates containing distinct Cry toxin types in the context of host specificity alterations. Host specificity spectra were taken from the data for toxins only (“Rank3”) or joint annotations for toxins and strains (“Strains_add”) as displayed in the “Mode” column. The “Dataset_type” column defines whether genome assemblies (“Assemblies”) or strains (“Strains”) are described. Similar to Table S75, strains/assemblies are grouped according to toxins relative to recombination events specifying certain parent roles, namely, recombinants (“Rec”), minor parents (“Min”), and major parents (“Maj”) as specified in the “Tox_type” column. Specificity changes are described in the “Orders_local_flag” column, and the parent-child pair is considered different if the host orders are not completely identical. The mean, median, and SD (Standard Deviation) values are presented in the “Mean_hosts_num”, “Median_hosts_num”, and “SD_hosts_num” columns, respectively. The total number of isolates containing certain toxins is specified in the “Num_units” column.

**Table S78.** Mean estimates of the number of produced Cry toxins found in the isolates containing distinct Cry toxin types. The datasets based on host specificity were taken from the data for toxins only (“Rank3”) or joint annotations for toxins and strains (“Strains_add”) as displayed in the “Mode” column. The “Dataset_type” column defines whether genome assemblies (“Assemblies”) or strains (“Strains”) are described. Strains/assemblies are grouped according to toxins relative to recombination events specifying certain parent roles, namely, recombinants, minor parents, major parents, or unaffected toxins as specified in the “Tox_type” column. The mean, median, and SD (Standard Deviation) values are presented in the “Mean_tox_num”, “Median_tox_num”, and “SD_tox_num” columns, respectively. The total number of isolates containing certain toxins is specified in the “Num_units” column.

**Table S79.** Mean estimates of the number of produced Cry toxins found in the isolates containing distinct Cry toxin types in the context of host specificity alterations. The datasets based on host specificity were taken from the data for toxins only (“Rank3”) or joint annotations for toxins and strains (“Strains_add”) as displayed in the “Mode” column. The “Dataset_type” column defines whether genome assemblies (“Assemblies”) or strains (“Strains”) are described. Strains/assemblies are grouped according to toxins relative to recombination events specifying certain parent roles, namely, recombinants (“Rec”), minor parents (“Min”), and major parents (“Maj”) as specified in the “Tox_type” column. Specificity changes are described in the “Orders_local_flag” column, and the parent-child pair is considered different if the host orders are not completely identical. The mean, median, and SD (Standard Deviation) values are presented in the “Mean_tox_num”, “Median_tox_num”, and “SD_tox_num” columns, respectively. The total number of isolates containing certain toxins is specified in the “Num_units” column.

**Table S80.** The total distribution of bacterial isolates containing Cry toxins of certain types in the context of host specificity changes after recombination events. The “Dataset_type” column defines whether genome assemblies (“Assemblies”) or strains (“Strains”) are described. The “Mode” column represents the data source, such as the usage of the annotations for toxins only (“Rank3”) or a combined dataset for toxins and strains they reside in (“Strains_add”). The “Ancestral_flag” column indicates whether the toxicity spectrum was ancestrally reconstructed (“Ancestral”) or taken as it is (“Recent”). Strains/assemblies are grouped according to toxins relative to recombination events specifying certain parent roles, namely, recombinants (“Rec”), minor parents (“Min”), and major parents (“Maj”) as shown in the “Tox_type” column. In the “Orders_local_flag” column, the child-parent pair is marked as “Same” in the case where affected hosts are identical for recombinants and parents under consideration. The comparison type is presented in the “Compare_type” column, namely: recombinants with minor parents (“Rec_Min”) and recombinants with major parents (“Rec_Maj”).

**Table S81.** Bootstrap-based assessment of the host specificity changes in toxins present in the known diversity of Cry-producing strains. The estimates were obtained using 1000 iterations for calculating the difference ratio. The ratio represents the fraction of the items marked as different. The children-parent pairs are considered different if the list of affected host orders in recombinants and parents is not equal. The “Dataset_type” column defines whether genome assemblies (“Assemblies”) or strains (“Strains”) are described. The “Mode” column represents the data source, such as the usage of the annotations for toxins only (“Rank3”) or a combined dataset for toxins and strains they reside in (“Strains_add”). The comparison type is presented in the “Compare_type” column, namely: recombinants with minor parents (“Rec_Min”), recombinants with major parents (“Rec_Maj”), and parents between themselves (“Min_Maj”). The “Diff_flag_exp” and “Diff_flag_boot” contain difference ratio estimates for the observed and randomly simulated datasets, respectively. The “Raw_pval” column contains the *P* values using the Wilcox test, whilst in the “P_adj” column, the FDR-adjusted (False Discovery Rate) values are presented.

**Table S82.** Similarity estimates between the isolates containing Cry toxins of distinct roles in recombination events, according to toxic activities and the spectrum of produced toxins. The “Dataset_type” column defines whether genome assemblies (“Assemblies”) or strains (“Strains”) are described. The “Mode” column represents the data source, such as the usage of the annotations for toxins only (“Rank3”) or a combined dataset for toxins and strains they reside in (“Strains_add”). The isolates are classified according to host specificity changes signified in the “Orders_local_flag” column. The parent-child pairs are called different if the sets of host orders are not exactly equal, and the same otherwise. The toxin group relative to which the comparisons are performed is specified in the “Tox_type” column. Recombinants (“Rec”), minor parents (“Min”), and major parents (“Maj”) are studied accordingly. The comparison type is presented in the “Compare_type” column, namely: recombinants with minor parents (“Rec_Min”), recombinants with major parents (“Rec_Maj”), and parents between themselves (“Min_Maj”). The similarity metrics include the overlap and Jaccard coefficient calculated using the activities against host species (“Sim_species” and “Jac_species”) and the sets of produced Cry toxins (“Sim_toxins” and “Jac_toxins”).

**Table S83.** The averaged values of pair-wise similarities in the context of affected host species and the range of produced toxins in the isolates containing recombination-affected *cry* loci. The usage of assemblies and strains is determined by the “Dataset_type” column. The source of toxicity data is described in the “Mode” column, with “Rank3” corresponding to toxins only and “Strains_add” indicating the combined dataset for toxins and strains. In the “Compare_type” column, the type of comparisons is presented, namely, recombinants with minor parents (“Rec_Min”), recombinants with major parents (“Rec_Maj”), and parents between themselves (“Min_Maj”). Host specificity changes described in the “Orders_local_flag” column correspond to the alterations in the affected host orders. If the spectrum between toxin groups is identical, the pairs are called the “same” or named “different” if the reverse is true. Mean, median, and SD (Standard Deviation) estimates based on the sets of affected host species using the overlap metrics are presented in the “Mean_sim_host”, “Median_sim_host”, and “SD_sim_host” columns, whereas those based on the Jaccard coefficient are described in “the Mean_jac_host”, “Median_jac_host”, and “SD_jac_host” columns. The analogous content according to the toxins is described in the “Mean_sim_tox”, “Median_sim_tox”, ” SD_sim_tox”, “Mean_jac_tox”, “Median_jac_tox”, and “SD_jac_tox” columns. The total number of strains and assemblies is presented in the “Num_units” column.

**Table S84.** The integral distribution of the comparisons between strains containing participants in recombination events regarding their role and host specificity changes. The “Dataset_type” column defines whether the assemblies or strains are used. The source of toxicity data is described in the “Mode” column, with “Rank3” corresponding to toxins only and “Strains_add” indicating the combined dataset for toxins and strains. The isolates are classified according to host specificity changes signified in the “Orders_local_flag” column. The parent-child pairs are called different if the sets of host orders are not exactly equal, and the same otherwise. The toxin group of the first isolate relative to which the comparisons are performed is specified in the “Tox_type” column. Recombinants (“Rec”), minor parents (“Min”), and major parents (“Maj”) are studied accordingly. The identifier of the first strain/assembly in the comparison, the number of affected host orders, and the produced toxins are specified in the “Unit1”, “Host_num”, and “Num_toxins” columns. Similar information for the second unit in comparison is described in the “Unit1”, “Host_num2”, and “Num_toxins2” columns. The similarity metrics imply the usage of the overlap and Jaccard coefficients according to the lists of affected host species (“Sim_species” and “Jac_species”) or the spectra of Cry toxins found in the isolates (“Sim_toxins” and “Jac_toxins”). The comparison type is presented in the “Compare_type” column, namely: recombinants with minor parents (“Rec_Min”) and recombinants with major parents (“Rec_Maj”).

**Table S85.** The global correlations between distinct properties of the isolates containing Cry toxins subjected to domain exchanges in the context of host specificity alterations. The correlations are calculated within the isolates containing recombinants and minor parents (“Min”) or major parents (“Maj”) as shown in the “Tox_type” column. The classification of the events is based on changes in affected host orders between parents and children. The pairs are called different if the spectrum of host orders is not entirely equal, and the same in the other case. The characteristics in comparisons within the considered isolates are presented in the “Param1” and “Param2” columns. The following data are studied, namely: (i) similarity metrics based on the produced toxins and affected host species based on the overlap and Jaccard coefficients (“Sim_species”, “Sim_toxins”, “Jac_species”, and “Jac_toxins”), (ii) the number of affected hosts and the spectrum of toxins in the first (“Host_num”, “Num_toxins”) and the second strain (“Host_num2”, “Num_toxins2”). The correlation estimates (“Pearson_corr”) are obtained using the Pearson correlation method. The “Pval” column contains the *P* values using the Pearson test, whilst in the “P_adj” column, the FDR-adjusted (False Discovery Rate) values are presented. The significance level is explicitly stated in the “Signif_level” column based on the adjusted *P* values.

**Table S86.** Properties of connected components of the strain graph linking strains that contain at least one common toxin. Shown are the number of host species (“Num_sp”) attributed to orders (“Host_type”), the total number of unique host orders (“Num_hosts”), toxins (“Num_tox_types”), strains (“Num_strains”), and recombination events in case toxins participate in domain exchanges (“Num_events”). Considered are all components or those containing toxins subjected to recombination (“Rec”).

**Table S87.** Distribution of parents and recombinants among connected components of the strains graph linking strains that contain at least one common toxin. Shown are components encompassing recombinants (“Rec_comps”), minor (“Min_comps”), and major (“Maj_comps”) parents. The similarity measure is calculated based on the sets of toxins in the components when comparing recombinants with parents (“Min_jac”, “Maj_jac”) or parents only (“Inter_par_jac”).

**Table S88.** Distributions of similarity estimates regarding the sets of orders and species affected by recombinants and parents, either minor or major. Two similarity measures are considered, namely the Jaccard and Szymkiewicz-Simpson coefficients, respectively.

**Table S89.** The global classification of strains encompassing recombination-affected toxins according to their host range. The “Tox_type” column indicates which toxin group, according to recombination events, is described, namely, recombinants (“Recombinants”), minor parents (“Minor”), and major parents (“Major”). The same characteristic is given in the “Both_type” column, with the toxins acting both as parents and recombinants in distinct domain exchange events being named “Both”. The described toxin is presented in the “Tox” column, whereas the units under consideration are denoted in the “Strain” and “Component” columns, describing individual strains and connected components in the strain graph, respectively. The type of the unit, i.e., connected components (“Components”) and strains (“Strains”), is given in the “Comp_type” column. The number of affected orders for a certain toxin is presented in the “Num_orders_tox” column, whereas the spectrum for the whole unit is presented in the “Num_orders” column. In the case of connected components, mean estimates for all the isolates within the component are given. The number of toxins is stated in the “Num_toxins” column. Components/strains are classified as generalists and specialists, as shown in the “Generalist_type” column, according to the number of affected hosts, being named generalists in case they affect more than one host order. In the case of connected components, those including more than 70% of generalist strains are termed generalists. The respective percentage is shown in the “Gen_percent” column. Host change is calculated using three modes: (i) “Change_flag_global” – the difference between recombinants and at least one parent – “Change_flag_global”, (ii) –the difference between recombinants and a certain parent – “Change_flag_ local”, and (iii) “Orders_local_flag” – the difference between recombinants and parents of a certain type in terms of affected orders. Affected orders for each isolate are listed in the “Host_order” column. The total number of individual recombination events found in the toxins within the isolates/connected components is described in the “Num_events” column.

**Table S90.** The co-occurrence of the toxins classified according to their role in recombination events in the isolates and bacterial populations. Both individual strains (“Strains”) and connected components from the strain graph (“Components”) are considered as specified in the “Unit_type” column. The units are called generalists in case they affect more than one host order, as defined in the “Generalist_type” column. For connected components, not less than 70% of the included isolates should be generalists to call the whole component a generalist. The number of recombinants (“Rec_num”), minor parents (“Min_num”), and major parents (“Maj_num”) from distinct recombination events is presented.

**Table S91.** The statistical validation of the co-occurrence between toxins of diverse roles in the recombination events within the bacterial isolates. The underlying data were taken from Table S89. The “Comp_type” column indicates whether individual strains (“Strains”) or the whole connected components from the strain graph (“Components”) were used. The “Generalist_type” column classifies the dataset as generalists and specialists with the former affecting more than one host order. In the case of the whole connected components, those uniting at least 70% of generalist strains are called generalists. The types of toxin pairs in comparison are given in the “Tox_type” and “Tox_type2” columns. Recombinants (“Rec_num”), minor parents (“Min_num”), and major parents (“Maj_num”) are studied. The correlation estimates (“Pearson_corr”) are obtained using the Pearson correlation method. The “Pval” column contains the *P* values using the Pearson test, whilst in the “P_adj” column, the FDR-adjusted (False Discovery Rate) values are presented. The significance level is explicitly stated in the “Signif_level” column based on the adjusted *P* values.

**Table S92.** The global frequency of strains and populations classified as generalists and specialists, and grouped according to the recombination-subject toxins they produce. The type of the considered toxins is stated in the “Tox_type” column, namely recombinants (“Recombinants”), minor parents (“Minor”), and major parents (“Major”). Both individual strains (“Strains”) and connected components from the strain graph (“Components”) are considered as specified in the “Comp_type” column. The “Generalist_type” column indicates the classification of the unit groups according to the number of activities. The units are called generalists in case they affect more than one host order, as defined in the “Generalist_type” column. For connected components, not less than 70% of the included isolates should be generalists to call the whole component a generalist. The “Const_change_flag” column specifies if the changes in host specificity of recombinants and parents occurred. The parent-child pairs are specified as different in the case that the lists of affected hosts for recombinants and parents are not equal.

**Table S93.** General descriptive properties of strains and connected components from the strain graph classified according to the toxins they house, the spectrum of affected host orders, and host specificity changes between toxins engaged in recombination-based domain exchanges. The “Comp_type” column indicates whether individual strains (“Strains”) or the whole connected components from the strain graph (“Components”) were used. The type of the considered toxins is stated in the “Tox_type” column, namely recombinants (“Recombinants”), minor parents (“Minor”), and major parents (“Major”). The “Generalist_type” column classifies the dataset as generalists and specialists with the former affecting more than one host order. In the case of the whole connected components, those uniting at least 70% of generalist strains are called generalists. The isolates are classified according to host specificity changes signified in the “Const_change_flag” column. The parent-child pairs are called different if the sets of host orders are not exactly equal, and the same otherwise. The estimates, including the mean, mean SD (Standard Deviation) values based on the number of activities, are presented in the “Mean_hosts”, “Median_hosts”, and “SD_hosts” columns. The same data for the number of produced toxins are described in the “Mean_toxins”, “Median_toxins”, and “SD_toxins” columns.

**Table S94.** Global comparisons between strains and bacterial populations encompassing certain toxins according to their role in recombination events. The underlying data are described in Table S89. The “Comp_type” column indicates whether individual strains (“Strains”) or the whole connected components from the strain graph (“Components”) were used. The “Generalist_type” column classifies the dataset as generalists and specialists with the former affecting more than one host order. In the case of the whole connected components, those uniting at least 70% of generalist strains are called generalists. Specificity changes are described in the “Change_flag” column, and the parent-child pair is considered different if the host orders are not completely identical. Toxin types in comparison are listed in the “Tox_type1” and “Tox_type2” columns. Recombinants (“Recombinants”), minor parents (“Minor”), and major parents (“Major”) are examined. The average number of hosts for the two groups under consideration is given in the “Num_host1” and “Num_host2” columns, whilst the number of toxins is described in the“Num_tox1” and “Num_tox2” columns. The average estimates are statistically compared using the Wilcox test with the respective *P* values specified in the “Pval_hosts” and “Pval_tox” columns. The FDR-adjusted (False Discovery Rate) *P* values are presented in the “P_adj_hosts” and “P_adj_tox” columns. The “Signif_level_hosts” and “Signif_level_tox” describe the significance level based on the adjusted estimates.

**Table S95.** The global correlation landscape between the isolates containing Cry toxins of distinct groups. Both connected components (“Components”) and strains (“Strains”) are studied as specified in the “Comp_type” column. The “Generalist_type” column gives the classification of the units according to the number of host orders. Generalists affect more than one host order. In the case of the whole connected components, those uniting at least 70% of generalist strains are called generalists. The isolates are classified according to host specificity changes signified in the “Const_change_flag” column. The parent-child pairs are called different if the sets of host orders are not exactly equal, and the same otherwise. The characteristics in comparisons within the considered isolates are presented in the “Param1” and “Param2” columns. The following data are studied, namely: the number of affected hosts for the whole unit (“Num_orders”) or recombination-affected toxins residing in it (“Num_orders_tox”), the number of toxins in the component/strain, the number of individual recombination events between the toxins residing in the units (“Num_events”), and the percentage of generalist strains/components in the considered group (“Gen_percent”). The correlation estimates (“Corr_stat”) are obtained using the Pearson correlation method. The “Pval” column contains the *P* values using the Pearson test, whilst in the “P_adj” column, the FDR-adjusted (False Discovery Rate) values are presented. The significance level is explicitly stated in the “Signif_level” column based on the adjusted *P* values.

**Table S96.** The total frequency of recombination strategies in the context of changes in the number of toxic activities. Each recombination event is classified according to the total percentage of generalist strains/connected components for those containing recombinants (“Rec_spec”), minor parents (“Min_spec”), and major parents (“Maj_spec”). The usage of strains (“Strains”) and connected components (“Components”) is displayed in the “Class” column. The content in the “Strategy” column unites the descriptions from the above-mentioned columns following the same order (recombinants-major parents-minor parents). For instance, the SSS strategy implies that the exchanges were constrained by specialists only.

**Table S97.** The number of strains/connected components from the strain graph realizing certain recombination strategies according to changes in the number of host orders. Both connected components (“Components”) and strains (“Strains”) are studied as specified in the “Comp_type” column. The type of the considered toxins is stated in the “Tox_type” column, namely recombinants (“Recombinants”), minor parents (“Minor”), and major parents (“Major”). The content in the “Strategy” column describes whether the isolates represent specialists or generalists following the predefined order (recombinants, major parents, minor parents). For instance, the SSS strategy implies that the exchanges were constrained by specialists only. Generalists affect more than one host order. In the case of the whole connected components, those uniting at least 70% of generalist strains are called generalists.

**Table S98.** The descriptive statistics of bacterial populations realizing distinct recombination strategies according to changes in the number of host orders. The data are arranged according to individual recombination events (the “ID” column) affecting toxins residing in the strains or connected components. The “Comp_type” column indicates whether individual strains (“Strains”) or the whole connected components from the strain graph (“Components”) were used. The identifier or the isolates/components is presented in the “Unit_name” column. The total number of toxins in the unit is given in the “Num_toxins” column, whereas the number of host orders for the isolates and toxins they house is presented in the “Num_hosts” and “Num_hosts_tox” columns, respectively. The “Orders_local_flag” and “Const_change_flag” columns represent changes in host specificity after recombination events, with the former describing the recombinant-parent pairs according to the type of the parents and the latter indicating the global comparison based on the sets of hosts for all parents. The pairs are considered different if the lists of affected orders are not completely identical. The toxin group within the units is presented in the “Tox_type” columns, including recombinants (“Recombinants”), minor parents (“Minor”), and major parents (“Major”). The “Final_type” column classifies the dataset as generalists and specialists with the former affecting more than one host order. This classification was used to define the whole strategy. The row is attributed to generalists if at least 70% of generalist units are present in the list of all strains/components containing toxins from a certain recombination event. At the same time, the “Unit_gen” column describes the type of the strain/component mentioned in the “Unit_name” column, per se. The fraction of generalists is presented in the “Gen_percent” column. The “Num_events” column represents the total number of individual recombination events affecting all toxins in the isolate/population. The strategy is given in the “Strategy” column. The respective content describes if the isolates represent specialists or generalists following the predefined order (recombinants, major parents, minor parents). For instance, the SSS strategy implies that the exchanges were constrained by specialists only.

**Table S99.** Common properties of the isolates and bacterial populations that realize certain recombination strategies. The “Comp_type” column indicates whether individual strains (“Strains”) or the whole connected components from the strain graph (“Components”) were used. The content in the “Strategy” column unites the descriptions from the above-mentioned columns following the same order (recombinants-major parents-minor parents). The strategy is defined by the nature of the units containing these toxin groups according to their attributions to generalists and specialists, with the former affecting more than one host order. In the case of the whole connected components, those uniting at least 70% of generalist strains are called generalists. In the strategy name, not less than 70% of all strains/components encompassing the toxins of a certain type from a certain recombination should be generalists to call the entire group a generalist. For instance, the SSS strategy implies that the exchanges were constrained by specialists only. The type of the considered toxins is stated in the “Tox_type” column, namely recombinants (“Recombinants”), minor parents (“Minor”), and major parents (“Major”). The estimates, including the mean, mean SD (Standard Deviation) values based on the number of activities for the isolates, are presented in the “Mean_hosts”, “Median_hosts”, and “SD_hosts” columns. Similarly, the number of host orders for toxins within the isolates is given in the “Mean_hosts_tox”, “Median_hosts_tox”, and “SD_hosts_tox” columns. The same data for the number of produced toxins are described in the “Mean_toxins”, “Median_toxins”, and “SD_toxins” columns. Finally, the number of individual recombination events affecting toxins residing in strains/components is given in the “Mean_events”, “Median_events”, and “SD_events” columns.

**Table S100.** The correlation characteristics between distinct factors in the isolates attributed to certain recombination strategies. The content in the “Strategy” column unites the descriptions from the above-mentioned columns following the same order (recombinants-major parents-minor parents). The strategy is defined by the nature of the units containing these toxin groups according to their attributions to generalists and specialists, with the former affecting more than one host order. In the case of the whole connected components, those uniting at least 70% of generalist strains are called generalists. In the strategy name, not less than 70% of all strains/components encompassing the toxins of a certain type from a certain recombination should be generalists to call the entire group a generalist. For instance, the SSS strategy implies that the exchanges were constrained by specialists only. The “Comp_type” column indicates whether individual strains (“Strains”) or the whole connected components from the strain graph (“Components”) were used. The toxin group within the units is presented in the “Tox_type” columns, including recombinants (“Recombinants”), minor parents (“Minor”), and major parents (“Major”). The characteristics in comparisons within the considered isolates/components are presented in the “Param1” and “Param2” columns. The following data are studied, namely: the number of affected hosts for the whole unit (“Num_orders”) or recombination-affected toxins residing in it (“Num_orders_tox”), the number of toxins in the component/strain, the number of individual recombination events between the toxins residing in the units (“Num_events”), and the percentage of generalist strains/components in the considered group (“Gen_percent”). The correlation estimates (“Corr_stat”) are obtained using the Pearson correlation method. The “Pval” column contains the *P* values using the Pearson test, whilst in the “P_adj” column, the FDR-adjusted (False Discovery Rate) values are presented. The significance level is explicitly stated in the “Signif_level” column based on the adjusted *P* values.

**Table S101.** Summary of evolutionary selection analysis using branch, site, and branch-site models. The respective evaluation is made based on domain sequences individually (“Current_domain”). In the case of site and branch-site models, recombinants or parents are marked, which corresponds to “rec_” and “per_” prefixes in columns. Mean ω values (the nonsynonymous to synonymous substitution rate ratio, dN/dS) for background and foreground are presented in columns with “omega_b” and “omega_1” postfixes. The postfix “_lnL” indicates likelihood estimates for each evolutionary model. Results of the LRT (likelihood ratio test) are presented in columns containing “_vs_” interfix. The coordinates of revealed conserved, positively selected, and relaxed sites are included in columns with “sites_c”, “sites_s”, and “sites_r” postfixed, respectively. The probability of sites for each significant region is placed in parentheses.

**Table S102.** Event-wise properties with results of evolutionary selection analysis using branch, site, and branch-site models. The respective evaluation is made based on domain sequences individually (“Current_domain”). In the case of branch and branch-site models, recombinants or parents are marked, which corresponds to “Rec” and “Par” in the “Tox_type” column. Mean ω values (the nonsynonymous to synonymous substitution rate ratio, dN/dS) for background and foreground are presented in columns with “omega_b” and “omega_1” postfixes. The columns “evol_type” and “Site_model_type” indicated the best-fit evolutionary model calculated on branches and sites. The best-fit site and branch-site models are included in columns “best_site_model” and “best_branch_site”, respectively. Columns with postfixes “_pos” and “cons” correspond to the number of significantly positively selected and conserved sites. The column “Same_flag” indicates whether at least one order differs between recombinants and parents. The overlap between sets of species affected by parents and recombinants is calculated using the Szymkiewicz-Simpson coefficient (“Simpson_species”).

**Table S103.** The distribution of sites significantly associated with evolutionary selection according to site and branch-site models. The respective evaluation is made based on domain sequences individually (“Current_domain”). In the case of branch-site models, recombinants or parents are marked, which corresponds to “Rec” and “Par” in the “Tox_type” column. For each site among the relative coordinates, the respective probabilities are presented (“Site_prob”).

**Table S104.** Taxonomic attributions of strains harboring recombinants and parents. Shown events from the refined dataset of recombination events detected with the RDP4 software (Table S12). Presented are transferred domains, recombinants (“Recs”), minor, and major parents (“Major_parents”). Columns with the “parent_” prefix represent parents based on the phylogenetic trees of the individual domains. For a detailed description of the filtering procedure, see Fig. S1a. The column “Non_bt_type” contains the lists of toxins regarding their role in recombination found in species other than *Bacillus thuringiensis*.

**SI References**

1.	Wickham H. ggplot2: elegant graphics for data analysis. Springer-Verlag New York, 2016.

2.	Yu G. Using ggtree to visualize data on tree-like structures. *Curr Protoc Bioinformatics* 2020;**69**:e96. https://doi.org/10.1002/cpbi.96

3.	Ellson J et al. Graphviz - open source graph drawing tools. In: Mutzel P, Jünger M, Leipert S (eds.) *International symposium on graph drawing*. 2002. Berlin, Heidelberg: Springer Berlin Heidelberg, 2002, pp 483–484. https://doi.org/10.1007/3-540-45848-4_57

4.	Crickmore N et al. A structure-based nomenclature for *Bacillus thuringiensis* and other bacteria-derived pesticidal proteins. *J Invertebr Pathol* 2021;**186**:107438. https://doi.org/10.1016/j.jip.2020.107438

5.	Shikov AE et al. No more tears: mining sequencing data for novel *Bt* Cry toxins with CryProcessor. *Toxins (Basel)* 2020;**12**:204. https://doi.org/10.3390/toxins12030204

6.	Fu L et al. CD-HIT: accelerated for clustering the next-generation sequencing data. *Bioinformatics* 2012;**28**:3150–3152. https://doi.org/10.1093/bioinformatics/bts565

7.	Cock PJA et al. Biopython: freely available Python tools for computational molecular biology and bioinformatics. *Bioinformatics* 2009;**25**:1422–1423. https://doi.org/10.1093/bioinformatics/btp163

8.	Shen W et al. SeqKit: a cross-platform and ultrafast toolkit for FASTA/Q file manipulation. *PLoS One* 2016;**11**:e0163962. https://doi.org/10.1371/journal.pone.0163962

9.	Quinlan AR, Hall IM. BEDTools: a flexible suite of utilities for comparing genomic features. *Bioinformatics* 2010;**26**:841–842. https://doi.org/10.1093/bioinformatics/btq033

10.	Katoh K, Standley DM. MAFFT multiple sequence alignment software version 7: improvements in performance and usability. *Mol Biol Evol* 2013;**30**:772–780. https://doi.org/10.1093/molbev/mst010

11.	Capella-Gutiérrez S, Silla-Martínez JM, Gabaldón T. trimAl: a tool for automated alignment trimming in large-scale phylogenetic analyses. *Bioinformatics* 2009;**25**:1972–1973. https://doi.org/10.1093/bioinformatics/btp348

12.	Darriba D et al. ModelTest-NG: a new and scalable tool for the selection of DNA and protein evolutionary models. *Mol Biol Evol* 2020;**37**:291–294. https://doi.org/10.1093/molbev/msz189

13.	Kozlov AM et al. RAxML-NG: a fast, scalable and user-friendly tool for maximum likelihood phylogenetic inference. *Bioinformatics* 2019;**35**:4453–4455. https://doi.org/10.1093/bioinformatics/btz305

14.	Sand A et al. tqDist: a library for computing the quartet and triplet distances between binary or general trees. *Bioinformatics* 2014;**30**:2079–2080. https://doi.org/10.1093/bioinformatics/btu157

15.	Paradis E, Claude J, Strimmer K. APE: analyses of phylogenetics and evolution in R language. *Bioinformatics* 2004;**20**:289–290. https://doi.org/10.1093/bioinformatics/btg412

16.	Schliep KP. phangorn: phylogenetic analysis in R. *Bioinformatics* 2011;**27**:592–593. https://doi.org/10.1093/bioinformatics/btq706

17.	Mir A, Rotger L, Rosselló F. Sound Colless-like balance indices for multifurcating trees. *PLoS One* 2018;**13**:e0203401–e0203401. https://doi.org/10.1371/journal.pone.0203401

18.	Galili T. dendextend: an R package for visualizing, adjusting and comparing trees of hierarchical clustering. *Bioinformatics* 2015;**31**:3718–3720. https://doi.org/10.1093/bioinformatics/btv428

19.	Mostowy R et al. Efficient inference of recent and ancestral recombination within bacterial populations. *Mol Biol Evol* 2017;**34**:1167–1182. https://doi.org/10.1093/molbev/msx066

20.	Croucher NJ et al. Rapid phylogenetic analysis of large samples of recombinant bacterial whole genome sequences using Gubbins. *Nucleic Acids Res* 2015;**43**:e15. https://doi.org/10.1093/nar/gku1196

21.	Didelot X, Wilson DJ. ClonalFrameML: efficient inference of recombination in whole bacterial genomes. *PLoS Comput Biol* 2015;**11**:1–18. https://doi.org/10.1371/journal.pcbi.1004041

22.	Gu Z et al. circlize implements and enhances circular visualization in R. *Bioinformatics* 2014;**30**:2811–2812. https://doi.org/10.1093/bioinformatics/btu393

23.	Hagberg AA, Schult DA, Swart PJ. Exploring network structure, dynamics, and function using NetworkX. *7th Python in Science Conference (SciPy 2008)* . 2008. https://doi.org/10.25080/TCWV9851

24.	Tonkin-Hill G et al. Producing polished prokaryotic pangenomes with the Panaroo pipeline. *Genome Biol* 2020;**21**:180. https://doi.org/10.1186/s13059-020-02090-4

25.	Xie Z, Tang H. ISEScan: automated identification of insertion sequence elements in prokaryotic genomes. *Bioinformatics* 2017;**33**:3340–3347. https://doi.org/10.1093/bioinformatics/btx433

26.	Starikova E V et al. Phigaro: high-throughput prophage sequence annotation. *Bioinformatics* 2020;**36**:3882–3884. https://doi.org/10.1093/bioinformatics/btaa250

27.	Bertelli C, Brinkman FSL. Improved genomic island predictions with IslandPath-DIMOB. *Bioinformatics* 2018;**34**:2161–2167. https://doi.org/10.1093/bioinformatics/bty095

28.	Zeileis A, Grothendieck G. zoo: S3 infrastructure for regular and irregular time series. *J Stat Softw* 2005;**14**:1–27. https://doi.org/10.18637/jss.v014.i06

29.	Kassambara A, Mundt F. Factoextra: extract and visualize the results of multivariate data analyses. R package version 1.0.7. 2020.

30.	Charrad M et al. NbClust: An R package for determining the relevant number of clusters in a data set. *J Stat Softw* 2014;**61**:1–36. https://doi.org/10.18637/jss.v061.i06

31.	Bailey TL et al. MEME SUITE: tools for motif discovery and searching. *Nucleic Acids Res* 2009;**37**:W202–W208. https://doi.org/10.1093/nar/gkp335

32.	Fox J, Weisberg S. Visualizing fit and lack of fit in complex regression models with predictor effect plots and partial residuals. *J Stat Softw* 2018;**87**. https://doi.org/10.18637/jss.v087.i09

33.	Roder T et al. Scoary2: rapid association of phenotypic multi-omics data with microbial pan-genomes. *Genome Biol* 2024;**25**:93. https://doi.org/10.1186/s13059-024-03233-7

34.	Frankenhuyzen K van. Insecticidal activity of *Bacillus thuringiensis* crystal proteins. *J Invertebr Pathol* 2009;**101**:1–16. https://doi.org/10.1016/j.jip.2009.02.009

35.	Valtierra-de-Luis D et al. Potential for *Bacillus thuringiensis* and other bacterial toxins as biological control agents to combat dipteran pests of medical and agronomic importance. *Toxins (Basel)* 2020;**12**:773. https://doi.org/10.3390/toxins12120773

36.	Domínguez-Arrizabalaga M et al. Insecticidal activity of *Bacillus thuringiensis* proteins against coleopteran pests. *Toxins (Basel)* 2020;**12**:430. https://doi.org/10.3390/toxins12070430

37.	Guangchuang Y. Scatterpie: scatter pie plot. 2025

38.	Huerta-Cepas J, Serra F, Bork P. ETE 3: reconstruction, analysis, and visualization of phylogenomic data. *Mol Biol Evol* 2016;**33**:1635–1638. https://doi.org/10.1093/molbev/msw046

39.	Grant JR et al. Proksee: in-depth characterization and visualization of bacterial genomes. *Nucleic Acids Res* 2023;**51**:W484-W492 https://doi.org/10.1093/nar/gkad326

40.	Hollensteiner J et al. Complete genome sequence of the nematicidal *Bacillus thuringiensis* MYBT18246. *Stand Genomic Sci* 2017;**12**:48. https://doi.org/10.1186/s40793-017-0259-x

41.	Mahadeva Swamy HM et al. Expression of *cry3A* gene and its toxicity against Asian Gray Weevil *Myllocerus undecimpustulatus undatus* Marshall (Coleoptera: Curculionidae). *J Basic Microbiol* 2013;**53**:664–676. https://doi.org/10.1002/jobm.201200272

42.	Barloy F et al. Cloning and expression of the first anaerobic toxin gene from *Clostridium bifermentans* subsp. *malaysia*, encoding a new mosquitocidal protein with homologies to *Bacillus thuringiensis delta*-endotoxins. *J Bacteriol* 1996;**178**:3099–3105. https://doi.org/10.1128/jb.178.11.3099-3105.1996

43.	Zhang J et al. Cloning and analysis of the first *cry* gene from *Bacillus popilliae*. *J Bacteriol* 1997;**179**:4336–4341. https://doi.org/10.1128/jb.179.13.4336-4341.1997

44.	Nowakowska MB, Douillard FP, Lindström M. Looking for the X factor in bacterial pathogenesis: association of *orfX-p47* gene clusters with toxin genes in clostridial and non-clostridial bacterial species. *Toxins (Basel)* 2019;**12**:19. https://doi.org/10.3390/toxins12010019

45.	Castillo-Esparza JF, Hernández-González I, Ibarra JE. Search for Cry proteins expressed by *Bacillus* spp. genomes, using hidden Markov model profiles. *3 Biotech* 2019;**9**:13. https://doi.org/10.1007/s13205-018-1533-3

46.	Shikov AE et al. Current methods for recombination detection in bacteria. *Int J Mol Sci* 2022;**23**. https://doi.org/10.3390/ijms23116257

47.	de Maagd RA, Bravo A, Crickmore N. How *Bacillus thuringiensis* has evolved specific toxins to colonize the insect world. *Trends Genet* 2001;**17**:193–199. https://doi.org/10.1016/s0168-9525(01)02237-5

48.	Bravo A et al. Evolution of *Bacillus thuringiensis* Cry toxins insecticidal activity. *Microb Biotechnol* 2013;**6**:17–26. https://doi.org/10.1111/j.1751-7915.2012.00342.x

49.	Pardo-López L, Soberón M, Bravo A. *Bacillus thuringiensis* insecticidal three-domain Cry toxins: mode of action, insect resistance and consequences for crop protection. *FEMS Microbiol Rev* 2013;**37**:3–22. https://doi.org/10.1111/j.1574-6976.2012.00341.x

50.	Song L et al. Specific activity of a *Bacillus thuringiensis* strain against *Locusta migratoria manilensis*. *J Invertebr Pathol* 2008;**98**:169–176. https://doi.org/10.1016/j.jip.2008.02.006

51.	Kitts PA et al. Assembly: a resource for assembled genomes at NCBI. *Nucleic Acids Res* 2016;**44**:D73-80. https://doi.org/10.1093/nar/gkv1226
